# Use of a targeted, computer/web-based guided self-help psychoeducation toolkit for distressing hallucinations (MUSE) in people with an at-risk mental state for psychosis: protocol for a randomised controlled feasibility trial

**DOI:** 10.1136/bmjopen-2023-076101

**Published:** 2023-06-30

**Authors:** Jahnese Hamilton, Bronia Arnott, Charlotte Aynsworth, Nicola A Barclay, Lauren Birkett, Toby Brandon, Lyndsey Dixon, Robert Dudley, J Einbeck, Christopher Gibbs, Ehsan Kharatikoopaei, Jennifer Simpson, Guy Dodgson, Charles Fernyhough

**Affiliations:** 1 Research and Development, Cumbria Northumberland Tyne and Wear NHS Foundation Trust, Newcastle upon Tyne, UK; 2 Faculty of Medical Sciences, Newcastle University, Newcastle upon Tyne, UK; 3 Central At-Risk Mental State Service, Cumbria Northumberland Tyne and Wear NHS Foundation Trust, Newcastle upon Tyne, UK; 4 Faculty of Health and Life Sciences, Northumbria University, Newcastle upon Tyne, UK; 5 Department of Psychology, University of York, York, UK; 6 Department of Psychology, Durham University, Durham, UK; 7 Department of Computing and Maths, Manchester Metropolitan University, Manchester, UK; 8 Early Intervention in Psychosis Service, Tees Esk and Wear Valleys NHS Foundation Trust, Darlington, UK

**Keywords:** MENTAL HEALTH, PSYCHIATRY, Randomized Controlled Trial

## Abstract

**Introduction:**

Individuals who access at-risk mental state (ARMS) services often have unusual sensory experiences and levels of distress that lead them to seek help. The Managing Unusual Sensory Experiences (MUSE) treatment is a brief symptom targeted intervention that draws on psychological explanations to help account for unusual experiences. Practitioners use formulation and behavioural experiments to support individuals to make sense of their experiences and enhance coping strategies. The primary objective of this feasibility trial is to resolve key uncertainties before a definitive trial and inform parameters of a future fully powered trial.

**Methods and analysis:**

88 participants aged 14–35 accepted into ARMS services, experiencing hallucinations/unusual sensory experiences which are considered by the patient to be a key target problem will be recruited from UK National Health Service (NHS) sites and randomised using 1:1 allocation (stratified by site, gender, and age) to either 6–8 sessions of MUSE or time-matched treatment as usual. Participants and therapists will be unblinded, research assessors are blinded. Blinded assessment will occur at baseline, 12 weeks and 20 weeks postrandomisation. Data will be reported in line with Consolidated Standards of Reporting Trials. Primary trial outcomes are feasibility outcomes, primary participant outcomes are functioning and hallucinations. Additional analysis will investigate potential psychological mechanisms and secondary mental well-being outcomes. Trial progression criteria follows signal of efficacy and uses an analytical framework with a traffic-light system to determine viability of a future trial. Subsequent analysis of the NHS England Mental Health Services Data Set 3 years postrandomisation will assess long-term transition to psychosis.

**Ethics and dissemination:**

This trial has received Research Ethics Committee approval (Newcastle North Tyneside 1 REC; 23/NE/0032). Participants provide written informed consent; young people provide assent with parental consent. Dissemination will be to ARMS Services, participants, public and patient forums, peer-reviewed publications and conferences.

**Trial registration number:**

ISRCTN58558617.

**STRENGTHS AND LIMITATIONS OF THIS STUDY**

- This is a feasibility randomised controlled trial, results will address key uncertainties to inform a future large-scale trial, including sample size and design decisions.
- The Managing Unusual Sensory Experiences intervention toolkit, the trial design and participant facing materials have been developed with substantial input from people with lived experience.
- This study is distinctive in exploring potential causal cognitive mechanisms in an at-risk mental state population who have unusual sensory experiences.
- There is no gold standard treatment to compare the intervention to, so controlled time-matched treatment as usual is selected as the comparator.
- The follow-up period is short (20 weeks postrandomisation), therefore, longer-term participant impacts will not be fully assessed; however, long-term transition to psychosis will be examined via the Mental Health Services Data Set.

## Introduction

At-risk mental state (ARMS) describes presentations that indicate a potential prodromal stage of psychosis, or risk of psychosis, with around 25% of ARMS individuals converting to psychosis within 36 months.^1^ The importance of working with these individuals to target possible unhelpful beliefs in development, reduce distress, support healthy functioning and potentially to prevent the development of full psychosis is widely advocated.^2 3^

The presence of unusual sensory experiences, such as hearing voices and seeing visions (hallucinations), may not in themselves indicate mental ill health as there may be common underlying psychological mechanisms or a continuum of experience from benign, everyday experiences to more severe hallucinations that require treatment.^4^ However, increased frequency and intensity of hallucinations, alongside distress and a decline in functioning, are linked to transition to psychosis and are threshold criteria in scales recommended in ARMS services.^5 6^ Intervening to reduce the distress of unusual sensory experiences and offer explanations of the possible mechanisms behind these experiences may be key in preventing transition to psychosis.^3 7^

Current UK National Institute for Health and Care (NICE) guidelines recommend that people meeting ARMS criteria should be referred for specialist assessment and offered cognitive–behavioural therapy (CBT) to reduce the risk of developing psychosis.^8–10^ While approaches involving CBT and CBT with supportive therapy show promise in ARMS, the evidence for CBT improving functioning and mental state, or reducing progression to psychosis, is inconclusive.^11–13^ No specific psychological intervention has been identified as having superior effectiveness in its treatment; there is no gold-standard treatment.^11 14 15^ ARMS services, therefore, need to further assess interventions that indicate potential benefit. Robust clinical trials are needed to determine benefits vs risk profiles, accessibility and cost-effectiveness.^11 13 16^

Treatment development would be improved if they addressed key causal mechanisms leading to distressing experiences, and adapted treatment to the needs of different age groups.^17 18^ Taking a staged or stepped approach to psychological intervention is good practice, usually with CBT and needs-based interventions prior to pharmacology.^8 18 19^ There is scope for research into briefer approaches implemented prior to CBT in ARMS services, and emerging evidence from early intervention in psychosis (EIP) research that inclusion of briefer targeted evidence-based interventions prior to CBT may result in a reduction of need for more in-depth CBT, as people better understand their experiences and have less need for interventions.^20^

Through extensive multidisciplinary research into voice hearing, clinically embedded research with patients who are indicating at risk state for psychosis, and studies of first episode psychosis, we have developed a targeted, computer/web-based guided self-help psychoeducation toolkit for distressing hallucinations (managing unusual sensory experiences, MUSE).^7 21^ MUSE endeavours to provide scientific and normalising explanations that may provide acceptable and helpful understandings of an individual’s unusual sensory experiences and help to prevent more delusional explanations from developing. MUSE has been trialled with an ARMS patient group in a non-randomised study^21^ and shown to be acceptable with good participant satisfaction with the therapy. We intend to assess MUSE through a series of trials to determine patient benefit and possible impact on progression to psychosis in patients at high risk. We will also seek to learn more about whether change relates to target mechanisms underlying hallucination subtypes.^22^ This could be important for further refinement of treatment.

### Objectives

#### Primary objective

To conduct an ISRCTN-registered feasibility randomised controlled trial to resolve key feasibility uncertainties and inform the parameters of a future trial, to investigate the preliminary effect of MUSE+treatment as usual (TAU) versus time-matched supportive psychotherapy TAU on general functioning (assessed using the Social and Occupational Functional Assessment Scale (SOFAS),^23^ and mental state related to frequency and distress of unusual sensory experiences and false beliefs (assessed using the Psychotic Symptom Rating Scales (PSYRATS)^24^ total score, and sub-scales Hallucinations and Attribution^25^) in ARMS patients post therapy and at 5 month postrandomisation follow-up.

#### Secondary objectives

To explore additional treatment effects on unusual sensory experiences, anxiety, depression and quality of life, and whether there are indications of other factors (sleep disturbance and trauma) influencing treatment effects.

To test feasibility of collecting measures of psychological mechanisms, including psychological and personal (phenotypical) factors implicated in the clinical course of hallucinations. To analyse which psychological mechanisms are influenced by the treatment and contribute to its clinical effect and inform a future investigation of whether any efficacy of MUSE is through impact on these mechanisms.

To collect routine data for a future records investigation testing feasibility of tracking transition to psychosis through medical databases (hospital records/Mental Health Services Data Set (MHSDS)), to examine which features of MUSE (presenting, treatment response and mechanistic) are most relevant to psychosis prevention.

## Methods and analysis

### Trial design and flow chart

This is a feasibility trial employing a prospective randomised, open-label, observer blinded, endpoint design assessing a targeted, computer/web based guided self-help psychoeducation toolkit for distressing hallucinations (MUSE)+TAU (6–8 sessions) compared with a TAU time-matched control (also referred to as supportive psychotherapy) (6–8 sessions) offered by a multidisciplinary team which includes needs based emotional support, psychoeducation and stress management, aiming to reduce distress from hallucinations and improve functioning, in people with an ARMS for psychosis in UK secondary care mental health services.

The trial has received National Health Service (NHS) Research Ethics positive opinion (23/NE/0032) and Health Research Authority Approvals and is registered with the ISRCTN registry (ISRCTN58558617, registered 9 May 2023). Two substantial amendments followed first approval and were obtained prior to first participant consent: Amendment 1 notably added in an unvalidated Preferences Questionnaire for therapeutic intervention, and changed an anxiety self-report questionnaire over to use the State-Trait Anxiety Inventory-Short Form (STAI-Short Form).^26–28^ Amendment 2 replaced a longer dissociative experiences questionnaire for the eight-item Brief Dissociative Experiences Scale (DES-B)-Modified.^29 30^

The trial has an independent trial steering committee (TSC) and Lived Experience Advisory Panel (LEAP) facilitated by a coapplicant for the study with lived experience of psychosis.

### Participants

Recruitment will be via NHS secondary care mental health clinical teams providing ARMS services. Patients who potentially meet the eligibility criteria for the trial, and their parent/guardian where appropriate if under 18 years, will be informed of the study by a member of their clinical team. Participants will be checked for eligibility prior to informed consent via discussion with referring teams and in the participant–researcher discussion prior to giving informed consent. Participant information sheets will be provided at least 3 days prior to the informed consent meeting. Written informed consent in adherence to principles of Good Clinical Practice will be obtained prior to participation. For participants aged 14 and 15 years old, parent/guardian informed consent with child assent will be taken; this option of assent with parent/guardian consent will also be made available to participants aged 16 and 17 years old due to their potential vulnerability and the governing UK law which classes a minor as someone who is under 18 years old. Verbal consent form will be used for participants with literacy challenges. Interpreters and translated consent forms will be available for participants who do not speak English. Participants will be given £15 honorarium for each assessment time point.

### Trial eligibility criteria

#### Inclusion criteria

- In contact with an ARMS service or accepted on an ARMS pathway by EIP services.
- Aged 14–35.
- Hallucinations/unusual sensory experiences scoring at least three on the Perceptual Abnormalities Subscale of the Comprehensive Assessment of ARMS (CAARMS).^6^
- Hallucinations considered by the patient to be a key target problem.
- Judged to have been clinically stable for the preceding 2 weeks.

#### Exclusion criteria

- intellectual disability or severe cognitive dysfunction affecting ability to engage with research materials.
- lacking capacity to give informed consent.

### Randomisation and blinding

Eligible participants who have completed baseline assessments will be randomised and subsequent assessments will be scheduled from the point of randomisation. An independent web-based randomisation service (sealedenvelope.com) will be used for the trial. Randomisation will be in the ratio 1:1 to the two groups: MUSE+TAU (intervention) or TAU time-matched control (supportive psychotherapy+TAU (control). Randomisation will be stratified by site, gender (M/F/other) and age (14–17 years/18–35 years inclusive). Randomisation allocation will be independent and dynamically generated using a randomised modified minimisation method^31^ to assure allocation concealment along with preservation of allocation ratio. Randomisation allocation is made known to the CI and site PIs, the trial coordinator(s) and the trial therapists only at the point of randomisation, by email.

Research assessors for the trial will be blind to the allocation throughout the trial. Clinicians, therapists and participants will be unblind. Trial statisticians will be partially blind; In the first instance, for the analyses and reporting of main outcomes of the trial the Statisticians will be fully blind. However, for secondary sensitivity analysis such as impact of number of MUSE sessions on effect size) and the mechanisms investigations, the statisticians will be required to view which participants received MUSE treatment.

### Assessments

Assessors blinded to trial allocation will complete participant assessments at baseline, 12 weeks postrandomisation, and 20 weeks postrandomisation (see table 1). Sociodemographic information will be collected from the participant at baseline only (CSRI questions 1–3.5 as amended for the trial^32^).

**Table 1:** Trial assessments and key participant procedures schedule

Assessments/procedures	Participant identification	Enrolment and baseline	Randomisation	Intervention weeks 1–12	12 weeks postrandomisation (±10 days)	20 weeks postrandomisation (±10 days)
Recruitment and eligibility discussions	X					
Informed consent		X				
CSRI Sociodemographic Q1-3.5		X				
Randomisation			X			
MUSE and TAU/TAU intervention				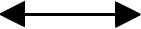		
Blinded assessments						
MUSE ARMS primary outcome measures: SOFAS and PSYRATS		X			X	X
CSRI service use Q4.1–4.4		X				
CSRI Q4.5 criminal justice services and Q5 medication		X			X	X
MUSE ARMS secondary outcome measures: CAARMS-PA, PHQ-9, GAD-7, ReQoL-20, ISI, ITQ/ITQ-CA, MMHQ		X			X	X
Subtype measures and cognitive tasks (1–2 subtypes selected per participant)		X			X	X
Treatment preference		X				
Unblinded assessments						
CSRI service use at follow-up Q4.1–4.4					X	X
Transition to psychosis data					X	X
Adverse event data					X	X
Therapeutic alliance STTS-R					X	
Participants interviews (withdrawals subsample)				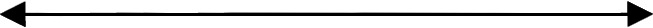		
Participants interviews (MUSE completers sub-sample)					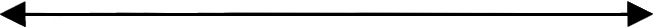	
Participants interviews (TAU subsample)					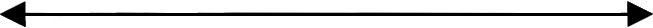	
Therapists interviews (subsample)					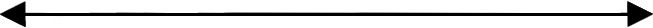	

ARMS, at-risk mental state; CAARMS-PA, Comprehensive Assessment of At-Risk Mental States subscale of Perceptual Abnormalities; CSRI, Client Services Receipt Inventory; GAD-7, Generalised Anxiety Disorder Assessment;; ISI, Insomnia Severity Index; ITQ/ITQ-CA, International Trauma Questionnaire/International Trauma Questionnaire - Child and Adolescent Version; MMHQ, Multi-Modal Hallucinations Scale; MUSE, managing unusual sensory experiences; PHQ-9, Patient Health Questionnaire; PSYRATS, Psychotic Symptom Rating Scales; ReQoL-20, Recovering Quality of Life; SOFAS, Social and Occupational Functional Assessment Scale; STTS-R, Satisfaction with Therapy and Therapist Scale-Revised.

### Primary indicators of outcome

The primary outcome measures are: (1) Feasibility outcomes, including qualitative interviews; (2) General functioning assessed using the SOFAS,^23^ a clinician/clinical researcher rated single-item scale; (3) Target problem hallucinations assessed using the PSYRATS^24^ (hallucination total) clinician/clinical researcher rated interview, and; (4) Distress and Attribution dimensions of target problem assessed using the PSYRATS.^25^

### Secondary assessments

Additional assessments will be the clinician/clinical researcher administered semi-structured interview CAARMS subscale of Perceptual Abnormalities^6^ to elicit further detail about the nature of unusual experiences. Self-reported measures will rate depression symptom severity using the Patient Health Questionnaire (PHQ-9^33^), anxiety using the Generalised Anxiety Disorder Assessment (GAD-7^34^), quality of life using the Recovering Quality of Life measure (ReQoL-20^35^), sleep difficulties using the Insomnia Severity Index (ISI^36^) and trauma using the International Trauma Questionnaire/Child and Adolescent Version (ITQ/ITQ-CA^37 38^). An unvalidated measure, the Multi-Modal Hallucinations Questionnaire (MMHQ) will be used to assess cross-modal sensory experiences.

### Service use

Assessment of potential contamination of the MUSE intervention within the TAU condition, of other psychological therapies use within the treatment arms, and the need for additional interventions beyond the treatment phase will be captured using the CSRI^32^ (as amended for the trial) at baseline, 12 weeks and 20 weeks follow-up. CSRI service use data at 12 weeks and 20 weeks will be collected from medical notes by the unblinded researcher to preserve blinding of research assessors.

### Mechanisms assessment

To assess further information on mechanisms, subtype measures and cognitive tasks will be selected per participant for 1–2 hallucination subtypes: (1) Inner speech, using the Varieties Of Inner Speech Questionnaire,^39^ and computerised cognitive tasks Auditory Signal Detection Task and Auditory Reality Monitoring Source Memory Task^40 41^; (2) Memory, using the DES-B-Modified^29 30^ and computerised cognitive task Inhibition of Currently Irrelevant Memories^42^; (3) Hypervigilance, using the STAI-Short Form,^26–28^ and computerised cognitive Jumbled Speech Task,^43 44^ and (4) Visual, using the visual section of the Plymouth Sensory Imagery Questionnaire,^45^ and computerised cognitive tasks Visual Signal Detection,^46^ Visual Reality Monitoring^47^ and Face Pareidolia Task.^46^ Researchers receive training on subtype selection. Selections are monitored and evaluated against MUSE therapist subtype selections to assess selection reliability and potential training needs.

### Acceptability assessment

To assess therapy preference, satisfaction and acceptability of the intervention, participants will be asked about treatment preferences at baseline using a study specific preferences questionnaire (see online supplemental material 1), and treatment satisfaction postintervention using the Satisfaction with Therapy and Therapist Scale-Revised (STTS-R).^48 49^ Qualitative interviews with participants and trial therapists will further explore experience of MUSE, TAU and trial procedures.

10.1136/bmjopen-2023-076101.supp1 Supplementary data

### Long-term outcomes

Long-term transition to psychosis outcomes will be collected 3 years post baseline via the NHS England MHSDS.

### Data management

Interview/clinical assessments data will be scored following the visit and entered onto Qualtrics by the researcher. Source data will be retained in the site file. Self-report data will be entered directly onto Qualtrics during visits using participant ID and visit as markers. Unblinded data on service use will be entered onto Qualtrics at the visit time points. Qualtrics outputs and computerised cognitive task data will be downloaded and date stamped at regular intervals to allow data audit. The full data set will be transferred in its anonymous form to the stats team on completion of data lock at the end of the trial. Trial monitoring at sites will occur across the life cycle of the trial and will follow the sponsor approved data monitoring plan.

### Intervention: MUSE+TAU

The MUSE intervention is a novel targeted, computer/web-based guided self-help psychoeducation toolkit and psychological treatment manual for managing distressing hallucinations in mental health, developed and owned jointly by Durham University and CNTW. Patients work with experienced therapists, under expert supervision, who use the MUSE package within therapy sessions to develop a formulation explaining the development of hallucinations and foster new skills and strategies for their management. The MUSE treatment is divided into the following modules: What are Voices?; How the Mind Works; Assessment (of participant subtype); Inner Speech; Memory and Trauma; Hypervigilance; and Sleep (see Dudley *et al* 50 for details).

Six to eight 1-hour sessions will be offered weekly by experienced therapists who are clinical psychologists or psychological therapists. Therapists will be accredited or working towards accreditation by the British Association of Behavioural and Cognitive Psychotherapists, employed by the ARMS service and have experience of MUSE, receiving clinical supervision and fortnightly MUSE supervision. MUSE is loaded onto therapists smart tablet/NHS laptop (not reliant on Wi-Fi) and is available to patients via the CNTW website between sessions. No personal data are recorded or stored on MUSE toolkit.

Session by session measures will be used as part of the MUSE package to enable therapists to monitor any variations in hallucination frequency and distress that may have a bearing on the selection of module used or revisited during the treatment session.

Therapists will be asked to complete adherence checklists for each session contained within a per-participant MUSE Therapist Pack (see online supplemental material 2). With consent, each session will be audiorecorded to enable independent review by the site Principal Investigator or delegated clinical lead of a random 10% sample to ensure fidelity to protocol within and across sites.

10.1136/bmjopen-2023-076101.supp2 Supplementary data

### Control condition: time-matched control+TAU

To control for risk of bias from an undefined comparative treatment, and potential bias from dose effects, a time-matched TAU is included.^11 51^ In order to match the comparative brief intervention to usual practice within ARMS services, components of care were identified in an engagement meeting with ARMS service leads. These common core components could be described as supportive psychotherapy or ongoing care (needs-based emotional support, psychoeducation, normalisation and stress management) and were outlined as the interventions used by therapists as part of their normal clinical toolkit, alongside routine multidisciplinary care from the team. Patients work with different therapists who are ARMS clinicians and are not trained in MUSE. These clinicians will receive supervision on their practice through the routine supervision arrangements of their service and will record the interventions used within a per-participant TAU Therapist Pack (see online supplemental material 3). We will investigate how frequently and consistently these supportive psychotherapy interventions are offered to inform whether these interventions could act as a comparator intervention in future trials. This arm will be time-matched controlled, however, variation across services precluded using this comparator being defined as a controlled intervention. Number of sessions received in this group will be recorded for analysis.

10.1136/bmjopen-2023-076101.supp3 Supplementary data

### Both groups: TAU

In addition to the trial allocated intervention (MUSE or TAU time-matched control supportive psychotherapy), both treatment groups will also receive additional usual care as clinically indicated. No treatments will be withheld on account of being part of the trial. This includes regular monitoring, signposting to appropriate local services for unmet needs, social support and crisis management when required from the multidisciplinary team. CBT is also a core intervention recommended by NICE Guidance and offered across ARMS services. However, in practice, it is not always offered to all service users. CBT may form part of the care in both conditions as part of usual care. We will investigate the number of CBT sessions received by participants in both groups and investigate whether MUSE impacts on the number of sessions required. Additional care will be based on clinical judgement and will be recorded for both arms of the study. These additional elements of care, including interventions and contacts that occur beyond the MUSE/time-matched period, will be analysed for variations and similarities in the care received between the two groups.

### Analysis

**Figure 1:**
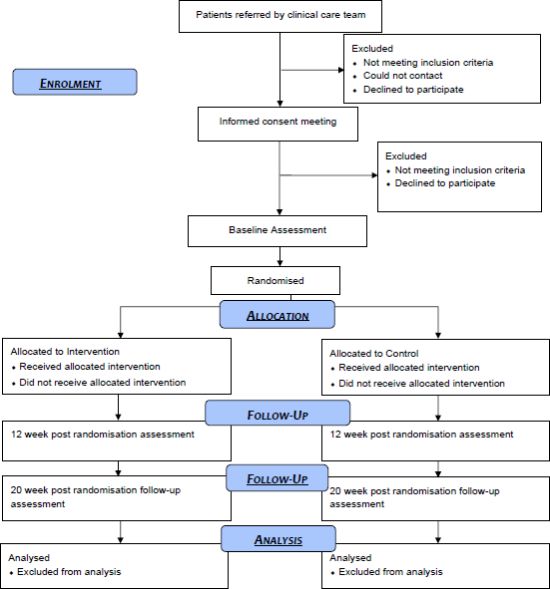
Data to report in line with the Consolidated Standards of Reporting Trials.

Analyses will follow intention to treat principles, with data analysed according to randomisation irrespective of treatment received. A full statistical analysis plan will be developed for the outcome measures and agreed with TSC before the end of data collection. Data will be reported in line with the Consolidated Standards of Reporting Trials^52^ (see figure 1).

### Sample size

As this is a feasibility trial there is no formal sample size calculation, interim analyses or stopping rule. The trial aims to recruit 88 participants with 70 participants completing the study (allowing for 20% attrition) to be able to robustly calculate the sample size for a definitive trial.^53^

### Statistical analysis

Feasibility outcomes will be analysed primarily: the ability of the trial to recruit participants, who reflect the diversity within the region and meet study inclusion criteria over the 9-month recruitment period, who complete assessment measures collected at baseline, postintervention and follow-up, until all participants complete the follow-up assessment or withdraw.

Descriptive statistics within each randomised group will be presented for baseline and follow-up points. All data will be summarised as appropriate using mean±SD and median±IQR for continuous outcome data; frequency and percentages for binary or categorical data; and rate for count data. Analysis will be via the latest version of R.

The signal of efficacy will be determined by examining the effect of each arm (MUSE vs supportive psychotherapy) on outcomes measures, estimated as change from baseline.

The effects will be estimated using generalised linear mixed effect models with the appropriate distribution and link function. Normal distributions with identity link will be used for continuous outcomes, and negative-binomial distributions with log link for count data outcomes. All binary or categorical outcomes will be analysed using generalised estimating equations (GEE). The mixed-effects models and GEE account for the repeated measurements per participant over the follow-up time points. All models will be adjusted for treatment arms and stratification variables. The mixed model approach taken will allow identifying the individual effect of the two interventions with relation to their baseline, as well as the difference in their effects through an interaction parameter of time and intervention. This can be considered as a model-based difference-in-difference analysis.

These models will be used mainly to estimate relevant parameters, since the trial is not powered for null hypothesis significance-testing. That is, while we are interested in identifying the magnitude of the signal of efficacy, we will not attempt to prove its significance.

In addition, a mediation analysis will examine how the different mechanism components mediate the estimated impact of the interventions on the primary outcomes, and a complier average causal effects analysis will determine the impact of the number of sessions on the MUSE effect.

If data are missing for a particular participant and outcome measure, this participant will be excluded from the analysis, for this outcome measure only, without further adjustment for missingness. However, the effect of missing data will be investigated additionally by sensitivity analysis using tabulation of rate of missingness across trial arms and imputation methods.

### Qualitative analysis

Audiorecordings will be transcribed and analysed (in NVivo software). Interview transcripts will be analysed using thematic analysis^54^ allowing a transparent, replicable and robust process and demonstration of reflexivity and quality. Transcripts will be coded by two researchers until coding reliability is established; coding will then be conducted by one researcher, with reliability checks by the qualitative lead. Data will be extracted into a framework matrix, summarising data by category from individual transcripts, with quotations selected as illustrative exemplars. Initial findings from the qualitative analyses will be presented to LEAP for feedback on interpretation.

### Health economics analysis

As a feasibility study, we are not undertaking a formal economic evaluation at this stage but will inform a health economic evaluation in a future definitive trial by piloting the ReQoL-UItility Index with the ReQoL-20 data for health economic analysis calculation.

### Criteria for proceeding to a future trial

The signal of efficacy is dependent on the primary outcome data (SOFAS, PSYRATS Total, PSYRATS distress, PSYRATS attribution) and follows: (1) Go: primary outcome data suggest the intervention may show an effect indicating clinical value warranting further investigation; (2) Refine: primary outcome data indicate no measure of effect, but one or more secondary outcomes indicates an effect and (3) Stop: no effect across any outcomes.

The trial progression criteria will follow signal of efficacy and cover domains of research delivery, therapy engagement and fidelity, and safety. The criteria were influenced by LEAP and TSC input and sign-off. Trial progression criteria use an analytical framework with a traffic-light system (see online supplemental table 1). Progression will depend on: (1) All Green outcomes: no/minor revisions prior to next development of the trial or (2) One or more Amber (but not Red) outcomes: If feasible, substantial alterations to the trial protocol, assessments or intervention, supported by the qualitative work-stream and discussed with the Trial Management Group and TSC prior to the next development of the trial or; (3) If one or more Red outcomes result then the trial is unlikely to progress at that site or very substantial amendments are needed. The mechanism measures and tasks will also be reviewed for sensitivity to change and reliability to inform the next development of the trial.

10.1136/bmjopen-2023-076101.supp7 Supplementary data

Decisions regarding any changes will consider the ADePT decision-making process to address potential problems with intervention, clinical setting, or trial design that may be relevant in either a trial setting or real world context. We will use qualitative data to contextualise our progression criteria, to ensure that the participant feedback informs our understanding of our research delivery and signal of efficacy.

### Adverse events

Serious adverse events (SAEs) are defined as: results in death; is life-threatening; requires hospitalisation or prolongation of existing hospitalisation; results in persistent or significant disability or incapacity; results in congenital anomaly or birth defect; or is otherwise considered medically significant by the investigator. Any SAEs shall be assessed immediately for trial relatedness and expectedness and reported to the Sponsor. Any related and unexpected SAEs and any Urgent Safety Measures (defined as: early withdrawal of participant(s) due to safety concerns about the intervention or assessments, or; changes to procedures due to concerns about staff or participant safety) shall be reported immediately to the Sponsor and Research Ethics Committee in accordance with Health Research Authority governance regulations (see: https://www.hra.nhs.uk/approvals-amendments/managing-your-approval/safety-reporting/) and sponsor standard operating procedures.

Adverse events will be recorded for all participants where the event relates to mental state, with focus on clinically significant: (A) increases in distress and/or psychosis; (B) increased harm to self/harm to others; (C) increased suicidal ideation/attempts; (D) increased use of drugs/alcohol; (E) emergency room visits for mental health concerns and(F) access to crises services.

### Patient and public involvement

The MUSE intervention, the trial design and participant facing materials and the grant application have benefited from input by individuals with lived experience. To ensure a retained focus on patients, an LEAP led by a coapplicant with personal lived experience of psychosis was established and meets monthly in a mixture of online and face-to-face formats throughout the lifetime of the trial. The study-specific preferences questionnaire was collaboratively developed with the LEAP. The outcome measures and the topic guides were piloted with LEAP members and amended following feedback. The LEAP members were consulted on the potential ethical issues of the trial and the trial progression criteria. Members of the LEAP group will also cofacilitate qualitative interviews, help disseminate study findings and enable patient experience to inform design of future research and any revisions of the treatment. Two LEAP members are part of the TSC, with one taking a lead on trial procedures and the other on the inclusion of underserved groups. Compensation for work done is given in accordance with NIHR PPI guidelines (https://www.nihr.ac.uk/documents/ppi-patient-and-public-involvement-resources-for-applicants-to-nihr-research-programmes/23437).

### Ethics and dissemination

This trial has obtained NHS Research Ethics Committee (REC) positive opinion from Newcastle North Tyneside 1 REC (reference: 23/NE/0032), and UK Health Research Authority approval (IRAS project ID: 323903). Participants are provided with participant information at least 3 days prior to providing informed consent. Participants provide written informed consent; young people provide assent with parental consent (see online supplemental materials 4–6). The research sponsor is Cumbria, Northumberland, Tyne and Wear NHS Foundation Trust (CNTW).

10.1136/bmjopen-2023-076101.supp4 Supplementary data

10.1136/bmjopen-2023-076101.supp5 Supplementary data

10.1136/bmjopen-2023-076101.supp6 Supplementary data

An anonymised version of the main outcome quantitative data and mechanisms data will be available either in open access as encouraged by peer-review publications or from the trial team on reasonable request with publication of the trial outcomes paper and mechanisms paper.

The research outcomes shall be submitted for peer-review open access publications. Anonymised data will be made available in a repository. Trial outcomes, mechanisms evaluations and long-term outcomes will be reported on.

The trial outcomes: The feasibility trial outcomes will report on feasibility outcomes and the candidate primary outcome measures (SOFAS and PSYRATS). Secondary reporting will detail the secondary treatment effects and influence of moderators. Additional reporting will detail treatment integrity: data on treatment adherence to the model (sessions checklist data); exposure of participants to the interventions and additional treatments within usual care (CSRI data); the quality of treatment delivered and responsiveness of participants as reflected on by therapists and participants (STTS-R data, qualitative data); and the programme differentiation between the novel intervention arm and the usual care arm (CSRI data).

Mechanisms will be reported on the analysis of secondary assessments for the purposes of informing which aspects of patient presentation the MUSE intervention works with, and informing the outcome measures in a future efficacy and mechanisms trial.

Long-term transition to psychosis paper: Long-term transition to psychosis through the MHSDS/medical records exploratory feasibility analysis will report which features of MUSE (presentation, treatment response, mechanistic) are indicated as most relevant to psychosis prevention.

### Trial status

The trial opened to recruitment at the two planned NHS sites on 14 April 2023 (Cumbria, Northumberland, Tyne and Wear NHS Foundation Trust) and 21 April 2023 (Tees, Esk and Wear alley NHS Foundation Trust). First participant randomisation (enrolment) was on 10 May 2023. Final participant facing procedures are due to be completed by end of June 2024. The study will finish at NHS research sites after the final assessment with the final participant is completed and the monitoring close-out visit has occurred at site.

## Supplementary Material

Reviewer comments

Author's manuscript
