# Supplementary material for: Use of a targeted, computer/web-based guided self-help psychoeducation toolkit for distressing hallucinations (MUSE) in people with an at-risk mental state for psychosis: protocol for a randomised controlled feasibility trial
Source: BMJ Open. 2023 Jun 30;13(6):e076101. doi: 10.1136/bmjopen-2023-076101 (PMC10314705; doi:10.1136/bmjopen-2023-076101)
Supplement: Supplementary data [file bmjopen-2023-076101supp001.pdf]

MUSE ARMS Feasibility Trial: Preferences Questionnaire; v1.0, 12.03.2023
IRAS Project ID: 323903

Preferences for psychological therapy or support

This set of questions is seeking to develop an understanding of your preferences for therapy treatment.

1. Do you have a preference for the number of therapy sessions you might get?

◯ 1-3

◯ 4-8

◯ 9-16

◯ 17-30

◯ Don't know

2. How important is it that your treatment:

Includes being give medication?

◯ Not important

◯ Somewhat important

◯ Very important

Includes a talking therapy?

◯ Not important

◯ Somewhat important

◯ Very important

Addresses any feelings of anxiety?

◯ Not important

◯ Somewhat important

◯ Very important

Hamilton J, *et al. BMJ Open* 2023; 13:e076101. doi: 10.1136/bmjopen-2023-076101

MUSE ARMS Feasibility Trial: Preferences Questionnaire; v1.0, 12.03.2023
IRAS Project ID: 323903

Addresses any feeling of low mood?

- ◯ Not important
- ◯ Somewhat important
- ◯ Very important

Helps you understand the causes of any unusual sensory experiences, such as hearing a voice?

- ◯ Not important
- ◯ Somewhat important
- ◯ Very important

Helps you learn to manage any unusual sensory experiences?

- ◯ Not important
- ◯ Somewhat important
- ◯ Very important

Helps you feel less distressed about any unusual sensory experiences?

- ◯ Not important
- ◯ Somewhat important
- ◯ Very important

3. What are your preferences for the way the therapist/clinical care team works with you?
   Please rate how important you think the following statements are:

I am given space to talk and feel heard

- ◯ Not important
- ◯ Somewhat important
- ◯ Very important

MUSE ARMS Feasibility Trial: Preferences Questionnaire; v1.0, 12.03.2023
IRAS Project ID: 323903

I work with my therapist to help me make sense of my experiences

- ◯ Not important
- ◯ Somewhat important
- ◯ Very important

I am involved in setting my own goals

- ◯ Not important
- ◯ Somewhat important
- ◯ Very important

I am given new ideas of how to cope with my experiences

- ◯ Not important
- ◯ Somewhat important
- ◯ Very important

4. How much do you hope to get the MUSE therapy?

- ◯ I would prefer to be allocated to MUSE based therapy
- ◯ I don’t mind one way or the other whether I receive MUSE based therapy
- ◯ I would prefer to be allocated to the treatment as usual.

Hamilton J, *et al. BMJ Open* 2023; 13:e076101. doi: 10.1136/bmjopen-2023-076101
