# Supplementary material for: Use of a targeted, computer/web-based guided self-help psychoeducation toolkit for distressing hallucinations (MUSE) in people with an at-risk mental state for psychosis: protocol for a randomised controlled feasibility trial
Source: BMJ Open. 2023 Jun 30;13(6):e076101. doi: 10.1136/bmjopen-2023-076101 (PMC10314705; doi:10.1136/bmjopen-2023-076101)
Supplement: Supplementary data [file bmjopen-2023-076101supp002.pdf]

MUSE THERAPY
THERAPIST PACK

*Return to research site file for archiving after completion

MUSE Therapist Pack Version 1.0; Date: 02.03.2023 MUSE ARMS Feasibility Trial; IRAS ID: 323903

Adherence Checklist: MUSE Therapy Sessions
(Please tick topic used in any session)

**Insert Date**:									
**Insert length of session (minutes):**									
**Module/Topic**	**S1**	**S2**	**S3**	**S4**	**S5**	**S6**	**S7**	**S8**	**Comments**
**What are voices?**									
What are voices?									
How many people hear voices?									
Why does it become a problem?									
Can things get better?									
Personal experiences									
1. **How the mind works?**									
Thoughts and senses									
How thoughts work									
Embarrassing thoughts									
The power of attention									
How we use expectation									
2. **Assessment**									
Types of unusual sensory experiences.									
What kind of voices do we hear?									
3. **Inner Speech**									
What is inner speech?									
Our inner speech can do amazing things									
Why do people not recognise voices?									
Thoughts are hard to control									
Blocking the loop									
Inner speech – what is the evidence?									
Tracking the self – Was that me?									
Writers and voice hearing									
Imaginary friends									
Formulation									
Voices and Relationships									
Transforming the voice									
Testing out your explanations									

Completed by

Participant ID (MUSE ID Number):	Print Name:	Role:	Signature:	**Completed on (DD/MM/YYYY)**

Living well with voices									
4. **Memory Based Voices**									
Memory, dissociation, trauma									
The importance of trauma									
Threat system and Soothing system									
Formulation									
Treating trauma									
5. **Hypervigilance**									
Nature versus Nurture									
Filling in the gaps									
What our perception system is designed to do									
Response to danger									
Formulation									
Threat system and soothing system									
Mistrust									
6. **Seeing Visions**									
Is seeing believing?									
What do your visions mean to you?									
Perception system design									
Filling in the gaps									
Tracking the self – was that me?									
Imaginary friends									
Testing distressing appraisals									
Changing images									
Living well with visual experiences									
Voices, visions and relationships									
Challenging unacceptability									
Testing out your explanations									
Living well with voices and visions									
7. **Sleep**									
Why do we sleep?									
How to sleep well									

	**Completed by**			
Participant ID (MUSE ID Number):	Print Name:	Role:	Signature:	**Completed on (DD/MM/YYYY)**

MUSE Therapist Pack Version 1.0; Date: 02.03.2023 MUSE ARMS Feasibility Trial; IRAS ID: 323903

Adverse Events Checklist

Insert date:									
	**S1**	**S2**	**S3**	**S4**	**S5**	**S6**	**S7**	**S8**	**Comments**
**Adverse events of interest reported?** **Add to Rio/Paris**									
**Serious Adverse Event? NB 24hour reporting deadline**									
**Urgent Safety Measures? NB Phone PI immediately**									

Adverse Events Guidance:

Adverse Events. Record on Rio/Paris for collection by the Unblinded Researcher at the 12wk and 20wk assessment time points that pertain to the following events of Protocol Interest:

- Clinically significant increases in distress and/or psychosis
- Increased harm to self/harm to others
- Increased suicidal ideation/attempts
- Increased use of drugs/alcohol
- Emergency room visits for mental health concerns
- Access to crises services

Serious Adverse Event (SAE): The site Principal Investigator (PI), or delegate shall report all SAEs within 24 hours of becoming aware of the event to the Chief Investigator (CI), or delegate via email to MUSE.ARMS@cntw.nhs.uk using the SAE reporting form. These are events that:

- results in death;
- is life-threatening;
- requires hospitalisation or prolongation of existing hospitalisation;
- results in persistent or significant disability or incapacity;
- consists of a congenital anomaly or birth defect; or,
- is otherwise considered medically significant by the investigator.

Urgent Safety Measures: The site Principal Investigator (PI), or delegate, must inform the CI immediately by telephone (Tel. 01670844670 / alternatively Teams video/voice call for guy.dodgson@cntw.nhs.uk) of urgent safety measures defined above in section 11.5 (early withdrawal/changes to procedure due to safety concerns for staff or participants). This information shall be documented on the Urgent safety reporting form and submitted by email to MUSE.ARMS@cntw.nhs.uk. This is when the following applies:

- Early withdrawal of participant(s) due to safety concerns about the intervention or assessments
- Changes to procedures due to concerns about staff or participant safety

Transition to Psychosis Checklist

Insert date:									
	**S1**	**S2**	**S3**	**S4**	**S5**	**S6**	**S7**	**S8**	**Comments**
**Indication of Transition to Psychosis?** **Add note to Rio/Paris**									

Transition to Psychosis Guidance: The following information suggests potential transition to psychosis for this protocol:

- Clinical diagnosis using standard diagnostic classification systems DSM/ICD
- Clinical diagnosis using ARMS assessment schedule documented in clinical notes
- Transfer to the Early Intervention in Psychosis pathway
- Treated or untreated psychotic episode of one week's duration or longer
- Initiation of treatment with antipsychotics (3 or more weeks of treatment with antipsychotics at a dose of ≥ 5mg haloperidol or equivalent)

Completed by

Participant ID (MUSE ID Number):	Print Name:	Role:	Signature:	**Completed on (DD/MM/YYYY)**

Therapy Session measure

MUSE Therapist Pack MUSE ARMS Feasibility Trial; IRAS ID: 323903

Session Number:

Date:

Therapy Session measure

Please answer the following questions about the voices you experienced <u>in the past week</u>

How frequent were the voices?

0%	10	20	30	40	50	60	70	80	90	100%
Voices not present					Once a day					Voices always present

Were the voices distressing? How much of the time?

0%	10	20	30	40	50	60	70	80	90	100%
Voices never distressing					Voices were distressing about half of the times					Voices always distressing

If relevant please answer the following questions about the visions you experienced <u>in the past week</u>

How frequent were the visions?

0%	10	20	30	40	50	60	70	80	90	100%
Visions not present					Once a day					Visions always present

Were the vision distressing? How much of the time?

0%	10	20	30	40	50	60	70	80	90	100%
Visions never distressing					Moderately distressing					extremely distressing

Overall, how distressing were the experiences listed above?

0%	10	20	30	40	50	60	70	80	90	100%
not at all distressing					Moderately distressing					extremely distressing

	Completed by			
Participant ID (MUSE ID Number):	Print Name:	Role:	Signature:	**Completed on (DD/MM/YYYY)**

Therapy Session measure

MUSE Therapist Pack MUSE ARMS Feasibility Trial; IRAS ID: 323903

Session Number:

Date:

Therapy Session measure

Please answer the following questions about the voices you experienced <u>in the past week</u>

How frequent were the voices?

0%	10	20	30	40	50	60	70	80	90	100%
Voices not present					Once a day					Voices always present

Were the voices distressing? How much of the time?

0%	10	20	30	40	50	60	70	80	90	100%
Voices never distressing					Voices were distressing about half of the times					Voices always distressing

If relevant please answer the following questions about the visions you experienced <u>in the past week</u>

How frequent were the visions?

0%	10	20	30	40	50	60	70	80	90	100%
Visions not present					Once a day					Visions always present

Were the vision distressing? How much of the time?

0%	10	20	30	40	50	60	70	80	90	100%
Visions never distressing					Moderately distressing					extremely distressing

Overall, how distressing were the experiences listed above?

0%	10	20	30	40	50	60	70	80	90	100%
not at all distressing					Moderately distressing					extremely distressing

	**Completed by**			
Participant ID (MUSE ID Number):	Print Name:	Role:	Signature:	**Completed on (DD/MM/YYYY)**

Therapy Session measure

MUSE Therapist Pack MUSE ARMS Feasibility Trial; IRAS ID: 323903

Session Number:

Date:

Therapy Session measure

Please answer the following questions about the voices you experienced <u>in the past week</u>

<u>How frequent were the voices?</u>

0%	10	20	30	40	50	60	70	80	90	100%
Voices not present					Once a day					Voices always present

<u>Were the voices distressing? How much of the time?</u>

0%	10	20	30	40	50	60	70	80	90	100%
Voices never distressing					Voices were distressing about half of the times					Voices always distressing

If relevant please answer the following questions about the visions you experienced <u>in the past week</u>

<u>How frequent were the visions?</u>

0%	10	20	30	40	50	60	70	80	90	100%
Visions not present					Once a day					Visions always present

<u>Were the vision distressing? How much of the time?</u>

0%	10	20	30	40	50	60	70	80	90	100%
Visions never distressing					Moderately distressing					extremely distressing

<u>Overall, how distressing were the experiences listed above?</u>

0%	10	20	30	40	50	60	70	80	90	100%
not at all distressing					Moderately distressing					extremely distressing

	Completed by			
Participant ID (MUSE ID Number):	Print Name:	Role:	Signature:	**Completed on (DD/MM/YYYY)**

Therapy Session measure

MUSE Therapist Pack MUSE ARMS Feasibility Trial; IRAS ID: 323903

Session Number:

Date:

Therapy Session measure

Please answer the following questions about the voices you experienced <u>in the past week</u>

How frequent were the voices?

0%	10	20	30	40	50	60	70	80	90	100%
Voices not present					Once a day					Voices always present

Were the voices distressing? How much of the time?

0%	10	20	30	40	50	60	70	80	90	100%
Voices never distressing					Voices were distressing about half of the times					Voices always distressing

If relevant please answer the following questions about the visions you experienced <u>in the past week</u>

How frequent were the visions?

0%	10	20	30	40	50	60	70	80	90	100%
Visions not present					Once a day					Visions always present

Were the vision distressing? How much of the time?

0%	10	20	30	40	50	60	70	80	90	100%
Visions never distressing					Moderately distressing					extremely distressing

Overall, how distressing were the experiences listed above?

0%	10	20	30	40	50	60	70	80	90	100%
not at all distressing					Moderately distressing					extremely distressing

	Completed by			
Participant ID (MUSE ID Number):	Print Name:	Role:	Signature:	**Completed on (DD/MM/YYYY)**

Therapy Session measure

MUSE Therapist Pack MUSE ARMS Feasibility Trial; IRAS ID: 323903

Session Number:

Date:

Therapy Session measure

Please answer the following questions about the voices you experienced <u>in the past week</u>

How frequent were the voices?

0%	10	20	30	40	50	60	70	80	90	100%
Voices not present					Once a day					Voices always present

Were the voices distressing? How much of the time?

0%	10	20	30	40	50	60	70	80	90	100%
Voices never distressing					Voices were distressing about half of the times					Voices always distressing

If relevant please answer the following questions about the visions you experienced <u>in the past week</u>

How frequent were the visions?

0%	10	20	30	40	50	60	70	80	90	100%
Visions not present					Once a day					Visions always present

Were the vision distressing? How much of the time?

0%	10	20	30	40	50	60	70	80	90	100%
Visions never distressing					Moderately distressing					extremely distressing

Overall, how distressing were the experiences listed above?

0%	10	20	30	40	50	60	70	80	90	100%
not at all distressing					Moderately distressing					extremely distressing

	**Completed by**			
Participant ID (MUSE ID Number):	Print Name:	Role:	Signature:	**Completed on (DD/MM/YYYY)**

Therapy Session measure

MUSE Therapist Pack MUSE ARMS Feasibility Trial; IRAS ID: 323903

Session Number:

Date:

Therapy Session measure

Please answer the following questions about the voices you experienced <u>in the past week</u>

<u>How frequent were the voices?</u>

0%	10	20	30	40	50	60	70	80	90	100%
Voices not present					Once a day					Voices always present

<u>Were the voices distressing? How much of the time?</u>

0%	10	20	30	40	50	60	70	80	90	100%
Voices never distressing					Voices were distressing about half of the times					Voices always distressing

If relevant please answer the following questions about the visions you experienced <u>in the past week</u>

<u>How frequent were the visions?</u>

0%	10	20	30	40	50	60	70	80	90	100%
Visions not present					Once a day					Visions always present

<u>Were the vision distressing? How much of the time?</u>

0%	10	20	30	40	50	60	70	80	90	100%
Visions never distressing					Moderately distressing					extremely distressing

<u>Overall, how distressing were the experiences listed above?</u>

0%	10	20	30	40	50	60	70	80	90	100%
not at all distressing					Moderately distressing					extremely distressing

Participant ID (MUSE ID Number):	Completed by<br>Print Name:	Role:	Signature:	**Completed on (DD/MM/YYYY)**

Therapy Session measure

MUSE Therapist Pack MUSE ARMS Feasibility Trial; IRAS ID: 323903

Session Number:

Date:

Therapy Session measure

Please answer the following questions about the voices you experienced <u>in the past week</u>

<u>How frequent were the voices?</u>

0%	10	20	30	40	50	60	70	80	90	100%
Voices not present					Once a day					Voices always present

<u>Were the voices distressing? How much of the time?</u>

0%	10	20	30	40	50	60	70	80	90	100%
Voices never distressing					Voices were distressing about half of the times					Voices always distressing

If relevant please answer the following questions about the visions you experienced <u>in the past week</u>

<u>How frequent were the visions?</u>

0%	10	20	30	40	50	60	70	80	90	100%
Visions not present					Once a day					Visions always present

<u>Were the vision distressing? How much of the time?</u>

0%	10	20	30	40	50	60	70	80	90	100%
Visions never distressing					Moderately distressing					extremely distressing

<u>Overall, how distressing were the experiences listed above?</u>

0%	10	20	30	40	50	60	70	80	90	100%
not at all distressing					Moderately distressing					extremely distressing

	Completed by			
Participant ID (MUSE ID Number):	Print Name:	Role:	Signature:	**Completed on (DD/MM/YYYY)**

Therapy Session measure

MUSE Therapist Pack MUSE ARMS Feasibility Trial; IRAS ID: 323903

Session Number:

Date:

Therapy Session measure

Please answer the following questions about the voices you experienced <u>in the past week</u>

<u>How frequent were the voices?</u>

0%	10	20	30	40	50	60	70	80	90	100%
Voices not present					Once a day					Voices always present

<u>Were the voices distressing? How much of the time?</u>

0%	10	20	30	40	50	60	70	80	90	100%
Voices never distressing					Voices were distressing about half of the times					Voices always distressing

If relevant please answer the following questions about the visions you experienced <u>in the past week</u>

<u>How frequent were the visions?</u>

0%	10	20	30	40	50	60	70	80	90	100%
Visions not present					Once a day					Visions always present

<u>Were the vision distressing? How much of the time?</u>

0%	10	20	30	40	50	60	70	80	90	100%
Visions never distressing					Moderately distressing					extremely distressing

<u>Overall, how distressing were the experiences listed above?</u>

0%	10	20	30	40	50	60	70	80	90	100%
not at all distressing					Moderately distressing					extremely distressing

	Completed by			
Participant ID (MUSE ID Number):	Print Name:	Role:	Signature:	**Completed on (DD/MM/YYYY)**
