# Supplementary material for: Use of a targeted, computer/web-based guided self-help psychoeducation toolkit for distressing hallucinations (MUSE) in people with an at-risk mental state for psychosis: protocol for a randomised controlled feasibility trial
Source: BMJ Open. 2023 Jun 30;13(6):e076101. doi: 10.1136/bmjopen-2023-076101 (PMC10314705; doi:10.1136/bmjopen-2023-076101)
Supplement: Supplementary data [file bmjopen-2023-076101supp003.pdf]

TAU THERAPY THERAPIST PACK

*Return to research site file for archiving after completion

TAU Therapist Pack Version 1.0; Date: 02.03.2023 MUSE ARMS Feasibility Trial; IRAS ID: 323903

Adherence Checklist: TAU Therapy Sessions (Please tick for used in any session)

**Insert Date:**									
**Insert length of session (minutes):**									
	**S1**	**S2**	**S3**	**S4**	**S5**	**S6**	**S7**	**S8**	**Comments**
**Was this a CBT session (Y/N)?**									
CBT Assessment									
Formulation									
Needs based emotional support									
Social Support									
Normalisation									
Stress management									
Psychoeducation* **Please describe if related to managing unusual sensory experiences in the comments box*									
Other:									
Other:									
Other:									

	**Completed by**			
Participant ID (MUSE ID Number):	Print Name:	Role:	Signature:	**Completed on (DD/MM/YYYY)**

TAU Therapist Pack Version 1.0; Date: 02.03.2023 MUSE ARMS Feasibility Trial; IRAS ID: 323903

Adverse Events Checklist

Insert date:									
	**S1**	**S2**	**S3**	**S4**	**S5**	**S6**	**S7**	**S8**	**Comments**
**Adverse events of interest reported?** **Add to Rio/Paris**									
**Serious Adverse Event? NB 24hour reporting deadline**									
**Urgent Safety Measures? NB Phone PI immediately**									

Adverse Events Guidance:

Adverse Events. Record on Rio/Paris for collection by the Unblinded Researcher at the 12wk and 20wk assessment time points that pertain to the following events of Protocol Interest:

- Clinically significant increases in distress and/or psychosis
- Increased harm to self/harm to others
- Increased suicidal ideation/attempts
- Increased use of drugs/alcohol
- Emergency room visits for mental health concerns
- Access to crises services

Serious Adverse Event (SAE): The site Principal Investigator (PI), or delegate shall report all SAEs within 24 hours of becoming aware of the event to the Chief Investigator (CI), or delegate via email to MUSE.ARMS@cntw.nhs.uk using the SAE reporting form. These are events that:

- results in death;
- is life-threatening;
- requires hospitalisation or prolongation of existing hospitalisation;
- results in persistent or significant disability or incapacity;
- consists of a congenital anomaly or birth defect; or,
- is otherwise considered medically significant by the investigator.

Urgent Safety Measures: The site Principal Investigator (PI), or delegate, must inform the CI immediately by telephone (Tel. 01670844670 / alternatively Teams video/voice call for guy.dodgson@cntw.nhs.uk) of urgent safety measures defined above in section 11.5 (early withdrawal/changes to procedure due to safety concerns for staff or participants). This information shall be documented on the Urgent safety reporting form and submitted by email to MUSE.ARMS@cntw.nhs.uk. This is when the following applies:

- Early withdrawal of participant(s) due to safety concerns about the intervention or assessments
- Changes to procedures due to concerns about staff or participant safety

Transition to Psychosis Checklist

Insert date:									
	**S1**	**S2**	**S3**	**S4**	**S5**	**S6**	**S7**	**S8**	**Comments**
**Indication of Transition to Psychosis?** **Add note to Rio/Paris**									

Transition to Psychosis Guidance: The following information suggests potential transition to psychosis for this protocol:

- Clinical diagnosis using standard diagnostic classification systems DSM/ICD
- Clinical diagnosis using ARMS assessment schedule documented in clinical notes
- Transfer to the Early Intervention in Psychosis pathway
- Treated or untreated psychotic episode of one week's duration or longer
- Initiation of treatment with antipsychotics (3 or more weeks of treatment with antipsychotics at a dose of ≥ 5mg haloperidol or equivalent

	**Completed by**			
Participant ID (MUSE ID Number):	Print Name:	Role:	Signature:	**Completed on (DD/MM/YYYY)**
