# Supplementary material for: Use of a targeted, computer/web-based guided self-help psychoeducation toolkit for distressing hallucinations (MUSE) in people with an at-risk mental state for psychosis: protocol for a randomised controlled feasibility trial
Source: BMJ Open. 2023 Jun 30;13(6):e076101. doi: 10.1136/bmjopen-2023-076101 (PMC10314705; doi:10.1136/bmjopen-2023-076101)
Supplement: Supplementary data [file bmjopen-2023-076101supp007.pdf]

Supplementary Table 1. Approved trial monitoring and progression criteria to a future definitive trial

Criterion	Critical feasibility outcome	Other feasibility and acceptability data relevant to the criterion		Proposed thresholds on critical outcome
**1) Recruitment**	• Number of participants consented into the trial and randomised	• Number of referrals per month<br>• Source of recruitment<br>• Number of participants eligible,<br>• Number of participants referred<br>• Reasons for non-eligibility or withdrawal of interest		Feasibility will be demonstrated where an average of least 7.84 participants are recruited and randomised per month (80% of recruitment target met).<br>If at least 5.88 participants are recruited per month, then a future trial will be feasible but additional strategies must be identified to support recruitment (e.g. informed by other feasibility data relevant to this criterion) (60-80% of recruitment target met).<br>If an average of under 5.88 participant is recruited per month over the recruitment period, feasibility within the current design will not be demonstrated (under 60% of recruitment target met).
**2) Therapy engagement**	• % who drop-out of therapy	• Session record forms for each therapy session<br>• Number of therapy sessions attended<br>• Qualitative interviews with SU participants<br>• Therapy satisfaction scores		Feasibility will be demonstrated if at least 80% of the participants in the intervention arm completed at least 4 out of the 6-8 sessions of MUSE.<br>If 60-80% of participants in the intervention arm complete at least 4 out of the 6-8 sessions of MUSE.<br>If less than 60% of participants in the intervention arm complete at least 4 out of the 6-8 sessions of MUSE.
**3) Assessment retention**	• % of participants who are lost to follow-up at primary assessment endpoint (12weeks post randomisation)	• Reasons for withdrawal from the study<br>• Qualitative interviews with SU participants<br>• Data completion		If at least 70% of participants complete primary outcome measure at primary assessment endpoint, feasibility will be demonstrated.<br>If 50-70% of participants complete primary outcome measure at primary assessment endpoint, a future trial will be feasible if strategies to overcome barriers are identified (e.g. via other data relevant to this).<br>If less than 50% of participants complete primary outcome measure at primary assessment endpoint, feasibility within the current design will not be demonstrated.
**4) Therapy fidelity**	• Adherence ratings from therapy tapes	• Session record form for each therapy session (including reasons for deviation from protocol)		Feasibility will be demonstrated if over 80% of rated therapy tapes are rated as acceptable.<br>If 50-80% of rated therapy tapes are rated as acceptable, a future trial will be feasible if strategies to overcome barriers are identified<br>If less than 50% of rated therapy tapes will be rates as acceptable, feasibility within the current design will not be demonstrated.
**5) Safety**	• Number of related SAEs	• Increased number of AEs in Intervention condition		0-1 Related SAEs in the Intervention arm.<br>2 Related SAEs in the Intervention arm.<br>3+ Related SAEs in the Intervention arm.
