# Supplementary material for: Use of a targeted, computer/web-based guided self-help psychoeducation toolkit for distressing hallucinations (MUSE) in people with an at-risk mental state for psychosis: protocol for a randomised controlled feasibility trial
Source: BMJ Open. 2023 Jun 30;13(6):e076101. doi: 10.1136/bmjopen-2023-076101 (PMC10314705; doi:10.1136/bmjopen-2023-076101)
Supplement: Supplementary data [file bmjopen-2023-076101supp004.pdf]

[Insert local site logos here]
IRAS Number: 323903 Study Title: MUSE ARMS Feasibility Trial.
Informed Consent Form [Version 2.0 23022023]
Centre Name: [e.g. CNTW / Other NHS Participating Organisation]

NHS
Cumbria, Northumberland, Tyne and Wear
NHS Foundation Trust

Participant ID Number:

MUSE ARMS Feasibility Trial

INFORMED CONSENT FORM

		Initial box to agree
1	I confirm that I have read the information sheet dated.................... (Version............) for the above study. I have had the opportunity to consider the information, ask questions and have had these answered satisfactorily.	☐
2	I understand that my participation is voluntary and that I am free to withdraw at any time without giving any reason, without my medical care or legal rights being affected.	☐
3	I understand that if I withdraw from the study, or need to be withdrawn due to becoming too unwell, the research team will keep the research data about me that they already have, and if you give consent to question 10 on this form they will continue to track long term outcomes via the MHSDS/medical notes unless you request that they do not.	☐
4	I understand that relevant sections of my medical notes and data collected during the study, may be looked at by responsible individuals from *[Research site]* and from the research Sponsor CNTW NHS Foundation Trust, their representatives and regulatory authorities for the purposes of this research study, which includes audit and monitoring for research quality assurance. I give permission for these individuals to have access to my records in accordance with this study participant information sheet and informed consent.	☐
5	I understand and agree that the information collected from me in the course of this study will be held and maintained by *[enter name of organisation(s) that will be storing the participant data]* and CNTW, and archived at *[enter name of organisation(s)]* and CNTW.	☐
6	I agree to my NHS Care Team being informed of my participation in the study.	☐
7	I agree for a brief summary of the research assessments and any treatment sessions to be shared with my clinical team (i.e. added into my NHS care notes).	☐
8	OPTIONAL: I consent to the use of audio recording of my treatment sessions to check the quality of the MUSE treatment. I understand recordings will follow NHS data security standards for storage and will be destroyed once they are checked for treatment quality. **Circle decision: YES / NO**	☐

Print double sided. When completed: One for participant, one for Investigator Site File, and one to be kept in medical notes.

9	OPTIONAL: I consent to take part in an interview about my experience in the trial. I recognise not everyone is asked to do this and that I can change my mind at any time. I am aware that these reflective interviews are audio recorded anonymously (using an ID code as identifier) and then transcribed during which any further potential personal identifying information is removed ahead of analysis of research findings.<br>**Circle decision: YES / NO**	☐
10	OPTIONAL: I consent to my medical records being accessed by the central research team at CNTW to collect follow-up data from medical databases to look at long term outcomes including use of hospital inpatient services. Medical databases include Hospital Records, and the Mental Health Services Data Set (MHSDS). This requires a copy of my consent form and my NHS record number to be sent securely to CNTW for processing and storage in the trial master file, which I agree to.<br>**Circle decision: YES / NO**	☐
11	I understand that the information collected about me will be used to support the writing up of research findings. The data in an anonymised format will be shared with researchers for this study who have a role in analysing and writing up data.	☐
12	I understand that in accordance with openness of data findings the anonymised data set from the study may be published in open access and or for wider research. My personal details will not be shared.	☐
13	I agree to take part in the above study.	☐
14	OPTIONAL: I would like to be contacted with end of study information on the trial and my preferred contact method is: email / post / text message *(circle as appropriate).* Contact details will be obtained from medical records.<br>**Circle decision: YES / NO**	☐

**Name of Participant**	
**Signature of Participant**	**Date**
***I certify that the information provided was discussed in a language accessible to the participant. That they retained and understood the information for a sufficient period in order to weigh up their decision and communicate their decision regarding informed consent.**	
***Name of Researcher Obtaining Consent**	
***Signature of Researcher Obtaining Consent**	**Date**

Print double sided. When completed: One for participant, one for Investigator Site File, and one to be kept in medical notes.

2/2
