# Supplementary material for: Use of a targeted, computer/web-based guided self-help psychoeducation toolkit for distressing hallucinations (MUSE) in people with an at-risk mental state for psychosis: protocol for a randomised controlled feasibility trial
Source: BMJ Open. 2023 Jun 30;13(6):e076101. doi: 10.1136/bmjopen-2023-076101 (PMC10314705; doi:10.1136/bmjopen-2023-076101)
Supplement: Supplementary data [file bmjopen-2023-076101supp005.pdf]

[Insert local site logos here]
IRAS Number: Study Title: MUSE ARMS Feasibility Trial.
YP Assent Form [Version 2.0 23022023]
Centre Name: [e.g. CNTW / Other NHS / Participating Organisation]

NHS
Cumbria, Northumberland, Tyne and Wear
NHS Foundation Trust

Participant ID Number:

MUSE ARMS Feasibility Trial

YP ASSENT FORM

		**Initial box to agree**
1	I confirm that I have had time to think about this study. I have had the time to consider the information, ask questions, and have had helpful answers.	☐
2	I understand that taking part is my choice. I am free to stop or take a break at any time without giving any reason.	☐
3	I understand that if I withdraw from the study, or need to be withdrawn due to becoming too unwell, the research team will keep the research data about me that they have already collected, and if you give assent to question 10 on this form they will continue to track long term outcomes via medical records unless you request that they do not.	☐
4	I understand that the research team will only collect information that helps answer the research questions.	☐
5	I understand that my medical notes and the information collected from me will be looked after by the NHS trusts involved in the study for research data quality checks.	☐
6	I agree to my NHS Care Team being told about of my participation in the study.	☐
7	I agree for a short summary of the research assessments and any treatment sessions to be shared with my clinical team (added into my NHS care notes).	☐
8	OPTIONAL: I agree to the audio recording of my treatment sessions. This is to check the treatment is being done properly and not what I am saying.<br>I understand recordings will follow NHS data security standards for storage and will be destroyed once they are checked.<br>**Circle decision: YES / NO**	☐

Print double sided. When completed: One for participant, one for Investigator Site File, and one to be kept in medical notes.

9	OPTIONAL: I agree to take part in an interview about my experience of the study*. These interviews are recorded confidentially and then written out; any identifying information is removed (it's anonymous). *Not everyone is asked to do this. **Circle decision: YES / NO**	☐
10	OPTIONAL : I agree to my medical records being accessed to collect data to look at long term outcomes including use of hospital inpatient services. This requires a copy of my assent form & parent/guardian consent form and my NHS record number to be sent securely to CNTW for processing and storage in the trial master file, which I agree to. **Circle decision: YES / NO**	☐
11	It has been explained that the information collected about me is anonymised (no one will know my name). The information collected is used by researchers for this study who have a job analysing and writing up the findings.	☐
12	It has been explained that the anonymised data set from the study may be published in open access for wider research. My personal details will not be shared (no one will know my name).	☐
13	I would like to take part in the study.	☐
14	OPTIONAL: I would like to be sent end of study information on how it went overall. My preferred contact method is: email / post / text message *(circle preferred).* Contact details will be obtained from medical records. **Circle decision: YES / NO**	☐

**Name of Participant**	
**Signature of Participant**	**Date**
***I certify that the information provided was discussed in a language accessible to the participant. That they retained and understood the information for a sufficient period in order to weigh up their decision and communicate their decision.**	
***Name of Researcher Obtaining Assent**	
***Signature of Researcher Obtaining Assent**	**Date**

Print double sided. When completed: One for participant, one for Investigator Site File, and one to be kept in medical notes.
