# Supplementary material for: Use of a targeted, computer/web-based guided self-help psychoeducation toolkit for distressing hallucinations (MUSE) in people with an at-risk mental state for psychosis: protocol for a randomised controlled feasibility trial
Source: BMJ Open. 2023 Jun 30;13(6):e076101. doi: 10.1136/bmjopen-2023-076101 (PMC10314705; doi:10.1136/bmjopen-2023-076101)
Supplement: Author's manuscript [file bmjopen-2023-076101.draft_revisions.pdf]

BMJ Open

BMJ Open is committed to open peer review. As part of this commitment we make the peer review history of every article we publish publicly available.

When an article is published we post the peer reviewers' comments and the authors' responses online. We also post the versions of the paper that were used during peer review. These are the versions that the peer review comments apply to.

The versions of the paper that follow are the versions that were submitted during the peer review process. They are not the versions of record or the final published versions. They should not be cited or distributed as the published version of this manuscript.

BMJ Open is an open access journal and the full, final, typeset and author-corrected version of record of the manuscript is available on our site with no access controls, subscription charges or pay-per-view fees (http://bmjopen.bmj.com).

If you have any questions on BMJ Open's open peer review process please email info.bmjopen@bmj.com

BMJ Open

A targeted, computer/web based guided self-help psychoeducation toolkit for distressing hallucinations (MUSE) in people with an At Risk Mental State (ARMS) for psychosis: a study protocol for a randomised controlled feasibility trial.

Journal:	*BMJ Open*
Manuscript ID	bmjopen-2023-076101
Article Type:	Protocol
Date Submitted by the Author:	28-May-2023

Complete List of Authors:	Hamilton, Jahnese; Cumbria Northumberland Tyne and Wear NHS Foundation Trust, Research and Development; Newcastle University<br>Arnott, Bronia; Newcastle University, Population Health Sciences Institute<br>Aynsworth, Charlotte; Cumbria Northumberland Tyne and Wear NHS Foundation Trust<br>Barclay, Nicola; Cumbria Northumberland Tyne and Wear NHS Foundation Trust<br>Birkett, Lauren; Cumbria Northumberland Tyne and Wear NHS Foundation Trust<br>Brandon, Toby; Northumbria University<br>Dixon, Lyndsey; Cumbria Northumberland Tyne and Wear NHS Foundation Trust<br>Dudley, Robert; University of York; Cumbria Northumberland Tyne and Wear NHS Foundation Trust<br>Einbeck, J; Durham University<br>Gibbs, Christopher; Cumbria Northumberland Tyne and Wear NHS Foundation Trust<br>Kharatikoopaei, Ehsan; Manchester Metropolitan University; Durham University<br>Simpson, Jennifer; Tees Esk and Wear Valleys NHS Foundation Trust<br>Dodgson, Guy; Cumbria Northumberland Tyne and Wear NHS Foundation Trust<br>Fernyhough, Charles; Durham University

Keywords:	MENTAL HEALTH, PSYCHIATRY, Randomized Controlled Trial

SCHOLARONE™ Manuscripts

A targeted, computer/web based guided self-help psychoeducation toolkit for distressing hallucinations (MUSE) in people with an At Risk Mental State (ARMS) for psychosis: a study protocol for a randomised controlled feasibility trial.

Abstract

Introduction: Individuals who access At Risk Mental State (ARMS) services often have unusual sensory experiences and levels of distress that lead them to seek help. The Managing Unusual Sensory Experiences (MUSE) treatment is a brief symptom targeted intervention that draws on psychological explanations to help account for unusual experiences. Practitioners to use formulation and behavioural experiments to support individuals to make sense of their experiences and enhance coping strategies. The primary objective of this feasibility trial is to resolve key uncertainties before a definitive trial and inform the parameters of a future fully powered trial.

Methods and analysis: 88 participants aged 14-35 accepted into ARMS services, experiencing hallucinations / unusual sensory experiences which are considered by the patient to be a key target problem will be recruited from UK NHS sites and randomised using 1:1 allocation (stratified by site, gender, and age) to either 6-8 sessions of MUSE or time matched treatment as usual. Participants and therapists will be unblind, research assessors are blind. Blinded assessment will occur at baseline, 12 weeks, and 20 weeks post randomisation. Data will be reported in line with CONSORT. Primary trial outcomes are feasibility outcomes, primary participant outcomes are functioning and hallucinations. Additional analysis will investigate potential psychological mechanisms and secondary mental wellbeing outcomes. Trial progression criteria follows signal of efficacy and uses an analytic framework with a traffic-light system to determine viability of a future trial. A subsequent analysis of the NHS England Mental Health Services Data Set 3 years post-randomisation will assess long-term transition to psychosis.

Ethics and dissemination: This trial has received Research Ethics Committee approval. Dissemination will be to ARMS Services, participants, public and patient forums, peer-reviewed publications and conferences.

Trial registration number: ISRCTN58558617.

Funding: National Institute for Health and Care Research (NIHR) Research for Patient Benefit (RfPB) (NIHR204125).

For peer review only

Strengths and limitations of this study

- This is a feasibility RCT. Results will address key uncertainties to inform a future large-scale trial, including sample size and design decisions.
- The MUSE intervention toolkit, the trial design and participant facing materials have been developed with substantial input from people with lived experience.
- This study is distinctive in exploring potential causal cognitive mechanisms in an at risk mental state population who have unusual sensory experiences.
- There is no Gold Standard treatment to compare the intervention to, therefore controlled time-matched Treatment as Usual (TAU) is selected as the comparator. In a meeting with ARMS service leads this was described as supportive psychotherapy.
- The follow-up period is short (20 weeks post randomisation), therefore longer-term participant impacts will not be assessed. However, consent is obtained for a follow-up evaluation at three years to examine transition to psychosis rates from the Mental Health Services Data Set (MHSDS).

Background

At Risk Mental State (ARMS) describes presentations that indicate a potential prodromal stage of psychosis, or risk of psychosis, with around 25% of ARMS individuals converting to psychosis within 36 months (1). The importance of working with these individuals to target possible unhelpful beliefs in development, reduce distress, support healthy functioning, and potentially to prevent the development of full psychosis is widely advocated (2, 3).

The presence of unusual sensory experiences, such as hearing voices and seeing visions (hallucinations), may not in themselves indicate mental ill health as there may be common underlying psychological mechanisms or a continuum of experience from benign, everyday experiences to more severe hallucinations that require treatment (4). However, increased frequency and intensity of hallucinations, alongside distress and a decline in functioning, are linked to transition to psychosis and are threshold criteria in scales recommended in ARMS services (5, 6). Intervening to reduce the distress of unusual sensory experiences and offer explanations of the possible mechanisms behind these experiences may be key in preventing transition to psychosis (3, 7).

Current UK NICE guidelines recommend that people meeting ARMS criteria should be referred for specialist assessment and offered Cognitive Behavioural Therapy (CBT) to reduce the risk of

For peer review only

developing psychosis (8-10). While approaches involving CBT and CBT with supportive therapy show promise in ARMS, the evidence for CBT improving functioning and mental state, or reducing progression to psychosis, is inconclusive (11-13). No specific psychological intervention has been identified as having superior effectiveness in its treatment; there is no 'Gold Standard' treatment (11, 14, 15). ARMS services therefore need to further assess interventions that indicate potential benefit. Robust clinical trials are needed to determine benefits versus risk profiles, accessibility and cost effectiveness (11, 13, 16).

Treatment development would be improved if they addressed key causal mechanisms leading to distressing experiences, and adapted treatment to the needs of different age groups (17, 18). Taking a staged or stepped approach to psychological intervention is good practice, usually with CBT and needs-based interventions prior to pharmacology (8, 18, 19). There is scope for research into briefer approaches implemented prior to CBT in ARMS services, and emerging evidence from early intervention in psychosis research that inclusion of briefer targeted evidence-based interventions prior to CBT may result in a reduction of need for more in-depth CBT, as people better understand their experiences and have less need for interventions (20).

Through extensive multidisciplinary research into voice hearing, clinically embedded research with patients who are indicating at risk state for psychosis, and studies of first episode psychosis, we have developed a targeted, computer/web-based guided self-help psychoeducation toolkit for distressing hallucinations (MUSE) (7, 21). MUSE endeavours to provide scientific and normalising explanations that may provide acceptable and helpful understandings of an individual's unusual sensory experiences and help to prevent more delusional explanations from developing. MUSE has been trialled with an ARMS patient group in a non-randomised study (21) and shown to be acceptable with good participant satisfaction with the therapy. We intend to assess MUSE through a series of trials to determine patient benefit and possible impact on progression to psychosis in patients at high risk. We will also seek to learn more about whether change relates to target mechanisms underlying hallucination subtypes(22). This could be important for further refinement of treatment.

Objectives

Primary objective

To conduct an ISRCTN-registered feasibility randomised controlled trial to resolve key feasibility uncertainties and inform the parameters of a future trial, to investigate the preliminary effect of MUSE+TAU versus time-matched supportive psychotherapy TAU on general functioning (assessed

For peer review only - http://bmjopen.bmj.com/site/about/guidelines.xhtml

using the Social and Occupational Functional Assessment Scale [SOFAS](23), and mental state related to frequency and distress of unusual sensory experiences and false beliefs (assessed using the Psychotic Symptom Rating Scales [PSYRATS](24) total score, and sub-scales Hallucinations and Attribution(25)) in ARMS patients post therapy and at five-month post randomisation follow-up.

Secondary objectives

To explore additional treatment effects on unusual sensory experiences, anxiety, depression, and quality of life, and whether there are indications of other factors (sleep disturbance and trauma) influencing treatment effects.

To test feasibility of collecting measures of psychological mechanisms, including psychological and personal (phenotypical) factors implicated in the clinical course of hallucinations. To analyse which psychological mechanisms are influenced by the treatment and contribute to its clinical effect and inform a future investigation of whether any efficacy of MUSE is through impact on these mechanisms.

To collect routine data for a future records investigation testing feasibility of tracking transition to psychosis through medical databases (hospital records/Mental Health Services Data Set (MHSDS)), to examine which features of MUSE (presenting, treatment response and mechanistic) are most relevant to psychosis prevention.

METHODS AND ANALYSIS

Trial design and flowchart

Methods and Analysis

This is a feasibility trial employing a prospective randomised, open-label, observer blinded, endpoint design assessing a targeted, computer/web based guided self-help psychoeducation toolkit for distressing hallucinations (MUSE)+TAU (6 – 8 sessions) compared to a TAU time matched control (also referred to as supportive psychotherapy) (6 – 8 sessions) offered by a multi-disciplinary team which includes needs based emotional support, psychoeducation and stress management, aiming to reduce distress from hallucinations and improve functioning, in people with an At Risk Mental State (ARMS) for psychosis in UK secondary care mental health services.

For peer review only

The trial has received NHS Research Ethics positive opinion (23/NE/0032) and Health Research Authority Approvals and is registered with the ISRCTN registry (ISRCTN58558617, registered 09.05.2023). Two substantial amendments followed first approval and were obtained prior to first participant consent: Amendment 1 notably added in an unvalidated Preferences Questionnaire for therapeutic intervention, and changed an anxiety self-report questionnaire over to use the State-Trait Anxiety Inventory – Short Form (STAI-Short Form)(26-28). Amendment 2 replaced a longer dissociative experiences questionnaire for the 8-item Brief Dissociative Experiences Scale [DES-B]—Modified(29, 30).

The trial has an independent trial steering committee (TSC) and Lived Experience Advisory Panel (LEAP) facilitated by a co-applicant for the study with lived experience of psychosis.

Participants

Recruitment will be via NHS secondary care mental health clinical teams providing ARMS services. Patients who potentially meet the eligibility criteria for the trial, and their parent/guardian where appropriate if under 18 years, will be informed of the study by a member of their clinical team. Participants will be checked for eligibility prior to informed consent via discussion with referring teams and in the participant-researcher discussion prior to giving informed consent. Participant Information Sheets will be provided at least three days prior to the informed consent meeting. Written informed consent in adherence to principles of Good Clinical Practice (GCP) will be obtained prior to participation. For participants aged 14 and 15 years old, Parent/Guardian informed consent with child assent will be taken; this option of assent with Parent/Guardian consent will also be made available to participants aged 16 and 17 years old due to their potential vulnerability and the governing UK law which classes a minor as someone who is under 18 years old. Verbal consent form will be used for participants with literacy challenges. Interpreters and translated consent forms will be available for participants who do not speak English. Participants will be given £15 honorarium for each assessment time-point.

Trial eligibility criteria

Inclusion criteria

- in contact with an ARMS service or accepted on an ARMS pathway by EIP services
- aged 14–35

For peer review only

- hallucinations / unusual sensory experiences scoring at least 3 on the Perceptual Abnormalities Subscale of the Comprehensive Assessment of At-Risk Mental States (CAARMS)(6)
- hallucinations considered by the patient to be a key target problem
- judged to have been clinically stable for the preceding 2 weeks

Exclusion criteria

- intellectual disability or severe cognitive dysfunction affecting ability to engage with research materials
- lacking capacity to give informed consent

Randomisation and blinding

Eligible participants who have completed baseline assessments will be randomised and subsequent assessments will be scheduled from the point of randomisation. An independent web-based randomisation service (sealedenvelope.com) will be used for the trial. Randomisation will be in the ratio 1:1 to the two groups: MUSE+TAU (intervention) or TAU time matched control (supportive psychotherapy+TAU (control). Randomisation will be stratified by site, gender (M/F/Other) and age (14–17years/18–35 years inclusive). Randomisation allocation will be independent and dynamically generated using a randomised modified minimisation method (31) to assure allocation concealment along with preservation of allocation ratio. Randomisation allocation is made known to the CI and site PIs, the Trial Coordinator(s) and the trial therapists only at the point of randomisation, by email.

Research assessors for the trial will be blind to the allocation throughout the trial. Clinicians, therapists and participants will be unblind. Trial statisticians will be partially blind; In the first instance, for the analyses and reporting of main outcomes of the trial the Statisticians will be fully blind. However, for secondary sensitivity analysis such as impact of number of MUSE sessions on effect size) and the mechanisms investigations, the Statisticians will be required to view which participants received MUSE treatment.

Assessments

Assessors blinded to trial allocation will complete participant assessments at baseline, 12 weeks post randomisation, and 20 weeks post randomisation (See Table 1). Sociodemographic information will be collected from the participant at baseline only (CSRI questions 1-3.5 as amended for the trial(32)).

For peer review only

Table 1: Trial Assessments and Key Participant Procedures Schedule

**Assessments/ procedures**	**Participant identification**	**Enrolment & baseline**	**Randomisation**	**Intervention Weeks 1-12**	**12 weeks post randomisation (+/- 10days)**	**20 weeks post randomisation (+/- 10days)**
Recruitment and eligibility discussions	X					
Informed consent		X				
CSRI Sociodemographic Q1-3.5		X				
Randomisation			X			
MUSE & TAU / TAU Intervention				←→		
**Blinded assessments**						
MUSE ARMS Primary Outcome Measures: SOFAS & PSYRATS		X			X	X
CSRI service use Q4.1-4.4		X				
CSRI Q4.5 criminal justice services and Q5 medication		X			X	X
MUSE ARMS Secondary Outcome Measures: CAARMS-PA, PHQ-9, GAD-7, ReQoL-20, ISI, ITQ/ITQ-CA, MMHQ		X			X	X
Subtype Measures & Cognitive Tasks (1-2 subtypes selected per participant)		X			X	X
Treatment preference		X				
**Unblinded assessments**						
CSRI service use at follow-up Q4.1-4.4					X	X
Transition to Psychosis data					X	X
Adverse Event (AE) data					X	X
Therapeutic Alliance STTS-R					X	
Participants interviews (Withdrawals sub-sample)				←→ (Intervention Weeks 1-12 to 12 weeks)		
Participants interviews (MUSE completers sub-sample)					←→ (12 weeks to 20 weeks)	
Participants interviews (TAU sub-sample)					←→ (12 weeks to 20 weeks)	
Therapists interviews (sub-sample)					←→ (12 weeks to 20 weeks)	

Primary indicators of outcome

The primary outcome measures are: (i) Feasibility outcomes, including qualitative interviews; (ii) General functioning assessed using the SOFAS(23), a clinician/clinical researcher rated single-item scale; (iii) Target problem hallucinations assessed using the PSYRATS(24) (hallucination total) clinician/clinical researcher rated interview, and; (iv) Distress and attribution dimensions of target problem assessed using the PSYRATS (25).

Secondary assessments

For peer review only

Additional assessments will be the clinician/clinical researcher administered semi-structured interview CAARMS subscale of Perceptual Abnormalities(6) to elicit further detail about the nature of unusual experiences. Self-reported measures will rate depression symptom severity (PHQ-9(33)), anxiety (GAD-7(34)), quality of life (ReQoL-20(35)), sleep difficulties (ISI(36)), and trauma (ITQ/ITQ-CA(37, 38)). An unvalidated measure, the Multi-Modal Hallucinations Scale (MMHS) will be used to assess cross-modal sensory experiences.

Service use

Assessment of potential contamination of the MUSE intervention within the TAU condition, of other psychological therapies use within the treatment arms, and the need for additional interventions beyond the treatment phase will be captured using the CSRI(32) (as amended for the trial) at baseline, 12 week and 20 week follow-up. CSRI service use data at 12 weeks and 20 weeks will be collected from medical notes by the unblinded researcher to preserve blinding of research assessors.

Mechanisms assessment

To assess further information on mechanisms, subtype measures & cognitive tasks will be selected per participant for 1 to 2 hallucination subtypes: (i) Inner speech, using the Varieties Of Inner Speech Questionnaire (VISQ-R)(39), and computerised cognitive tasks Auditory Signal Detection Task and Auditory Reality Monitoring Source Memory Task (40, 41); (ii) Memory, using the Brief Dissociative Experiences Scale —Modified (DES-B)(29, 30) and computerised cognitive task Inhibition of Currently Irrelevant Memories (ICIM)(42); (iii) Hypervigilance, using the State-Trait Anxiety Inventory – Short Form (STAI-Short Form)(26-28), and computerised cognitive Jumbled Speech Task (JST)(43, 44), and; (iv) Visual, using the visual section of the Plymouth Sensory Imagery Questionnaire (Psi-Q)(45) , and computerised cognitive tasks Visual Signal Detection(46), Visual Reality Monitoring(47) and Face Pareidolia Task(46). Researchers receive training on subtype selection. Selections are monitored and evaluated against MUSE therapist subtype selections to assess selection reliability and potential training needs.

Acceptability assessment

To assess therapy preference, satisfaction and acceptability of the intervention, participants will be asked about treatment preferences at baseline using a study specific preferences questionnaire (see supplementary materials), and treatment satisfaction post intervention using the Satisfaction with Therapy and Therapist Scale-Revised (STTS-R)(48, 49). Qualitative interviews with participants and trial therapists will further explore experience of MUSE, TAU, and trial procedures.

Long-term outcomes

For peer review only

Long-term transition to psychosis outcomes will be collected 3 years post baseline via the NHS England Mental Health Services Data Set (MHSDS).

Data management

Interview/clinical assessments data will be scored following the visit and entered onto Qualtrics by the researcher. Source data will be retained in the site file. Self-report data will be entered directly onto Qualtrics during visits using participant ID and visit as markers. Unblinded data on service use will be entered onto Qualtrics at the visit time points. Qualtrics outputs and computerised cognitive task data will be downloaded and date stamped at regular intervals to allow data audit. The full data set will be transferred in its anonymous form to the stats team upon completion of data lock at the end of the trial. Trial monitoring at sites will occur across the life cycle of the trial and will follow the Sponsor approved data monitoring plan.

Intervention - Managing Unusual Sensory Experiences (MUSE)+TAU

The MUSE intervention is a novel targeted, computer/web-based guided self-help psychoeducation toolkit and psychological treatment manual for managing distressing hallucinations in mental health, developed and owned jointly by Durham University and CNTW. Patients work with experienced therapists, under expert supervision, who utilise the MUSE package within therapy sessions to develop a formulation explaining the development of hallucinations and foster new skills and strategies for their management. The MUSE treatment is divided into the following modules: What are Voices?; How the Mind Works; Assessment (of participant subtype); Inner Speech; Memory and Trauma; Hypervigilance; and Sleep (see Dudley, Dodgson (50) for details).

Six to eight 1-hour sessions will be offered weekly by experienced therapists who are clinical psychologists or psychological therapists. Therapists will be accredited or working towards accreditation by the British Association of Behavioural and Cognitive Psychotherapists (BABCP), employed by the ARMS service and have experience of MUSE, receiving clinical supervision and fortnightly MUSE supervision. MUSE is loaded onto therapists smart tablet/NHS laptop (not reliant on Wi-Fi) and is available to patients via the CNTW website between sessions. No personal data are recorded or stored on MUSE toolkit.

Session by session measures will be used as part of the MUSE package to enable therapists to monitor any variations in hallucination frequency and distress that may have a bearing on the selection of module used or revisited during the treatment session.

Therapists will be asked to complete adherence checklists for each session contained within a per-participant MUSE Therapist Pack (see supplementary materials). With consent, each session will be

For peer review only

audio-recorded to enable independent review by the site Principal Investigator or delegated Clinical Lead of a random 10% sample to ensure fidelity to protocol within and across sites.

Control condition – Time matched control +TAU

To control for risk of bias from an undefined comparative treatment, and potential bias from dose effects, a time matched TAU is included(11, 51). In order to match the comparative brief intervention to usual practice within ARMS services, components of care were identified in an engagement meeting with ARMS service leads. These common core components could be described as supportive psychotherapy or ongoing care (needs based emotional support, psychoeducation, normalisation and stress management) and were outlined as the interventions used by therapists as part of their normal clinical toolkit, alongside routine multi-disciplinary care from the team. Patients work with different therapists who are ARMS clinicians and are not trained in MUSE. These clinicians will receive supervision on their practice through the routine supervision arrangements of their service and will record the interventions used within a per-participant TAU Therapist Pack (see supplementary materials). We will investigate how frequently and consistently these supportive psychotherapy interventions are offered to inform whether these interventions could act as a comparator intervention in future trials. This arm will be time-matched controlled, however variation across services precluded using this comparator being defined as a controlled *intervention*. Number of sessions received in this group will be recorded for analysis.

Both Groups: Treatment as Usual (TAU)

In addition to the trial allocated intervention (MUSE or TAU Time matched control supportive psychotherapy), both treatment groups will also receive additional usual care as clinically indicated. No treatments will be withheld on account of being part of the trial. This includes regular monitoring, signposting to appropriate local services for unmet needs, social support and crisis management when required from the multi-disciplinary team. CBT is also a core intervention recommended by NICE Guidance and offered across ARMS services. However, in practice it is not always offered to all service users. CBT may form part of the care in both conditions as part of usual care. We will investigate the number of CBT sessions received by participants in both groups and investigate whether MUSE impacts on the number of sessions required. Additional care will be based on clinical judgement and will be recorded for both arms of the study. These additional elements of care, including interventions and contacts that occur beyond the MUSE/time-matched period will be analysed for variations and similarities in the care received between the two groups.

For peer review only

Analysis

Analyses will follow intention to treat principles, with data analysed according to randomisation irrespective of treatment received. A full statistical analysis plan (SAP) will be developed for the outcome measures and agreed with TSC before the end of data collection. Data will be reported in line with the Consolidated Standards of Reporting Trials (CONSORT)(52), see figure 1.

Sample size

As this is a feasibility trial there is no formal sample size calculation, interim analyses or stopping rule. The trial aims to recruit 88 participants with 70 participants completing the study (allowing for 20% attrition) to be able to robustly calculate the sample size for a definitive trial(53).

Statistical analysis

Feasibility outcomes will be analysed primarily: the ability of the trial to recruit participants, who reflect the diversity within the region, and meet study inclusion criteria over the 9-month recruitment period, who complete assessment measures collected at baseline, post intervention and follow-up, until all participants complete the follow-up assessment or withdraw.

Descriptive statistics within each randomised group will be presented for baseline and follow-up points. All data will be summarised as appropriate using mean±standard deviation and median±interquartile range for continuous outcome data; frequency and percentages for binary or categorical data; and rate for count data. Analysis will be via the latest version of R.

The signal of efficacy will be determined by examining the effect of each arm (MUSE versus supportive psychotherapy) on outcomes measures, estimated as change from baseline.

The effects will be estimated using generalised linear mixed effect models with the appropriate distribution and link function. Normal distributions with identity link will be used for continuous outcomes, and negative-binomial distributions with log link for count data outcomes. All binary or categorical outcomes will be analysed using generalised estimating equations (GEE). The mixed-effects models and GEE account for the repeated measurements per participant over the follow-up time points. All models will be adjusted for treatment arms and stratification variables. The mixed model approach taken will allow identifying the individual effect of the two interventions with relation to their baseline, as well as the difference in their effects through an interaction parameter of time and intervention. This can be considered as a model-based difference-in-difference analysis.

For peer review only

These models will be used mainly to estimate relevant parameters, since the trial is not powered for null hypothesis significance-testing. That is, while we are interested in identifying the magnitude of the signal of efficacy, we will not attempt to prove its significance.

In addition, a mediation analysis will examine how the different mechanism components mediate the estimated impact of the interventions on the primary outcomes, and a complier average causal effects (CACE) analysis will determine the impact of the number of sessions on the MUSE effect.

If data are missing for a particular participant and outcome measure, this participant will be excluded from the analysis, for this outcome measure only, without further adjustment for missingness. However, the effect of missing data will be investigated additionally by sensitivity analysis using tabulation of rate of missingness across trial arms and imputation methods.

Qualitative analysis

Audio-recordings will be transcribed and analysed (in NVivo software). Interview transcripts will be analysed using thematic analysis(54) allowing a transparent, replicable and robust process and demonstration of reflexivity and quality. Transcripts will be coded by two researchers until coding reliability is established; coding will then be conducted by one researcher, with reliability checks by the qualitative lead. Data will be extracted into a framework matrix, summarising data by category from individual transcripts, with quotations selected as illustrative exemplars. Initial findings from the qualitative analyses will be presented to LEAP for feedback on interpretation.

Health economics analysis

As a feasibility study, we are not undertaking a formal economic evaluation at this stage but will inform a health economic evaluation in a future definitive trial by piloting the ReQoL-UItility Index with the ReQoL-20 data for health economic analysis calculation.

Criteria for proceeding to a future trial

The signal of efficacy is dependent upon the primary outcome data (SOFAS, PSYRATS Total, PSYRATS distress, PSYRATS attribution) and follows: i) Go: primary outcome data suggest the intervention may show an effect indicating clinical value warranting further investigation; ii) Refine: primary

For peer review only

outcome data indicate no measure of effect, but one or more secondary outcomes indicates an effect; iii) Stop: no effect across any outcomes.

The trial progression criteria will follow signal of efficacy and cover domains of research delivery, therapy engagement and fidelity, and safety. The criteria were influenced by LEAP and TSC input and sign-off. Trial progression criteria uses an analytic framework(58) with a traffic-light system (see Table 2 in supplementary materials). Progression will depend upon: (i) All Green outcomes: no/minor revisions prior to next development of the trial, or; (ii) One or more Amber (but not Red) outcomes: If feasible, substantial alterations to the trial protocol, assessments or intervention, supported by the qualitative work-stream and discussed with TMG and TSC prior to the next development of the trial or; (iii) If one or more Red outcomes result then the trial is unlikely to progress at that site or very substantial amendments are needed. The mechanism measures and tasks will also be reviewed for sensitivity to change and reliability to inform the next development of the trial.

Decisions regarding any changes will consider the ADePT decision-making process to address potential problems with intervention, clinical setting, or trial design that may be relevant in either a trial setting or real world context(58). We will use qualitative data to contextualise our progression criteria, to ensure that the participant feedback informs our understanding of our research delivery and signal of efficacy.

Adverse events

Serious Adverse Events (SAEs) are defined as: results in death; is life-threatening; requires hospitalisation or prolongation of existing hospitalisation; results in persistent or significant disability or incapacity; results in congenital anomaly or birth defect; or is otherwise considered medically significant by the investigator. Any SAEs shall be assessed immediately for trial relatedness and expectedness and reported to the Sponsor. Any related and unexpected SAEs and any Urgent Safety Measures (defined as: early withdrawal of participant(s) due to safety concerns about the intervention or assessments, or; changes to procedures due to concerns about staff or participant safety) shall be reported immediately to the Sponsor and Research Ethics Committee in accordance with Health Research Authority governance regulations (See: https://www.hra.nhs.uk/approvals-amendments/managing-your-approval/safety-reporting/) and Sponsor standard operating procedures.

Adverse events will be recorded for all participants where the event relates to mental state, with focus on clinically significant: a) increases in distress and/or psychosis; b) increased harm to

For peer review only

self/harm to others; c) increased suicidal ideation/attempts; d) increased use of drugs/alcohol; e) emergency room visits for mental health concerns; f) access to crises services.

Patient and public involvement (PPI)

The MUSE intervention, the trial design and participant facing materials and the grant application have benefitted from input by individuals with lived experience. To ensure a retained focus on patients, a Lived Experience Advisory Panel (LEAP) led by a co-applicant with personal lived experience of psychosis was established and meets monthly in a mixture of online and face-to-face formats throughout the lifetime of the trial. The study specific Preferences questionnaire was collaboratively developed with the LEAP. The outcome measures and the topic guides were piloted with LEAP members and amended following feedback. The LEAP were consulted on the potential ethical issues of the trial and the trial progression criteria. Members of the LEAP group will also co-facilitate qualitative interviews, help disseminate study findings, and enable patient experience to inform design of future research and any revisions of the treatment. Two LEAP members are part of the TSC, with one taking a lead on trial procedures and the other on the inclusion of under-served groups. Compensation for work done is given in accordance with NIHR PPI guidelines (https://www.nihr.ac.uk/documents/ppi-patient-and-public-involvement-resources-for-applicants-to-nihr-research-programmes/23437).

Ethics and dissemination

This trial has obtained NHS Research Ethics Committee (REC) positive opinion from Newcastle North Tyneside 1 REC (reference: 23/NE/0032), and UK Health Research Authority approval (IRAS project ID: 323903). The research Sponsor is Cumbria, Northumberland, Tyne and Wear NHS Foundation Trust (CNTW).

An anonymised version of the main outcome quantitative data and mechanisms data will be available either in open access as encouraged by peer review publications or from the trial team on reasonable request with publication of the trial outcomes paper and mechanisms paper.

The research outcomes shall be submitted for peer review open access publications from this trial. Anonymised data will be made available in a repository. Trial outcomes, mechanisms evaluations, and long-term outcomes will be reported on.

For peer review only

The Trial outcomes: The feasibility trial outcomes will report on feasibility outcomes and the candidate primary outcome measures (SOFAS and PSYRATS). Secondary reporting will detail the secondary treatment effects and influence of moderators. Additional reporting will detail treatment integrity: data on treatment adherence to the model (sessions checklist data); exposure of participants to the interventions and additional treatments within usual care (CSRI data); the quality of treatment delivered and responsiveness of participants as reflected on by therapists and participants (STTS-R data, qualitative data); and the programme differentiation between the novel intervention arm and the usual care arm (CSRI data).

Mechanisms will be reported on the analysis of secondary assessments for the purposes of informing which aspects of patient presentation the MUSE intervention works with, and informing the outcome measures in a future efficacy and mechanisms trial.

Long-term transition to psychosis paper: Long-term transition to psychosis through the MHSDS/medical records exploratory feasibility analysis will report which features of MUSE (presentation, treatment response, mechanistic) are indicated as most relevant to psychosis prevention.

Trial status

The trial opened to recruitment at the two planned NHS sites on the 14th April 2023 (Cumbria, Northumberland, Tyne and Wear NHS Foundation Trust) and 21st April 2023 (Tees, Esk and Wear alley NHS Foundation Trust). First participant randomisation (enrolment) was on 10th May 2023. Final participant facing procedures are due to be completed by end of June 2024. The study will finish at NHS research sites after the final assessment with the final participant is completed and the monitoring close-out visit has occurred at site.

Acknowledgments

We are grateful to our Sponsor, Cumbria, Northumberland, Tyne and Wear NHS Foundation Trust and the sponsorship team for their backing and support, and to the many individuals in the collaborating institutions who have enabled this trial to run. We are grateful to the dedicated involvement and contributions of the MARS Lived Experience Advisory Panel Group members, all who wish to remain anonymous and those who are happy to be named. Thank you to Gayl McCain, Katie Rumney, Helen Errington, Patricia Higgins and the whole group. Thanks is noted especially to all of the people who over the course of this research agree to receive information on the study and consider whether would like to take part. A number of people gave their time and expertise in reviewing iterations of the trial development and supporting the trial through the various funding

For peer review only

and approvals systems, who include Stephanie Common, Angus Williams, and others, and our appreciation is noted. The study has also benefitted from the technical contributions of the psychologists David Smailes (ORCID ID: 0000-0002-0455-070X) and Peter Moseley (ORCID ID: 0000-0002-9284-2509) who respectively built the computerised cognitive tasks used in the trial, we are grateful for this contribution. We would not be able to run the trial without the daily contribution also from all of the trial researchers on the ground who work within the NHS trusts to support the day to day running of trial operations, communication with participants, recruitment to the research and to conduct research assessments, thank you to Michael Kelly, Abbey Underwood, Alexandra Carne, Sarah Bentley, Emily Clare, Melanie Rowe, Victoria Wilson, Sarah Garner and others, thank you. Tremendous thanks is also given to all of the therapists who work into the NHS ARMS services who are delivering the interventions for this study, Carina Ewels, Eloise Todd, Louise Ross, Andrew Weightman, Claire Mason, Toyah Lebert, Mark Dawson, Louise Leach, Nicola Elliot, Paul Williams, Luke Rogers, Alison Bernard and to all of the individuals who support these services to function.

Contributors

Authorship follows ICMJE recommendations: https://www.icmje.org/recommendations/browse/roles-and-responsibilities/defining-the-role-of-authors-and-contributors.html JH is the trial manager and wrote the final protocol and protocol paper. GD is the chief investigator and has overall responsibility for the trial design and drafted the initial trial protocol. GD and CF are the joint study leads and lead on supervision and trial decisions. BA, CA, NB, TB, LD, CG, and JS contributed to the study design. GD and CF led the development of the treatment with substantial input from a range of clinicians and service users. CG was responsible for patient and public involvement. JE and EK are responsible for the statistical analysis design. NB, JS, and LB coordinated the trial at sites and were responsible for recruitment. All authors read and approved the final trial protocol.

Funding

This project is funded by the National Institute for Health and Care Research (NIHR) under its Research for Patient Benefit (RfPB) Programme (Grant Reference Number NIHR204125). The views expressed are those of the author(s) and not necessarily those of the NIHR or the Department of Health and Social Care.

Competing interests

GD, RD, JS, NB and CA provide psychological therapies for individuals with psychosis in NHS settings. RD receives payment for workshops in treating hallucinations. GD, RD, CF, NB, and CA hold or have

For peer review only

held grants to develop and carry out trials of psychological therapy for individuals with psychosis. LD provides sponsorship oversight of trials in CNTW NHS FT. All other authors declare no competing interest. The MUSE toolkit is the joint intellectual property of Durham University and Cumbria, Northumberland, Tyne and Wear NHS Foundation Trust and will be made freely available if proven to be beneficial.

Patient and public involvement

Patients and/or the public were involved in the design, or conduct, or reporting, or dissemination plans of this research. Refer to the Methods section for further details.

Patient consent for publication

Not applicable.

Provenance and peer review

Not commissioned; peer reviewed for ethical and funding approval prior to submission.

Open access

This is an open access article distributed in accordance with the Creative Commons Attribution 4.0 Unported (CC BY 4.0) license, which permits others to copy, redistribute, remix, transform and build upon this work for any purpose, provided the original work is properly cited, a link to the licence is given, and indication of whether changes were made. See: https://creativecommons.org/licenses/by/4.0/.

For peer review only

References

1. Salazar de Pablo G, Radua J, Pereira J, Bonoldi I, Arienti V, Besana F, et al. Probability of Transition to Psychosis in Individuals at Clinical High Risk An Updated Meta-analysis. JAMA Psychiatry. 2021;78:970-8.
2. Salazar De Pablo G, Estradé A, Cutroni M, Andlauer O, Fusar-Poli P. Establishing a clinical service to prevent psychosis: What, how and when? Systematic review. Translational Psychiatry. 2021;11(1).
3. Yung AR, Wood SJ, Malla A, Nelson B, McGorry P, Shah J. The reality of at risk mental state services: a response to recent criticisms. Psychological Medicine. 2021;51(2):212-8.
4. Toh WL, Moseley P, Fernyhough C. Hearing voices as a feature of typical and psychopathological experience. Nature Reviews Psychology. 2022;1(2):72-86.
5. Nelson B, Yung A, Markulev C, Nicoll M. The CAARMS: assessing young people at ultra high risk of psychosis: Orygen Youth Health Research Centre; 2014.

6. Yung AR, Yuen HP, McGorry PD, Phillips LJ, Kelly D, Dell'Olio M, et al. Mapping the onset of psychosis: the Comprehensive Assessment of At-Risk Mental States. Aust N Z J Psychiatry 2005;39:964–71.
7. Dodgson G, Alderson-Day B, Smailes D, Ryles F, Mayer C, Glen-Davison J, et al. Tailoring cognitive behavioural therapy to subtypes of voice-hearing using a novel tabletised manual: a feasibility study. Behavioural and Cognitive Psychotherapy. 2021;49(3):287-301.
8. NICE. Psychosis and schizophrenia in adults: prevention and management. 2014:1-40.
9. NICE. Psychosis and schizophrenia. 2021.
10. NICE. Psychosis and schizophrenia in children and young people: recognition and management. 2013:1-48.
11. Bosnjak Kuharic D, Kekin I, Hew J, Rojnic Kuzman M, Puljak L. Interventions for prodromal stage of psychosis. Cochrane Database Syst Rev. 2019;2019(11).
12. Mei C, van der Gaag M, Nelson B, Smit F, Yuen HP, Berger M, et al. Preventive interventions for individuals at ultra high risk for psychosis: An updated and extended meta-analysis. Clinical psychology review. 2021;86:102005.
13. Stafford MR, Jackson H, Mayo-Wilson E, Morrison AP, Kendall T. Early interventions to prevent psychosis: systematic review and meta-analysis. BMJ. 2013;346(jan18 1):f185-f.
14. Fusar-Poli P, Davies C, Solmi M, Brondino N, De Micheli A, Kotlicka-Antczak M, et al. Preventive Treatments for Psychosis: Umbrella Review (Just the Evidence). Front Psychiatry. 2019;10:764.
15. van der Gaag M, van den Berg D, Ising H. CBT in the prevention of psychosis and other severe mental disorders in patients with an at risk mental state: A review and proposed next steps. Schizophrenia research. 2019;203:88-93.
16. Aceituno D, Vera N, Prina AM, McCrone P. Cost-effectiveness of early intervention in psychosis: systematic review. The British Journal of Psychiatry. 2019;215(01):388-94.
17. Wykes T, Bell A, Carr S, Coldham T, Gilbody S, Hotopf M, et al. Shared goals for mental health research: what, why and when for the 2020s. Journal of Mental Health. 2021:1-9.
18. Schmidt SJ, Schultze-Lutter F, Schimmelmann BG, Maric NP, Salokangas RK, Riecher-Rössler A, et al. EPA guidance on the early intervention in clinical high risk states of psychoses. Eur Psychiatry. 2015;30(3):388-404.
19. Addington J, Addington D, Abidi S, Raedler T, Remington G. Canadian Treatment Guidelines for Individuals at Clinical High Risk of Psychosis. Can J Psychiatry. 2017;62(9):656-61.
20. Drake RJ, Day CJ, Picucci R, Warburton J, Larkin W, Husain N, et al. A naturalistic, randomized, controlled trial combining cognitive remediation with cognitive-behavioural therapy after first-episode non-affective psychosis. Psychol Med. 2014;44(9):1889-99.
21. Dodgson G, Aynsworth C, Mitrenga KJ, Gibbs C, Patton V, Fernyhough C, et al. Managing unusual sensory experiences: A feasibility trial in an At Risk Mental States for psychosis group. Psychology and Psychotherapy: Theory, Research and Practice. 2021;94(3):481-503.
22. McCarthy-Jones S, Thomas N, Strauss C, Dodgson G, Jones N, Woods A, et al. Better than mermaids and stray dogs? Subtyping auditory verbal hallucinations and its implications for research and practice. Schizophrenia bulletin. 2014;40 Suppl 4(Suppl 4):S275-84.
23. Goldman HH, Skodol AE, Lave TR. Revising axis V for DSM-IV: a review of measures of social functioning. Am J Psychiatry. 1992;149(9):1148-56.
24. Haddock G, McCarron J, Tarrier N, Faragher EB. Scales to measure dimensions of hallucinations and delusions: the psychotic symptom rating scales (PSYRATS). Psychol Med. 1999;29(4):879-89.
25. Woodward TS, Jung K, Hwang H, Yin J, Taylor L, Menon M, et al. Symptom dimensions of the psychotic symptom rating scales in psychosis: a multisite study. Schizophrenia bulletin. 2014;40 Suppl 4(Suppl 4):S265-74.
26. Zsido AN, Teleki SA, Csokasi K, Rozsa S, Bandi SA. Development of the short version of the spielberger state-trait anxiety inventory. Psychiatry Res. 2020;291:113223.

For peer review only

27. Spielberger CD. State-Trait Anxiety Inventory. The Corsini Encyclopedia of Psychology1983.
28. Spielberger CD, Gorsuch RL, Lushene RE. Manual for the State-Trait Anxiety Inventory1970.
29. Dalenberg C, Carlson E. Severity of Dissociative Symptoms—Adult (Brief Dissociative Experiences Scale [DES-B]—Modified) [Measurement instrument]. American Psychiatric Association; 2010.
30. Dalenberg C, Carlson E. Severity of Dissociative Symptoms—Child Age 11–17 (Brief Dissociative Experiences Scale [DES-B]—Modified) [Measurement instrument]. . American Psychiatric Association; 2010.
31. Kuznetsova OM, Tymofyeyev Y. Preserving the allocation ratio at every allocation with biased coin randomization and minimization in studies with unequal allocation. Statistics in Medicine. 2012;31(8):701-23.
32. Beecham J, Knapp M. Costing psychiatric interventions. In: Thornicroft G, editor. Measuring Mental Health Needs. 2nd ed: Gaskell; 2001. p. 200-24.
33. Kroenke K, Spitzer RL, Williams JB. The PHQ-9: validity of a brief depression severity measure. J Gen Intern Med. 2001;16(9):606-13.
34. Spitzer RL, Kroenke K, Williams JB, Löwe B. A brief measure for assessing generalized anxiety disorder: the GAD-7. Arch Intern Med. 2006;166(10):1092-7.
35. Keetharuth AD, Brazier J, Connell J, Bjorner JB, Carlton J, Taylor Buck E, et al. Recovering Quality of Life (ReQoL): a new generic self-reported outcome measure for use with people experiencing mental health difficulties. Br J Psychiatry. 2018;212(1):42-9.
36. Bastien CH, Vallières A, Morin CM. Validation of the Insomnia Severity Index as an outcome measure for insomnia research. Sleep Med. 2001;2(4):297-307.
37. Cloitre M, Shevlin M, Brewin CR, Bisson JI, Roberts NP, Maercker A, et al. The International Trauma Questionnaire: development of a self-report measure of ICD-11 PTSD and complex PTSD. Acta Psychiatr Scand. 2018;138(6):536-46.
38. Haselgruber A, Sölva K, Lueger-Schuster B. Symptom structure of ICD-11 Complex Posttraumatic Stress Disorder (CPTSD) in trauma-exposed foster children: examining the International Trauma Questionnaire - Child and Adolescent Version (ITQ-CA). Eur J Psychotraumatol. 2020;11(1):1818974.
39. Alderson-Day B, Mitrenga K, Wilkinson S, McCarthy-Jones S, Fernyhough C. The varieties of inner speech questionnaire – Revised (VISQ-R): Replicating and refining links between inner speech and psychopathology. Consciousness and Cognition. 2018;65:48-58.
40. Bentall RP, Slade PD. Reality testing and auditory hallucinations: a signal detection analysis. Br J Clin Psychol. 1985;24 (Pt 3):159-69.
41. Moseley P, Aleman A, Allen P, Bell V, Bless J, Bortolon C, et al. Correlates of Hallucinatory Experiences in the General Population: An International Multisite Replication Study. Psychological Science. 2021;32(7):1024-37.
42. Paulik G, Badcock JC, Maybery MT. Poor intentional inhibition in individuals predisposed to hallucinations. Cognitive Neuropsychiatry. 2007;12:457-70.
43. Campbell ML, Morrison AP. The relationship between bullying, psychotic-like experiences and appraisals in 14-16-year olds. Behav Res Ther. 2007;45(7):1579-91.
44. Fernyhough C, Bland K, Meins E, Coltheart M. Imaginary companions and young children's responses to ambiguous auditory stimuli: implications for typical and atypical development. J Child Psychol Psychiatry. 2007;48(11):1094-101.
45. Andrade J, May J, Deeprose C, Baugh SJ, Ganis G. Assessing vividness of mental imagery: The Plymouth Sensory Imagery Questionnaire. Br J Psychol. 2014;105(4):547-63.
46. Smailes D, Burdis E, Gregoriou C, Fenton B, Dudley R. Pareidolia-proneness, reality discrimination errors, and visual hallucination-like experiences in a non-clinical sample. Cognitive Neuropsychiatry. 2020;25:113-25.

For peer review only

47. Aynsworth C, Nemat N, Collerton D, Smailes D, Dudley R. Reality monitoring performance and the role of visual imagery in visual hallucinations. Behaviour Research and Therapy. 2017;97:115-22.
48. Oei TP, Shuttlewood GJ. Development of a Satisfaction with Therapy and Therapist Scale. Aust N Z J Psychiatry. 1999;33(5):748-53.
49. Oei TPS, Green AL. The Satisfaction With Therapy and Therapist Scale--Revised (STTS-R) for group psychotherapy: Psychometric properties and confirmatory factor analysis. Professional Psychology: Research and Practice. 2008;39(4):435-42.
50. Dudley R, Dodgson G, Common S, O'Grady L, Watson F, Gibbs C, et al. Managing Unusual Sensory Experiences in People with First-Episode Psychosis (MUSE FEP): a study protocol for a single-blind parallel-group randomised controlled feasibility trial. BMJ Open. 2022;12(5):e061827.
51. Higgins JPT, Green S. Cochrane handbook for systematic reviews of interventions: John Wiley & Sons; 2011.
52. Schulz KF, Altman DG, Moher D, the CG. CONSORT 2010 Statement: updated guidelines for reporting parallel group randomised trials. BMC Medicine. 2010;8(1):18.
53. Teare MD, Dimairo M, Shephard N, Hayman A, Whitehead A, Walters SJ. Sample size requirements to estimate key design parameters from external pilot randomised controlled trials: a simulation study. Trials. 2014;15(1):264.
54. Braun V, Clarke V. Using thematic analysis in psychology. Qualitative research in psychology. 2006;3:77-101.

For peer review only

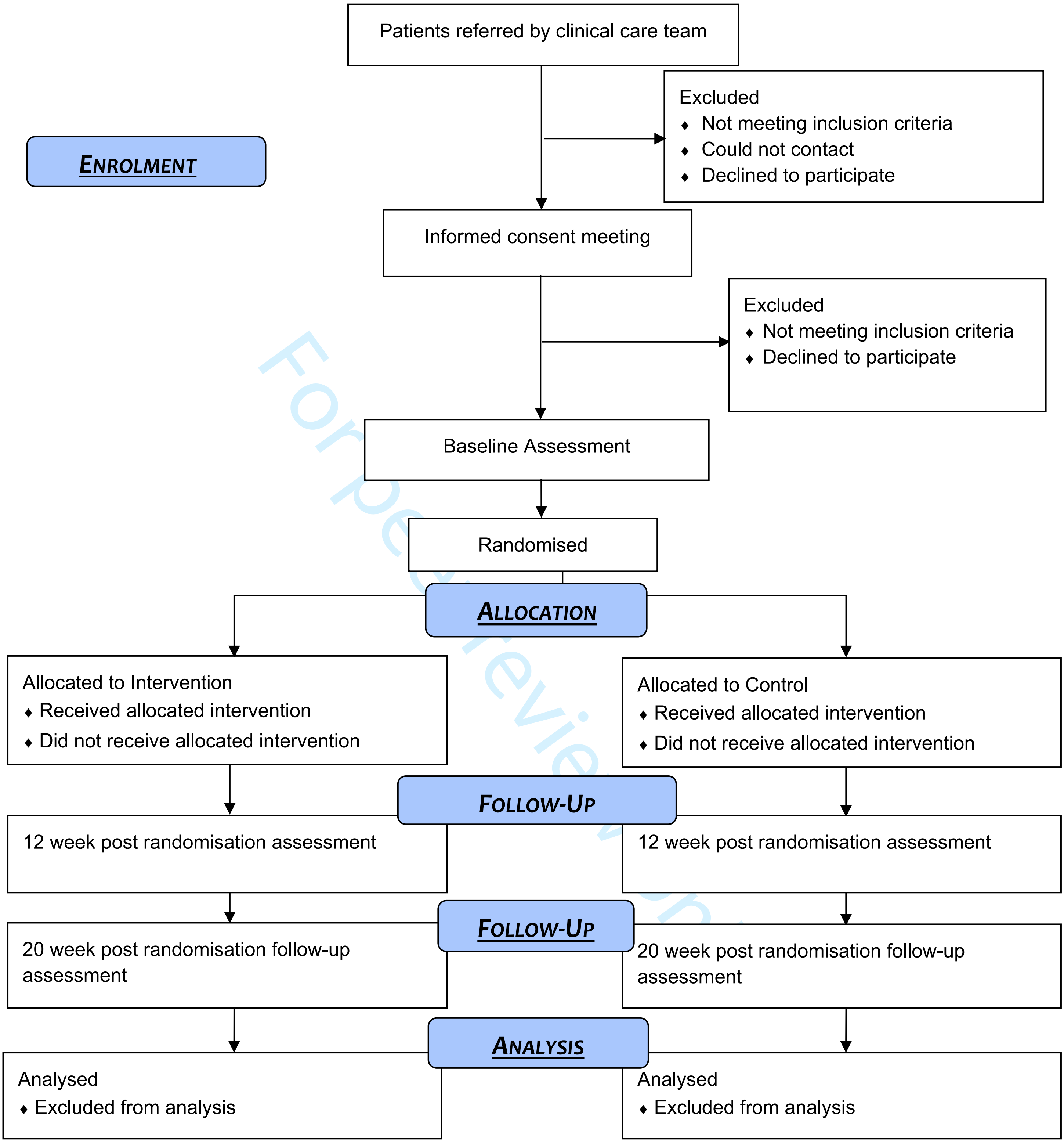
Patients referred by clinical care team
Excluded
Not meeting inclusion criteria
Could not contact
Declined to participate
ENROLMENT
Informed consent meeting
Excluded
Not meeting inclusion criteria
Declined to participate
Baseline Assessment
Randomised
ALLOCATION
Allocated to Intervention
Received allocated intervention
Did not receive allocated intervention
Allocated to Control
Received allocated intervention
Did not receive allocated intervention
FOLLOW-UP
12 week post randomisation assessment
12 week post randomisation assessment
FOLLOW-UP
20 week post randomisation follow-up assessment
20 week post randomisation follow-up assessment
ANALYSIS
Analysed
Excluded from analysis
Analysed
Excluded from analysis

Table 2. Approved trial monitoring and progression criteria to a future definitive trial

Criterion	Critical feasibility outcome	Other feasibility and acceptability data relevant to the criterion	Proposed thresholds on critical outcome
**1) Recruitment**	• Number of participants consented into the trial and randomised	• Number of referrals per month<br>• Source of recruitment<br>• Number of participants eligible,<br>• Number of participants referred<br>• Reasons for non-eligibility or withdrawal of interest	Feasibility will be demonstrated where an average of least 7.84 participants are recruited and randomised per month (80% of recruitment target met).<br>If at least 5.88 participants are recruited per month, then a future trial will be feasible but additional strategies must be identified to support recruitment (e.g. informed by other feasibility data relevant to this criterion) (60-80% of recruitment target met).<br>If an average of under 5.88 participant is recruited per month over the recruitment period, feasibility within the current design will not be demonstrated (under 60% of recruitment target met).
**2) Therapy engagement**	• % who drop-out of therapy	• Session record forms for each therapy session<br>• Number of therapy sessions attended<br>• Qualitative interviews with SU participants<br>• Therapy satisfaction scores	Feasibility will be demonstrated if at least 80% of the participants in the intervention arm completed at least 4 out of the 6-8 sessions of MUSE.<br>If 60-80% of participants in the intervention arm complete at least 4 out of the 6-8 sessions of MUSE.<br>If less than 60% of participants in the intervention arm complete at least 4 out of the 6-8 sessions of MUSE.
**3) Assessment retention**	• % of participants who are lost to follow-up at primary assessment endpoint (12weeks post randomisation)	• Reasons for withdrawal from the study<br>• Qualitative interviews with SU participants<br>• Data completion	If at least 70% of participants complete primary outcome measure at primary assessment endpoint, feasibility will be demonstrated.<br>If 50-70% of participants complete primary outcome measure at primary assessment endpoint, a future trial will be feasible if strategies to overcome barriers are identified (e.g. via other data relevant to this).<br>If less than 50% of participants complete primary outcome measure at primary assessment endpoint, feasibility within the current design will not be demonstrated.
**4) Therapy fidelity**	• Adherence ratings from therapy tapes	• Session record form for each therapy session (including reasons for deviation from protocol)	Feasibility will be demonstrated if over 80% of rated therapy tapes are rated as acceptable.<br>If 50-80% of rated therapy tapes are rated as acceptable, a future trial will be feasible if strategies to overcome barriers are identified<br>If less than 50% of rated therapy tapes will be rates as acceptable, feasibility within the current design will not be demonstrated.
**5) Safety**	• Number of related SAEs	• Increased number of AEs in Intervention condition	0-1 Related SAEs in the Intervention arm.<br>2 Related SAEs in the Intervention arm.<br>3+ Related SAEs in the Intervention arm.

For peer review only

For peer review only

MUSE ARMS Feasibility Trial: Preferences Questionnaire; v1.0, 12.03.2023
IRAS Project ID: 323903

Preferences for psychological therapy or support

This set of questions is seeking to develop an understanding of your preferences for therapy treatment.

1. Do you have a preference for the number of therapy sessions you might get?

◯ 1-3

◯ 4-8

◯ 9-16

◯ 17-30

◯ Don't know

2. How important is it that your treatment:

Includes being give medication?

◯ Not important

◯ Somewhat important

◯ Very important

Includes a talking therapy?

◯ Not important

◯ Somewhat important

◯ Very important

Addresses any feelings of anxiety?

◯ Not important

◯ Somewhat important

◯ Very important

For peer review only - http://bmjopen.bmj.com/site/about/guidelines.xhtml

For peer review only

MUSE ARMS Feasibility Trial: Preferences Questionnaire; v1.0, 12.03.2023
IRAS Project ID: 323903

Addresses any feeling of low mood?

- ◯ Not important
- ◯ Somewhat important
- ◯ Very important

Helps you understand the causes of any unusual sensory experiences, such as hearing a voice?

- ◯ Not important
- ◯ Somewhat important
- ◯ Very important

Helps you learn to manage any unusual sensory experiences?

- ◯ Not important
- ◯ Somewhat important
- ◯ Very important

Helps you feel less distressed about any unusual sensory experiences?

- ◯ Not important
- ◯ Somewhat important
- ◯ Very important

3. What are your preferences for the way the therapist/clinical care team works with you? Please rate how important you think the following statements are:

I am given space to talk and feel heard

- ◯ Not important
- ◯ Somewhat important
- ◯ Very important

For peer review only - http://bmjopen.bmj.com/site/about/guidelines.xhtml

MUSE ARMS Feasibility Trial: Preferences Questionnaire; v1.0, 12.03.2023
IRAS Project ID: 323903

I work with my therapist to help me make sense of my experiences

◯ Not important

◯ Somewhat important

◯ Very important

I am involved in setting my own goals

◯ Not important

◯ Somewhat important

◯ Very important

I am given new ideas of how to cope with my experiences

◯ Not important

◯ Somewhat important

◯ Very important

4. How much do you hope to get the MUSE therapy?

◯ I would prefer to be allocated to MUSE based therapy

◯ I don't mind one way or the other whether I receive MUSE based therapy

◯ I would prefer to be allocated to the treatment as usual.

For peer review only

For peer review only - http://bmjopen.bmj.com/site/about/guidelines.xhtml

MUSE THERAPY THERAPIST PACK

*Return to research site file for archiving after completion

For peer review only - http://bmjopen.bmj.com/site/about/guidelines.xhtml

Adherence Checklist: MUSE Therapy Sessions
(Please tick topic used in any session)

**Insert Date**:									
**Insert length of session (minutes):**									
**Module/Topic**	**S1**	**S2**	**S3**	**S4**	**S5**	**S6**	**S7**	**S8**	**Comments**
**What are voices?**									
What are voices?									
How many people hear voices?									
Why does it become a problem?									
Can things get better?									
Personal experiences									
1. **How the mind works?**									
Thoughts and senses									
How thoughts work									
Embarrassing thoughts									
The power of attention									
How we use expectation									
2. **Assessment**									
Types of unusual sensory experiences.									
What kind of voices do we hear?									
3. **Inner Speech**									
What is inner speech?									
Our inner speech can do amazing things									
Why do people not recognise voices?									
Thoughts are hard to control									
Blocking the loop									
Inner speech – what is the evidence?									
Tracking the self – Was that me?									
Writers and voice hearing									
Imaginary friends									
Formulation									
Voices and Relationships									
Transforming the voice									
Testing out your explanations									

Completed by

Participant ID (MUSE ID Number):	Print Name:	Role:	Signature:	**Completed on (DD/MM/YYYY)**

For peer review only

Living well with voices									
4. **Memory Based Voices**									
Memory, dissociation, trauma									
The importance of trauma									
Threat system and Soothing system									
Formulation									
Treating trauma									
5. **Hypervigilance**									
Nature versus Nurture									
Filling in the gaps									
What our perception system is designed to do									
Response to danger									
Formulation									
Threat system and soothing system									
Mistrust									
6. **Seeing Visions**									
Is seeing believing?									
What do your visions mean to you?									
Perception system design									
Filling in the gaps									
Tracking the self – was that me?									
Imaginary friends									
Testing distressing appraisals									
Changing images									
Living well with visual experiences									
Voices, visions and relationships									
Challenging unacceptability									
Testing out your explanations									
Living well with voices and visions									
7. **Sleep**									
Why do we sleep?									
How to sleep well									

	**Completed by**			
Participant ID (MUSE ID Number):	Print Name:	Role:	Signature:	**Completed on (DD/MM/YYYY)**

For peer review only

Adverse Events Checklist

Insert date:									
	**S1**	**S2**	**S3**	**S4**	**S5**	**S6**	**S7**	**S8**	**Comments**
**Adverse events of interest reported?** **Add to Rio/Paris**									
**Serious Adverse Event? NB 24hour reporting deadline**									
**Urgent Safety Measures? NB Phone PI immediately**									

Adverse Events Guidance:

Adverse Events. Record on Rio/Paris for collection by the Unblinded Researcher at the 12wk and 20wk assessment time points that pertain to the following events of Protocol Interest:

- Clinically significant increases in distress and/or psychosis
- Increased harm to self/harm to others
- Increased suicidal ideation/attempts
- Increased use of drugs/alcohol
- Emergency room visits for mental health concerns
- Access to crises services

Serious Adverse Event (SAE): The site Principal Investigator (PI), or delegate shall report all SAEs within 24 hours of becoming aware of the event to the Chief Investigator (CI), or delegate via email to MUSE.ARMS@cntw.nhs.uk using the SAE reporting form. These are events that:

- results in death;
- is life-threatening;
- requires hospitalisation or prolongation of existing hospitalisation;
- results in persistent or significant disability or incapacity;
- consists of a congenital anomaly or birth defect; or,
- is otherwise considered medically significant by the investigator.

Urgent Safety Measures: The site Principal Investigator (PI), or delegate, must inform the CI immediately by telephone (Tel. 01670844670 / alternatively Teams video/voice call for guy.dodgson@cntw.nhs.uk) of urgent safety measures defined above in section 11.5 (early withdrawal/changes to procedure due to safety concerns for staff or participants). This information shall be documented on the Urgent safety reporting form and submitted by email to MUSE.ARMS@cntw.nhs.uk. This is when the following applies:

- Early withdrawal of participant(s) due to safety concerns about the intervention or assessments
- Changes to procedures due to concerns about staff or participant safety

Transition to Psychosis Checklist

Insert date:									
	**S1**	**S2**	**S3**	**S4**	**S5**	**S6**	**S7**	**S8**	**Comments**
**Indication of Transition to Psychosis?** **Add note to Rio/Paris**									

Transition to Psychosis Guidance: The following information suggests potential transition to psychosis for this protocol:

- Clinical diagnosis using standard diagnostic classification systems DSM/ICD
- Clinical diagnosis using ARMS assessment schedule documented in clinical notes
- Transfer to the Early Intervention in Psychosis pathway
- Treated or untreated psychotic episode of one week's duration or longer
- Initiation of treatment with antipsychotics (3 or more weeks of treatment with antipsychotics at a dose of ≥ 5mg haloperidol or equivalent)

Completed by

Participant ID (MUSE ID Number):	Print Name:	Role:	Signature:	**Completed on (DD/MM/YYYY)**

For peer review only

Therapy Session measure

MUSE Therapist Pack MUSE ARMS Feasibility Trial; IRAS ID: 323903

Session Number:

Date:

Therapy Session measure

Please answer the following questions about the voices you experienced <u>in the past week</u>

<u>How frequent were the voices?</u>

0%	10	20	30	40	50	60	70	80	90	100%
Voices not present					Once a day					Voices always present

<u>Were the voices distressing? How much of the time?</u>

0%	10	20	30	40	50	60	70	80	90	100%
Voices never distressing					Voices were distressing about half of the times					Voices always distressing

If relevant please answer the following questions about the visions you experienced <u>in the past week</u>

<u>How frequent were the visions?</u>

0%	10	20	30	40	50	60	70	80	90	100%
Visions not present					Once a day					Visions always present

<u>Were the vision distressing? How much of the time?</u>

0%	10	20	30	40	50	60	70	80	90	100%
Visions never distressing					Moderately distressing					extremely distressing

<u>Overall, how distressing were the experiences listed above?</u>

0%	10	20	30	40	50	60	70	80	90	100%
not at all distressing					Moderately distressing					extremely distressing

	Completed by			
Participant ID (MUSE ID Number):	Print Name:	Role:	Signature:	**Completed on (DD/MM/YYYY)**

For peer review only

Therapy Session measure

MUSE Therapist Pack MUSE ARMS Feasibility Trial; IRAS ID: 323903

Session Number:

Date:

Therapy Session measure

Please answer the following questions about the voices you experienced <u>in the past week</u>

<u>How frequent were the voices?</u>

0%	10	20	30	40	50	60	70	80	90	100%
Voices not present					Once a day					Voices always present

<u>Were the voices distressing? How much of the time?</u>

0%	10	20	30	40	50	60	70	80	90	100%
Voices never distressing					Voices were distressing about half of the times					Voices always distressing

If relevant please answer the following questions about the visions you experienced <u>in the past week</u>

<u>How frequent were the visions?</u>

0%	10	20	30	40	50	60	70	80	90	100%
Visions not present					Once a day					Visions always present

<u>Were the vision distressing? How much of the time?</u>

0%	10	20	30	40	50	60	70	80	90	100%
Visions never distressing					Moderately distressing					extremely distressing

<u>Overall, how distressing were the experiences listed above?</u>

0%	10	20	30	40	50	60	70	80	90	100%
not at all distressing					Moderately distressing					extremely distressing

Completed by

Participant ID (MUSE ID Number):	Print Name:	Role:	Signature:	**Completed on (DD/MM/YYYY)**

For peer review only

Therapy Session measure

MUSE Therapist Pack MUSE ARMS Feasibility Trial; IRAS ID: 323903

Session Number:

Date:

Therapy Session measure

Please answer the following questions about the voices you experienced <u>in the past week</u>

<u>How frequent were the voices?</u>

0%	10	20	30	40	50	60	70	80	90	100%
Voices not present					Once a day					Voices always present

<u>Were the voices distressing? How much of the time?</u>

0%	10	20	30	40	50	60	70	80	90	100%
Voices never distressing					Voices were distressing about half of the times					Voices always distressing

If relevant please answer the following questions about the visions you experienced <u>in the past week</u>

<u>How frequent were the visions?</u>

0%	10	20	30	40	50	60	70	80	90	100%
Visions not present					Once a day					Visions always present

<u>Were the vision distressing? How much of the time?</u>

0%	10	20	30	40	50	60	70	80	90	100%
Visions never distressing					Moderately distressing					extremely distressing

<u>Overall, how distressing were the experiences listed above?</u>

0%	10	20	30	40	50	60	70	80	90	100%
not at all distressing					Moderately distressing					extremely distressing

	Completed by			
Participant ID (MUSE ID Number):	Print Name:	Role:	Signature:	**Completed on (DD/MM/YYYY)**

For peer review only

Therapy Session measure

MUSE Therapist Pack MUSE ARMS Feasibility Trial; IRAS ID: 323903

Session Number:

Date:

Therapy Session measure

Please answer the following questions about the voices you experienced <u>in the past week</u>

<u>How frequent were the voices?</u>

0%	10	20	30	40	50	60	70	80	90	100%
Voices not present					Once a day					Voices always present

<u>Were the voices distressing? How much of the time?</u>

0%	10	20	30	40	50	60	70	80	90	100%
Voices never distressing					Voices were distressing about half of the times					Voices always distressing

If relevant please answer the following questions about the visions you experienced <u>in the past week</u>

<u>How frequent were the visions?</u>

0%	10	20	30	40	50	60	70	80	90	100%
Visions not present					Once a day					Visions always present

<u>Were the vision distressing? How much of the time?</u>

0%	10	20	30	40	50	60	70	80	90	100%
Visions never distressing					Moderately distressing					extremely distressing

<u>Overall, how distressing were the experiences listed above?</u>

0%	10	20	30	40	50	60	70	80	90	100%
not at all distressing					Moderately distressing					extremely distressing

Completed by

Participant ID (MUSE ID Number):	Print Name:	Role:	Signature:	**Completed on (DD/MM/YYYY)**

For peer review only

Therapy Session measure

MUSE Therapist Pack MUSE ARMS Feasibility Trial; IRAS ID: 323903

Session Number:

Date:

Therapy Session measure

Please answer the following questions about the voices you experienced <u>in the past week</u>

<u>How frequent were the voices?</u>

0%	10	20	30	40	50	60	70	80	90	100%
Voices not present					Once a day					Voices always present

<u>Were the voices distressing? How much of the time?</u>

0%	10	20	30	40	50	60	70	80	90	100%
Voices never distressing					Voices were distressing about half of the times					Voices always distressing

If relevant please answer the following questions about the visions you experienced <u>in the past week</u>

<u>How frequent were the visions?</u>

0%	10	20	30	40	50	60	70	80	90	100%
Visions not present					Once a day					Visions always present

<u>Were the vision distressing? How much of the time?</u>

0%	10	20	30	40	50	60	70	80	90	100%
Visions never distressing					Moderately distressing					extremely distressing

<u>Overall, how distressing were the experiences listed above?</u>

0%	10	20	30	40	50	60	70	80	90	100%
not at all distressing					Moderately distressing					extremely distressing

	Completed by			
Participant ID (MUSE ID Number):	Print Name:	Role:	Signature:	**Completed on (DD/MM/YYYY)**

For peer review only

Therapy Session measure

MUSE Therapist Pack MUSE ARMS Feasibility Trial; IRAS ID: 323903

Session Number:

Date:

Therapy Session measure

Please answer the following questions about the voices you experienced <u>in the past week</u>

<u>How frequent were the voices?</u>

0%	10	20	30	40	50	60	70	80	90	100%
Voices not present					Once a day					Voices always present

<u>Were the voices distressing? How much of the time?</u>

0%	10	20	30	40	50	60	70	80	90	100%
Voices never distressing					Voices were distressing about half of the times					Voices always distressing

If relevant please answer the following questions about the visions you experienced <u>in the past week</u>

<u>How frequent were the visions?</u>

0%	10	20	30	40	50	60	70	80	90	100%
Visions not present					Once a day					Visions always present

<u>Were the vision distressing? How much of the time?</u>

0%	10	20	30	40	50	60	70	80	90	100%
Visions never distressing					Moderately distressing					extremely distressing

<u>Overall, how distressing were the experiences listed above?</u>

0%	10	20	30	40	50	60	70	80	90	100%
not at all distressing					Moderately distressing					extremely distressing

Completed by

Participant ID (MUSE ID Number):	Print Name:	Role:	Signature:	**Completed on (DD/MM/YYYY)**

For peer review only

Therapy Session measure

MUSE Therapist Pack MUSE ARMS Feasibility Trial; IRAS ID: 323903

Session Number:

Date:

Therapy Session measure

Please answer the following questions about the voices you experienced <u>in the past week</u>

<u>How frequent were the voices?</u>

0%	10	20	30	40	50	60	70	80	90	100%
Voices not present					Once a day					Voices always present

<u>Were the voices distressing? How much of the time?</u>

0%	10	20	30	40	50	60	70	80	90	100%
Voices never distressing					Voices were distressing about half of the times					Voices always distressing

If relevant please answer the following questions about the visions you experienced <u>in the past week</u>

<u>How frequent were the visions?</u>

0%	10	20	30	40	50	60	70	80	90	100%
Visions not present					Once a day					Visions always present

<u>Were the vision distressing? How much of the time?</u>

0%	10	20	30	40	50	60	70	80	90	100%
Visions never distressing					Moderately distressing					extremely distressing

<u>Overall, how distressing were the experiences listed above?</u>

0%	10	20	30	40	50	60	70	80	90	100%
not at all distressing					Moderately distressing					extremely distressing

Completed by

Participant ID (MUSE ID Number):	Print Name:	Role:	Signature:	**Completed on (DD/MM/YYYY)**

For peer review only

Therapy Session measure

MUSE Therapist Pack MUSE ARMS Feasibility Trial; IRAS ID: 323903

Session Number:

Date:

Therapy Session measure

Please answer the following questions about the voices you experienced in the past week

How frequent were the voices?

0%	10	20	30	40	50	60	70	80	90	100%
Voices not present					Once a day					Voices always present

Were the voices distressing? How much of the time?

0%	10	20	30	40	50	60	70	80	90	100%
Voices never distressing					Voices were distressing about half of the times					Voices always distressing

If relevant please answer the following questions about the visions you experienced in the past week

How frequent were the visions?

0%	10	20	30	40	50	60	70	80	90	100%
Visions not present					Once a day					Visions always present

Were the vision distressing? How much of the time?

0%	10	20	30	40	50	60	70	80	90	100%
Visions never distressing					Moderately distressing					extremely distressing

Overall, how distressing were the experiences listed above?

0%	10	20	30	40	50	60	70	80	90	100%
not at all distressing					Moderately distressing					extremely distressing

Completed by

Participant ID (MUSE ID Number):	Print Name:	Role:	Signature:	**Completed on (DD/MM/YYYY)**

For peer review only

TAU THERAPY THERAPIST PACK

*Return to research site file for archiving after completion

Adherence Checklist: TAU Therapy Sessions
(Please tick for used in any session)

**Insert Date:**									
**Insert length of session (minutes):**									
	**S1**	**S2**	**S3**	**S4**	**S5**	**S6**	**S7**	**S8**	**Comments**
**Was this a CBT session (Y/N)?**									
CBT Assessment									
Formulation									
Needs based emotional support									
Social Support									
Normalisation									
Stress management									
Psychoeducation*<br><br>**Please describe if related to managing unusual sensory experiences in the comments box*									
Other:									
Other:									
Other:									

	**Completed by**			
Participant ID (MUSE ID Number):	Print Name:	Role:	Signature:	**Completed on (DD/MM/YYYY)**

For peer review only

Adverse Events Checklist

Insert date:									
	**S1**	**S2**	**S3**	**S4**	**S5**	**S6**	**S7**	**S8**	**Comments**
**Adverse events of interest reported?** **Add to Rio/Paris**									
**Serious Adverse Event? NB 24hour reporting deadline**									
**Urgent Safety Measures? NB Phone PI immediately**									

Adverse Events Guidance:

Adverse Events. Record on Rio/Paris for collection by the Unblinded Researcher at the 12wk and 20wk assessment time points that pertain to the following events of Protocol Interest:

- Clinically significant increases in distress and/or psychosis
- Increased harm to self/harm to others
- Increased suicidal ideation/attempts
- Increased use of drugs/alcohol
- Emergency room visits for mental health concerns
- Access to crises services

Serious Adverse Event (SAE): The site Principal Investigator (PI), or delegate shall report all SAEs within 24 hours of becoming aware of the event to the Chief Investigator (CI), or delegate via email to MUSE.ARMS@cntw.nhs.uk using the SAE reporting form. These are events that:

- results in death;
- is life-threatening;
- requires hospitalisation or prolongation of existing hospitalisation;
- results in persistent or significant disability or incapacity;
- consists of a congenital anomaly or birth defect; or,
- is otherwise considered medically significant by the investigator.

Urgent Safety Measures: The site Principal Investigator (PI), or delegate, must inform the CI immediately by telephone (Tel. 01670844670 / alternatively Teams video/voice call for guy.dodgson@cntw.nhs.uk) of urgent safety measures defined above in section 11.5 (early withdrawal/changes to procedure due to safety concerns for staff or participants). This information shall be documented on the Urgent safety reporting form and submitted by email to MUSE.ARMS@cntw.nhs.uk. This is when the following applies:

- Early withdrawal of participant(s) due to safety concerns about the intervention or assessments
- Changes to procedures due to concerns about staff or participant safety

Transition to Psychosis Checklist

Insert date:									
	**S1**	**S2**	**S3**	**S4**	**S5**	**S6**	**S7**	**S8**	**Comments**
**Indication of Transition to Psychosis?** **Add note to Rio/Paris**									

Transition to Psychosis Guidance: The following information suggests potential transition to psychosis for this protocol:

- Clinical diagnosis using standard diagnostic classification systems DSM/ICD
- Clinical diagnosis using ARMS assessment schedule documented in clinical notes
- Transfer to the Early Intervention in Psychosis pathway
- Treated or untreated psychotic episode of one week's duration or longer
- Initiation of treatment with antipsychotics (3 or more weeks of treatment with antipsychotics at a dose of ≥ 5mg haloperidol or equivalent

	**Completed by**			
Participant ID (MUSE ID Number):	Print Name:	Role:	Signature:	**Completed on (DD/MM/YYYY)**

For peer review only

For peer review only

SPIRIT

STANDARD PROTOCOL ITEMS: RECOMMENDATIONS FOR INTERVENTIONAL TRIALS

SPIRIT 2013 Checklist: Recommended items to address in a clinical trial protocol and related documents*

Section/item	Item No	Description	
**Administrative information**			
Title	1	Descriptive title identifying the study design, population, interventions, and, if applicable, trial acronym	✓
Trial registration	2a	Trial identifier and registry name. If not yet registered, name of intended registry	✓
	2b	All items from the World Health Organization Trial Registration Data Set	
Protocol version	3	Date and version identifier	✓
Funding	4	Sources and types of financial, material, and other support	✓
Roles and responsibilities	5a	Names, affiliations, and roles of protocol contributors	✓
	5b	Name and contact information for the trial sponsor	✓
	5c	Role of study sponsor and funders, if any, in study design; collection, management, analysis, and interpretation of data; writing of the report; and the decision to submit the report for publication, including whether they will have ultimate authority over any of these activities	✓
	5d	Composition, roles, and responsibilities of the coordinating centre, steering committee, endpoint adjudication committee, data management team, and other individuals or groups overseeing the trial, if applicable (see Item 21a for data monitoring committee)	✓

For peer review only

**Introduction**			
Background and rationale	6a	Description of research question and justification for undertaking the trial, including summary of relevant studies (published and unpublished) examining benefits and harms for each intervention	
	6b	Explanation for choice of comparators	
Objectives	7	Specific objectives or hypotheses	
Trial design	8	Description of trial design including type of trial (eg, parallel group, crossover, factorial, single group), allocation ratio, and framework (eg, superiority, equivalence, noninferiority, exploratory)	
**Methods: Participants, interventions, and outcomes**			
Study setting	9	Description of study settings (eg, community clinic, academic hospital) and list of countries where data will be collected. Reference to where list of study sites can be obtained	
Eligibility criteria	10	Inclusion and exclusion criteria for participants. If applicable, eligibility criteria for study centres and individuals who will perform the interventions (eg, surgeons, psychotherapists)	
Interventions	11a	Interventions for each group with sufficient detail to allow replication, including how and when they will be administered	
	11b	Criteria for discontinuing or modifying allocated interventions for a given trial participant (eg, drug dose change in response to harms, participant request, or improving/worsening disease)	
	11c	Strategies to improve adherence to intervention protocols, and any procedures for monitoring adherence (eg, drug tablet return, laboratory tests)	
	11d	Relevant concomitant care and interventions that are permitted or prohibited during the trial	
Outcomes	12	Primary, secondary, and other outcomes, including the specific measurement variable (eg, systolic blood pressure), analysis metric (eg, change from baseline, final value, time to event), method of aggregation (eg, median, proportion), and time point for each outcome. Explanation of the clinical relevance of chosen efficacy and harm outcomes is strongly recommended	

For peer review only - http://bmjopen.bmj.com/site/about/guidelines.xhtml

Participant timeline	13	Time schedule of enrolment, interventions (including any run-ins and washouts), assessments, and visits for participants. A schematic diagram is highly recommended (see Figure)	
Sample size	14	Estimated number of participants needed to achieve study objectives and how it was determined, including clinical and statistical assumptions supporting any sample size calculations	
Recruitment	15	Strategies for achieving adequate participant enrolment to reach target sample size	
**Methods: Assignment of interventions (for controlled trials)**			
Allocation:			
Sequence generation	16a	Method of generating the allocation sequence (eg, computer-generated random numbers), and list of any factors for stratification. To reduce predictability of a random sequence, details of any planned restriction (eg, blocking) should be provided in a separate document that is unavailable to those who enrol participants or assign interventions	
Allocation concealment mechanism	16b	Mechanism of implementing the allocation sequence (eg, central telephone; sequentially numbered, opaque, sealed envelopes), describing any steps to conceal the sequence until interventions are assigned	
Implementation	16c	Who will generate the allocation sequence, who will enrol participants, and who will assign participants to interventions	
Blinding (masking)	17a	Who will be blinded after assignment to interventions (eg, trial participants, care providers, outcome assessors, data analysts), and how	
	17b	If blinded, circumstances under which unblinding is permissible, and procedure for revealing a participant's allocated intervention during the trial	
**Methods: Data collection, management, and analysis**			
Data collection methods	18a	Plans for assessment and collection of outcome, baseline, and other trial data, including any related processes to promote data quality (eg, duplicate measurements, training of assessors) and a description of study instruments (eg, questionnaires, laboratory tests) along with their reliability and validity, if known. Reference to where data collection forms can be found, if not in the protocol	
	18b	Plans to promote participant retention and complete follow-up, including list of any outcome data to be collected for participants who discontinue or deviate from intervention protocols	

For peer review only - http://bmjopen.bmj.com/site/about/guidelines.xhtml

Data management	19	Plans for data entry, coding, security, and storage, including any related processes to promote data quality (eg, double data entry; range checks for data values). Reference to where details of data management procedures can be found, if not in the protocol	
Statistical methods	20a	Statistical methods for analysing primary and secondary outcomes. Reference to where other details of the statistical analysis plan can be found, if not in the protocol	
	20b	Methods for any additional analyses (eg, subgroup and adjusted analyses)	
	20c	Definition of analysis population relating to protocol non-adherence (eg, as randomised analysis), and any statistical methods to handle missing data (eg, multiple imputation)	
**Methods: Monitoring**			
Data monitoring	21a	Composition of data monitoring committee (DMC); summary of its role and reporting structure; statement of whether it is independent from the sponsor and competing interests; and reference to where further details about its charter can be found, if not in the protocol. Alternatively, an explanation of why a DMC is not needed	
	21b	Description of any interim analyses and stopping guidelines, including who will have access to these interim results and make the final decision to terminate the trial	
Harms	22	Plans for collecting, assessing, reporting, and managing solicited and spontaneously reported adverse events and other unintended effects of trial interventions or trial conduct	
Auditing	23	Frequency and procedures for auditing trial conduct, if any, and whether the process will be independent from investigators and the sponsor	
**Ethics and dissemination**			
Research ethics approval	24	Plans for seeking research ethics committee/institutional review board (REC/IRB) approval	
Protocol amendments	25	Plans for communicating important protocol modifications (eg, changes to eligibility criteria, outcomes, analyses) to relevant parties (eg, investigators, REC/IRBs, trial participants, trial registries, journals, regulators)	
Consent or assent	26a	Who will obtain informed consent or assent from potential trial participants or authorised surrogates, and how (see Item 32)	
	26b	Additional consent provisions for collection and use of participant data and biological specimens in ancillary studies, if applicable	

For peer review only - http://bmjopen.bmj.com/site/about/guidelines.xhtml

Confidentiality	27	How personal information about potential and enrolled participants will be collected, shared, and maintained in order to protect confidentiality before, during, and after the trial	
Declaration of interests	28	Financial and other competing interests for principal investigators for the overall trial and each study site	
Access to data	29	Statement of who will have access to the final trial dataset, and disclosure of contractual agreements that limit such access for investigators	Protocol paper<br>Data management<br>* See Note Below
Ancillary and post-trial care	30	Provisions, if any, for ancillary and post-trial care, and for compensation to those who suffer harm from trial participation	IRAS<br><br>NHS Insurance<br>* See Note Below
Dissemination policy	31a	Plans for investigators and sponsor to communicate trial results to participants, healthcare professionals, the public, and other relevant groups (eg, via publication, reporting in results databases, or other data sharing arrangements), including any publication restrictions	
	31b	Authorship eligibility guidelines and any intended use of professional writers	Protocol Paper – ICMJE<br>* See Note Below
	31c	Plans, if any, for granting public access to the full protocol, participant-level dataset, and statistical code	
**Appendices**			
Informed consent materials	32	Model consent form and other related documentation given to participants and authorised surrogates	
Biological specimens	33	Plans for collection, laboratory evaluation, and storage of biological specimens for genetic or molecular analysis in the current trial and for future use in ancillary studies, if applicable	N/A

*It is strongly recommended that this checklist be read in conjunction with the SPIRIT 2013 Explanation & Elaboration for important clarification on the items. Amendments to the protocol should be tracked and dated. The SPIRIT checklist is copyrighted by the SPIRIT Group under the Creative Commons "Attribution-NonCommercial-NoDerivs 3.0 Unported" license.

For peer review only

*The SRIRIT checklist has been carefully reviewed against the trial protocol and associated documentation and the explanation of the protocol in the protocol paper submitted.
To provide further explanation on three items where more consideration could be given within the protocol itself are:

(i) item 29, refers to a statement of who will have access to the final trial dataset, and disclosure of contractual agreements that limit such access for investigators. This is an NIHR funded feasibility trial with no DMEC within its feasibility stage. We do not have contractual agreements that limit data access, other than to limit access to confidential information as this is restricted and explained in the participant facing documents. We have detailed in the protocol paper how data is input, stored, and transferred prior to analysis to allow for audit and monitoring in accordance with the trial monitoring plan.

(ii) item 30 refers to provisions, if any, for ancillary and post-trial care, and for compensation to those who suffer harm from trial participation. As this is a psychological therapies trial there are no biological risks, however we are conscious of the possibility of increased psychological risks and so we have developed clear safety reporting procedures for serious adverse events and for urgent safety measures should these arise, which abide by HRA and UK Research Ethics Committee standard procedures. We have also written into the protocol procedures for collecting adverse events that do not hit the seriousness criteria, so that these can be reported in the main outcomes paper. Our research has NHS insurance and this is clear on the IRAS application document. Any post-trial care would be standard NHS care;

(iii) item 31b refers to authorship eligibility guidelines and any intended use of professional writers. We do not use professional writers. We have not written authorship eligibility into the protocol, however the lead writers for the different papers have been identified in advance. We have followed the ICMJE recommendations for authorship and have noted this in the contributors section of the protocol paper.

All other items of the SPIRIT checklist are available within the protocol and Research Ethics Committee approved participant facing documents as appropriate.

For peer review only

CONSORT Abstract Checklist (Clarke et al 2008).

The Abstract has been reviewed in accordance with the CONSORT Abstract Checklist and is in adherence, see below. As this is a protocol paper of a trial open and in its very early stages of data collection, no results are currently available. TheMUSE ARMS Feasibility Trial opened to participants on 14.04.2023.

Item	Description	
Title	Identification of the study as randomised	✓
Authors*	Contact details for the corresponding author	✓
Trial design	Description of the trial design (eg, parallel, cluster, non-inferiority)	✓
Methods		
Participants	Eligibility criteria for participants and the settings where the data were collected	✓
Interventions	Interventions intended for each group	✓
Objective	Specific objective or hypothesis	✓
Outcome	Clearly defined primary outcome for this report	✓
Randomisation	How participants were allocated to interventions	✓
Blinding (masking)	Whether or not participants, care givers, and those assessing the outcomes were blinded to group assignment	✓
Results		
Numbers randomised	Number of participants randomised to each group	
Recruitment	Trial status	
Numbers analysed	Number of participants analysed in each group	
Outcome	For the primary outcome, a result for each group and the estimated effect size and its precision	
Harms	Important adverse events or side-effects	
Conclusions	General interpretation of the results	
Trial registration	Registration number and name of trial register	✓
Funding	Source of funding	✓

*For conference abstracts.

Reference:

CONSORT for reporting randomised trials in journal and conference abstracts
Clarke, Mike;Moher, David;Wager, Elizabeth;Middleton, Philippa;Altman, Douglas G;Schulz, Kenneth F
The Lancet; Jan 26-Feb 1, 2008; 371, 9609; ProQuest
pg. 281

BMJ Open

Use of a targeted, computer/web-based guided self-help psychoeducation toolkit for distressing hallucinations (MUSE) in people with an At Risk Mental State for psychosis: protocol for a randomised controlled feasibility trial

Journal:	*BMJ Open*
Manuscript ID	bmjopen-2023-076101.R1
Article Type:	Protocol
Date Submitted by the Author:	12-Jun-2023
Complete List of Authors:	Hamilton, Jahnese; Cumbria Northumberland Tyne and Wear NHS Foundation Trust, Research and Development; Newcastle University<br>Arnott, Bronia; Newcastle University<br>Aynsworth, Charlotte; Cumbria Northumberland Tyne and Wear NHS Foundation Trust<br>Barclay, Nicola; Cumbria Northumberland Tyne and Wear NHS Foundation Trust, Central At-Risk Mental State Service<br>Birkett, Lauren; Cumbria Northumberland Tyne and Wear NHS Foundation Trust<br>Brandon, Toby; Northumbria University<br>Dixon, Lyndsey; Cumbria Northumberland Tyne and Wear NHS Foundation Trust<br>Dudley, Robert; University of York; Cumbria Northumberland Tyne and Wear NHS Foundation Trust<br>Einbeck, J; Durham University<br>Gibbs, Christopher; Cumbria Northumberland Tyne and Wear NHS Foundation Trust<br>Kharatikoopaei, Ehsan; Durham University; Manchester Metropolitan University<br>Simpson, Jennifer; Tees Esk and Wear Valleys NHS Foundation Trust<br>Dodgson, Guy; Cumbria Northumberland Tyne and Wear NHS Foundation Trust<br>Fernyhough, Charles; Durham University
**Primary Subject Heading**:	Mental health
Secondary Subject Heading:	Evidence based practice
Keywords:	MENTAL HEALTH, PSYCHIATRY, Randomized Controlled Trial

Use of a targeted, computer/web-based guided self-help psychoeducation toolkit for distressing hallucinations (MUSE) in people with an At Risk Mental State for psychosis: protocol for a randomised controlled feasibility trial

Abstract

Introduction: Individuals who access At Risk Mental State (ARMS) services often have unusual sensory experiences and levels of distress that lead them to seek help. The Managing Unusual Sensory Experiences (MUSE) treatment is a brief symptom targeted intervention that draws on psychological explanations to help account for unusual experiences. Practitioners use formulation and behavioural experiments to support individuals to make sense of their experiences and enhance coping strategies. The primary objective of this feasibility trial is to resolve key uncertainties before a definitive trial and inform parameters of a future fully powered trial.

Methods and analysis: 88 participants aged 14-35 accepted into ARMS services, experiencing hallucinations / unusual sensory experiences which are considered by the patient to be a key target problem will be recruited from UK NHS sites and randomised using 1:1 allocation (stratified by site, gender, and age) to either 6-8 sessions of MUSE or time-matched treatment as usual. Participants and therapists will be unblinded, research assessors are blinded. Blinded assessment will occur at baseline, 12weeks, and 20weeks post randomisation. Data will be reported in line with CONSORT. Primary trial outcomes are feasibility outcomes, primary participant outcomes are functioning and hallucinations. Additional analysis will investigate potential psychological mechanisms and secondary mental wellbeing outcomes. Trial progression criteria follows signal of efficacy and uses an analytic framework with a traffic-light system to determine viability of a future trial. Subsequent analysis of the NHS England Mental Health Services Data Set 3 years post-randomisation will assess long-term transition to psychosis.

Ethics and dissemination: This trial has received Research Ethics Committee approval (Newcastle North Tyneside 1 REC; 23/NE/0032). Participants provide written informed consent; young people provide assent with parental consent. Dissemination will be to ARMS Services, participants, public and patient forums, peer-reviewed publications and conferences.

Trial registration: ISRCTN58558617.

For peer review only

Strengths and limitations of this study

- This is a feasibility RCT, results will address key uncertainties to inform a future large-scale trial, including sample size and design decisions.
- The MUSE intervention toolkit, the trial design and participant facing materials have been developed with substantial input from people with lived experience.
- This study is distinctive in exploring potential causal cognitive mechanisms in an At Risk Mental State population who have unusual sensory experiences.
- There is no gold standard treatment to compare the intervention to, so controlled time-matched treatment as usual is selected as the comparator.
- The follow-up period is short (20 weeks post randomisation), therefore longer-term participant impacts will not be fully assessed; however long-term transition to psychosis will be examined via the Mental Health Services Data Set.

INTRODUCTION

At Risk Mental State (ARMS) describes presentations that indicate a potential prodromal stage of psychosis, or risk of psychosis, with around 25% of ARMS individuals converting to psychosis within 36 months (1). The importance of working with these individuals to target possible unhelpful beliefs in development, reduce distress, support healthy functioning, and potentially to prevent the development of full psychosis is widely advocated (2, 3).

The presence of unusual sensory experiences, such as hearing voices and seeing visions (hallucinations), may not in themselves indicate mental ill health as there may be common underlying psychological mechanisms or a continuum of experience from benign, everyday experiences to more severe hallucinations that require treatment (4). However, increased frequency and intensity of hallucinations, alongside distress and a decline in functioning, are linked to transition to psychosis and are threshold criteria in scales recommended in ARMS services (5, 6). Intervening to reduce the distress of unusual sensory experiences and offer explanations of the possible mechanisms behind these experiences may be key in preventing transition to psychosis (3, 7).

Current UK NICE guidelines recommend that people meeting ARMS criteria should be referred for specialist assessment and offered Cognitive Behavioural Therapy (CBT) to reduce the risk of developing psychosis (8-10). While approaches involving CBT and CBT with supportive therapy show promise in ARMS, the evidence for CBT improving functioning and mental state, or reducing

For peer review only

progression to psychosis, is inconclusive (11-13). No specific psychological intervention has been identified as having superior effectiveness in its treatment; there is no gold standard treatment (11, 14, 15). ARMS services therefore need to further assess interventions that indicate potential benefit. Robust clinical trials are needed to determine benefits versus risk profiles, accessibility and cost effectiveness (11, 13, 16).

Treatment development would be improved if they addressed key causal mechanisms leading to distressing experiences, and adapted treatment to the needs of different age groups (17, 18). Taking a staged or stepped approach to psychological intervention is good practice, usually with CBT and needs-based interventions prior to pharmacology (8, 18, 19). There is scope for research into briefer approaches implemented prior to CBT in ARMS services, and emerging evidence from early intervention in psychosis research that inclusion of briefer targeted evidence-based interventions prior to CBT may result in a reduction of need for more in-depth CBT, as people better understand their experiences and have less need for interventions (20).

Through extensive multidisciplinary research into voice hearing, clinically embedded research with patients who are indicating at risk state for psychosis, and studies of first episode psychosis, we have developed a targeted, computer/web-based guided self-help psychoeducation toolkit for distressing hallucinations (MUSE) (7, 21). MUSE endeavours to provide scientific and normalising explanations that may provide acceptable and helpful understandings of an individual's unusual sensory experiences and help to prevent more delusional explanations from developing. MUSE has been trialled with an ARMS patient group in a non-randomised study (21) and shown to be acceptable with good participant satisfaction with the therapy. We intend to assess MUSE through a series of trials to determine patient benefit and possible impact on progression to psychosis in patients at high risk. We will also seek to learn more about whether change relates to target mechanisms underlying hallucination subtypes(22). This could be important for further refinement of treatment.

Objectives

Primary objective

To conduct an ISRCTN-registered feasibility randomised controlled trial to resolve key feasibility uncertainties and inform the parameters of a future trial, to investigate the preliminary effect of MUSE + treatment as usual (TAU) versus time-matched supportive psychotherapy TAU on general functioning (assessed using the Social and Occupational Functional Assessment Scale [SOFAS](23), and mental state related to frequency and distress of unusual sensory experiences and false beliefs (assessed using the Psychotic Symptom Rating Scales [PSYRATS](24) total score, and sub-scales

For peer review only

Hallucinations and Attribution(25)) in ARMS patients post therapy and at five-month post randomisation follow-up.

Secondary objectives

To explore additional treatment effects on unusual sensory experiences, anxiety, depression, and quality of life, and whether there are indications of other factors (sleep disturbance and trauma) influencing treatment effects.

To test feasibility of collecting measures of psychological mechanisms, including psychological and personal (phenotypical) factors implicated in the clinical course of hallucinations. To analyse which psychological mechanisms are influenced by the treatment and contribute to its clinical effect and inform a future investigation of whether any efficacy of MUSE is through impact on these mechanisms.

To collect routine data for a future records investigation testing feasibility of tracking transition to psychosis through medical databases (hospital records/Mental Health Services Data Set (MHSDS)), to examine which features of MUSE (presenting, treatment response and mechanistic) are most relevant to psychosis prevention.

METHODS AND ANALYSIS

Trial design and flowchart

This is a feasibility trial employing a prospective randomised, open-label, observer blinded, endpoint design assessing a targeted, computer/web based guided self-help psychoeducation toolkit for distressing hallucinations (MUSE)+TAU (6 – 8 sessions) compared to a TAU time-matched control (also referred to as supportive psychotherapy) (6 – 8 sessions) offered by a multi-disciplinary team which includes needs based emotional support, psychoeducation and stress management, aiming to reduce distress from hallucinations and improve functioning, in people with an At Risk Mental State (ARMS) for psychosis in UK secondary care mental health services.

The trial has received NHS Research Ethics positive opinion (23/NE/0032) and Health Research Authority Approvals and is registered with the ISRCTN registry (ISRCTN58558617, registered 09.05.2023). Two substantial amendments followed first approval and were obtained prior to first participant consent: Amendment 1 notably added in an unvalidated Preferences Questionnaire for therapeutic intervention, and changed an anxiety self-report questionnaire over to use the State-Trait Anxiety Inventory – Short Form (STAI-Short Form)(26-28). Amendment 2 replaced a longer

For peer review only

dissociative experiences questionnaire for the 8-item Brief Dissociative Experiences Scale [DES-B]—Modified(29, 30).

The trial has an independent trial steering committee (TSC) and Lived Experience Advisory Panel (LEAP) facilitated by a co-applicant for the study with lived experience of psychosis.

Participants

Recruitment will be via NHS secondary care mental health clinical teams providing ARMS services. Patients who potentially meet the eligibility criteria for the trial, and their parent/guardian where appropriate if under 18 years, will be informed of the study by a member of their clinical team. Participants will be checked for eligibility prior to informed consent via discussion with referring teams and in the participant-researcher discussion prior to giving informed consent. Participant Information Sheets will be provided at least three days prior to the informed consent meeting. Written informed consent in adherence to principles of Good Clinical Practice (GCP) will be obtained prior to participation. For participants aged 14 and 15 years old, Parent/Guardian informed consent with child assent will be taken; this option of assent with Parent/Guardian consent will also be made available to participants aged 16 and 17 years old due to their potential vulnerability and the governing UK law which classes a minor as someone who is under 18 years old. Verbal consent form will be used for participants with literacy challenges. Interpreters and translated consent forms will be available for participants who do not speak English. Participants will be given £15 honorarium for each assessment time-point.

Trial eligibility criteria

Inclusion criteria

- in contact with an ARMS service or accepted on an ARMS pathway by EIP services
- aged 14–35
- hallucinations / unusual sensory experiences scoring at least 3 on the Perceptual Abnormalities Subscale of the Comprehensive Assessment of At-Risk Mental States (CAARMS)(6)
- hallucinations considered by the patient to be a key target problem
- judged to have been clinically stable for the preceding 2 weeks

Exclusion criteria

- intellectual disability or severe cognitive dysfunction affecting ability to engage with research materials

For peer review only

- lacking capacity to give informed consent

Randomisation and blinding

Eligible participants who have completed baseline assessments will be randomised and subsequent assessments will be scheduled from the point of randomisation. An independent web-based randomisation service (sealedenvelope.com) will be used for the trial. Randomisation will be in the ratio 1:1 to the two groups: MUSE+TAU (intervention) or TAU time-matched control (supportive psychotherapy+TAU (control). Randomisation will be stratified by site, gender (M/F/Other) and age (14–17years/18–35 years inclusive). Randomisation allocation will be independent and dynamically generated using a randomised modified minimisation method (31) to assure allocation concealment along with preservation of allocation ratio. Randomisation allocation is made known to the CI and site PIs, the Trial Coordinator(s) and the trial therapists only at the point of randomisation, by email.

Research assessors for the trial will be blind to the allocation throughout the trial. Clinicians, therapists and participants will be unblind. Trial statisticians will be partially blind; In the first instance, for the analyses and reporting of main outcomes of the trial the Statisticians will be fully blind. However, for secondary sensitivity analysis such as impact of number of MUSE sessions on effect size) and the mechanisms investigations, the Statisticians will be required to view which participants received MUSE treatment.

Assessments

Assessors blinded to trial allocation will complete participant assessments at baseline, 12 weeks post randomisation, and 20 weeks post randomisation (See Table 1). Sociodemographic information will be collected from the participant at baseline only (CSRI questions 1-3.5 as amended for the trial(32)).

Table 1. Trial assessments and key participant procedures schedule

Assessments/ procedures	Participant identification	Enrolment & baseline	Randomisation	Intervention Weeks 1-12	12 weeks post randomisation (+/- 10days)	20 weeks post randomisation (+/- 10days)
Recruitment and eligibility discussions	X					
Informed consent		X				
CSRI Sociodemographic Q1-3.5		X				
Randomisation			X			
MUSE & TAU / TAU Intervention				←→		
**Blinded assessments**						

For peer review only

MUSE ARMS Primary Outcome Measures: SOFAS & PSYRATS		X			X	X
CSRI service use Q4.1-4.4		X				
CSRI Q4.5 criminal justice services and Q5 medication		X			X	X
MUSE ARMS Secondary Outcome Measures: CAARMS-PA, PHQ-9, GAD-7, ReQoL-20, ISI, ITQ/ITQ-CA, MMHQ		X			X	X
Subtype Measures & Cognitive Tasks (1-2 subtypes selected per participant)		X			X	X
Treatment preference		X				
**Unblinded assessments**						
CSRI service use at follow-up Q4.1-4.4					X	X
Transition to Psychosis data					X	X
Adverse Event (AE) data					X	X
Therapeutic Alliance STTS-R					X	
Participants interviews (Withdrawals sub-sample)				↔	↔	
Participants interviews (MUSE completers sub-sample)					↔	↔
Participants interviews (TAU sub-sample)					↔	↔
Therapists interviews (sub-sample)					↔	↔

Primary indicators of outcome

The primary outcome measures are: (i) Feasibility outcomes, including qualitative interviews; (ii) General functioning assessed using the SOFAS(23), a clinician/clinical researcher rated single-item scale; (iii) Target problem hallucinations assessed using the PSYRATS(24) (hallucination total) clinician/clinical researcher rated interview, and; (iv) Distress and attribution dimensions of target problem assessed using the PSYRATS (25).

Secondary assessments

Additional assessments will be the clinician/clinical researcher administered semi-structured interview CAARMS subscale of Perceptual Abnormalities(6) to elicit further detail about the nature of unusual experiences. Self-reported measures will rate depression symptom severity (PHQ-9(33)), anxiety (GAD-7(34)), quality of life (ReQoL-20(35)), sleep difficulties (ISI(36)), and trauma (ITQ/ITQ-CA(37, 38)). An unvalidated measure, the Multi-Modal Hallucinations Scale (MMHS) will be used to assess cross-modal sensory experiences.

Service use

Assessment of potential contamination of the MUSE intervention within the TAU condition, of other psychological therapies use within the treatment arms, and the need for additional interventions

For peer review only

beyond the treatment phase will be captured using the CSRI(32) (as amended for the trial) at baseline, 12 week and 20 week follow-up. CSRI service use data at 12 weeks and 20 weeks will be collected from medical notes by the unblinded researcher to preserve blinding of research assessors.

Mechanisms assessment

To assess further information on mechanisms, subtype measures & cognitive tasks will be selected per participant for 1 to 2 hallucination subtypes: (i) Inner speech, using the Varieties Of Inner Speech Questionnaire (VISQ-R)(39), and computerised cognitive tasks Auditory Signal Detection Task and Auditory Reality Monitoring Source Memory Task (40, 41); (ii) Memory, using the Brief Dissociative Experiences Scale —Modified (DES-B)(29, 30) and computerised cognitive task Inhibition of Currently Irrelevant Memories (ICIM)(42); (iii) Hypervigilance, using the State-Trait Anxiety Inventory – Short Form (STAI-Short Form)(26-28), and computerised cognitive Jumbled Speech Task (JST)(43, 44), and; (iv) Visual, using the visual section of the Plymouth Sensory Imagery Questionnaire (Psi-Q)(45) , and computerised cognitive tasks Visual Signal Detection(46), Visual Reality Monitoring(47) and Face Pareidolia Task(46). Researchers receive training on subtype selection. Selections are monitored and evaluated against MUSE therapist subtype selections to assess selection reliability and potential training needs.

Acceptability assessment

To assess therapy preference, satisfaction and acceptability of the intervention, participants will be asked about treatment preferences at baseline using a study specific preferences questionnaire (see supplementary materials 1), and treatment satisfaction post intervention using the Satisfaction with Therapy and Therapist Scale-Revised (STTS-R)(48, 49). Qualitative interviews with participants and trial therapists will further explore experience of MUSE, TAU, and trial procedures.

Long-term outcomes

Long-term transition to psychosis outcomes will be collected 3 years post baseline via the NHS England Mental Health Services Data Set (MHSDS).

Data management

Interview/clinical assessments data will be scored following the visit and entered onto Qualtrics by the researcher. Source data will be retained in the site file. Self-report data will be entered directly onto Qualtrics during visits using participant ID and visit as markers. Unblinded data on service use will be entered onto Qualtrics at the visit time points. Qualtrics outputs and computerised cognitive task data will be downloaded and date stamped at regular intervals to allow data audit. The full data

For peer review only

set will be transferred in its anonymous form to the stats team upon completion of data lock at the end of the trial. Trial monitoring at sites will occur across the life cycle of the trial and will follow the Sponsor approved data monitoring plan.

Intervention: Managing Unusual Sensory Experiences (MUSE) + TAU

The MUSE intervention is a novel targeted, computer/web-based guided self-help psychoeducation toolkit and psychological treatment manual for managing distressing hallucinations in mental health, developed and owned jointly by Durham University and CNTW. Patients work with experienced therapists, under expert supervision, who utilise the MUSE package within therapy sessions to develop a formulation explaining the development of hallucinations and foster new skills and strategies for their management. The MUSE treatment is divided into the following modules: What are Voices?; How the Mind Works; Assessment (of participant subtype); Inner Speech; Memory and Trauma; Hypervigilance; and Sleep (see Dudley, Dodgson (50) for details).

Six to eight 1-hour sessions will be offered weekly by experienced therapists who are clinical psychologists or psychological therapists. Therapists will be accredited or working towards accreditation by the British Association of Behavioural and Cognitive Psychotherapists (BABCP), employed by the ARMS service and have experience of MUSE, receiving clinical supervision and fortnightly MUSE supervision. MUSE is loaded onto therapists smart tablet/NHS laptop (not reliant on Wi-Fi) and is available to patients via the CNTW website between sessions. No personal data are recorded or stored on MUSE toolkit.

Session by session measures will be used as part of the MUSE package to enable therapists to monitor any variations in hallucination frequency and distress that may have a bearing on the selection of module used or revisited during the treatment session.

Therapists will be asked to complete adherence checklists for each session contained within a per-participant MUSE Therapist Pack (see supplementary materials 2). With consent, each session will be audio-recorded to enable independent review by the site Principal Investigator or delegated Clinical Lead of a random 10% sample to ensure fidelity to protocol within and across sites.

Control condition: time-matched control + TAU

To control for risk of bias from an undefined comparative treatment, and potential bias from dose effects, a time-matched TAU is included(11, 51). In order to match the comparative brief intervention to usual practice within ARMS services, components of care were identified in an

For peer review only

engagement meeting with ARMS service leads. These common core components could be described as supportive psychotherapy or ongoing care (needs based emotional support, psychoeducation, normalisation and stress management) and were outlined as the interventions used by therapists as part of their normal clinical toolkit, alongside routine multi-disciplinary care from the team. Patients work with different therapists who are ARMS clinicians and are not trained in MUSE. These clinicians will receive supervision on their practice through the routine supervision arrangements of their service and will record the interventions used within a per-participant TAU Therapist Pack (see supplementary materials 3). We will investigate how frequently and consistently these supportive psychotherapy interventions are offered to inform whether these interventions could act as a comparator intervention in future trials. This arm will be time-matched controlled, however variation across services precluded using this comparator being defined as a controlled *intervention*. Number of sessions received in this group will be recorded for analysis.

Both groups: TAU

In addition to the trial allocated intervention (MUSE or TAU time-matched control supportive psychotherapy), both treatment groups will also receive additional usual care as clinically indicated. No treatments will be withheld on account of being part of the trial. This includes regular monitoring, signposting to appropriate local services for unmet needs, social support and crisis management when required from the multi-disciplinary team. CBT is also a core intervention recommended by NICE Guidance and offered across ARMS services. However, in practice it is not always offered to all service users. CBT may form part of the care in both conditions as part of usual care. We will investigate the number of CBT sessions received by participants in both groups and investigate whether MUSE impacts on the number of sessions required. Additional care will be based on clinical judgement and will be recorded for both arms of the study. These additional elements of care, including interventions and contacts that occur beyond the MUSE/time-matched period will be analysed for variations and similarities in the care received between the two groups.

Analysis

Analyses will follow intention to treat principles, with data analysed according to randomisation irrespective of treatment received. A full statistical analysis plan (SAP) will be developed for the outcome measures and agreed with TSC before the end of data collection. Data will be reported in line with the Consolidated Standards of Reporting Trials (CONSORT)(52), see figure 1.

Sample size

For peer review only

As this is a feasibility trial there is no formal sample size calculation, interim analyses or stopping rule. The trial aims to recruit 88 participants with 70 participants completing the study (allowing for 20% attrition) to be able to robustly calculate the sample size for a definitive trial(53).

Statistical analysis

Feasibility outcomes will be analysed primarily: the ability of the trial to recruit participants, who reflect the diversity within the region, and meet study inclusion criteria over the 9-month recruitment period, who complete assessment measures collected at baseline, post intervention and follow-up, until all participants complete the follow-up assessment or withdraw.

Descriptive statistics within each randomised group will be presented for baseline and follow-up points. All data will be summarised as appropriate using mean ± standard deviation and median ± interquartile range for continuous outcome data; frequency and percentages for binary or categorical data; and rate for count data. Analysis will be via the latest version of R.

The signal of efficacy will be determined by examining the effect of each arm (MUSE versus supportive psychotherapy) on outcomes measures, estimated as change from baseline.

The effects will be estimated using generalised linear mixed effect models with the appropriate distribution and link function. Normal distributions with identity link will be used for continuous outcomes, and negative-binomial distributions with log link for count data outcomes. All binary or categorical outcomes will be analysed using generalised estimating equations (GEE). The mixed-effects models and GEE account for the repeated measurements per participant over the follow-up time points. All models will be adjusted for treatment arms and stratification variables. The mixed model approach taken will allow identifying the individual effect of the two interventions with relation to their baseline, as well as the difference in their effects through an interaction parameter of time and intervention. This can be considered as a model-based difference-in-difference analysis.

These models will be used mainly to estimate relevant parameters, since the trial is not powered for null hypothesis significance-testing. That is, while we are interested in identifying the magnitude of the signal of efficacy, we will not attempt to prove its significance.

In addition, a mediation analysis will examine how the different mechanism components mediate the estimated impact of the interventions on the primary outcomes, and a complier average causal effects (CACE) analysis will determine the impact of the number of sessions on the MUSE effect.

If data are missing for a particular participant and outcome measure, this participant will be excluded from the analysis, for this outcome measure only, without further adjustment for

For peer review only

missingness. However, the effect of missing data will be investigated additionally by sensitivity analysis using tabulation of rate of missingness across trial arms and imputation methods.

Qualitative analysis

Audio-recordings will be transcribed and analysed (in NVivo software). Interview transcripts will be analysed using thematic analysis(54) allowing a transparent, replicable and robust process and demonstration of reflexivity and quality. Transcripts will be coded by two researchers until coding reliability is established; coding will then be conducted by one researcher, with reliability checks by the qualitative lead. Data will be extracted into a framework matrix, summarising data by category from individual transcripts, with quotations selected as illustrative exemplars. Initial findings from the qualitative analyses will be presented to LEAP for feedback on interpretation.

Health economics analysis

As a feasibility study, we are not undertaking a formal economic evaluation at this stage but will inform a health economic evaluation in a future definitive trial by piloting the ReQoL-UItility Index with the ReQoL-20 data for health economic analysis calculation.

Criteria for proceeding to a future trial

The signal of efficacy is dependent upon the primary outcome data (SOFAS, PSYRATS Total, PSYRATS distress, PSYRATS attribution) and follows: i) Go: primary outcome data suggest the intervention may show an effect indicating clinical value warranting further investigation; ii) Refine: primary outcome data indicate no measure of effect, but one or more secondary outcomes indicates an effect; iii) Stop: no effect across any outcomes.

The trial progression criteria will follow signal of efficacy and cover domains of research delivery, therapy engagement and fidelity, and safety. The criteria were influenced by LEAP and TSC input and sign-off. Trial progression criteria uses an analytic framework with a traffic-light system (see supplementary Table 1). Progression will depend upon: (i) All Green outcomes: no/minor revisions prior to next development of the trial, or; (ii) One or more Amber (but not Red) outcomes: If feasible, substantial alterations to the trial protocol, assessments or intervention, supported by the qualitative work-stream and discussed with TMG and TSC prior to the next development of the trial or; (iii) If one or more Red outcomes result then the trial is unlikely to progress at that site or very substantial amendments are needed. The mechanism measures and tasks will also be reviewed for sensitivity to change and reliability to inform the next development of the trial.

For peer review only

Decisions regarding any changes will consider the ADePT decision-making process to address potential problems with intervention, clinical setting, or trial design that may be relevant in either a trial setting or real world context. We will use qualitative data to contextualise our progression criteria, to ensure that the participant feedback informs our understanding of our research delivery and signal of efficacy.

Adverse events

Serious Adverse Events (SAEs) are defined as: results in death; is life-threatening; requires hospitalisation or prolongation of existing hospitalisation; results in persistent or significant disability or incapacity; results in congenital anomaly or birth defect; or is otherwise considered medically significant by the investigator. Any SAEs shall be assessed immediately for trial relatedness and expectedness and reported to the Sponsor. Any related and unexpected SAEs and any Urgent Safety Measures (defined as: early withdrawal of participant(s) due to safety concerns about the intervention or assessments, or; changes to procedures due to concerns about staff or participant safety) shall be reported immediately to the Sponsor and Research Ethics Committee in accordance with Health Research Authority governance regulations (See: https://www.hra.nhs.uk/approvals-amendments/managing-your-approval/safety-reporting/) and Sponsor standard operating procedures.

Adverse events will be recorded for all participants where the event relates to mental state, with focus on clinically significant: a) increases in distress and/or psychosis; b) increased harm to self/harm to others; c) increased suicidal ideation/attempts; d) increased use of drugs/alcohol; e) emergency room visits for mental health concerns; f) access to crises services.

Patient and public involvement

The MUSE intervention, the trial design and participant facing materials and the grant application have benefitted from input by individuals with lived experience. To ensure a retained focus on patients, a Lived Experience Advisory Panel (LEAP) led by a co-applicant with personal lived experience of psychosis was established and meets monthly in a mixture of online and face-to-face formats throughout the lifetime of the trial. The study specific Preferences questionnaire was collaboratively developed with the LEAP. The outcome measures and the topic guides were piloted with LEAP members and amended following feedback. The LEAP were consulted on the potential ethical issues of the trial and the trial progression criteria. Members of the LEAP group will also co-facilitate qualitative interviews, help disseminate study findings, and enable patient experience to inform design of future research and any revisions of the treatment. Two LEAP members are part of

For peer review only

the TSC, with one taking a lead on trial procedures and the other on the inclusion of under-served groups. Compensation for work done is given in accordance with NIHR PPI guidelines (https://www.nihr.ac.uk/documents/ppi-patient-and-public-involvement-resources-for-applicants-to-nihr-research-programmes/23437).

ETHICS AND DISSEMINATION

This trial has obtained NHS Research Ethics Committee (REC) positive opinion from Newcastle North Tyneside 1 REC (reference: 23/NE/0032), and UK Health Research Authority approval (IRAS project ID: 323903). Participants are provided with Participant Information at least three days prior to providing informed consent. Participants provide written informed consent; young people provide assent with parental consent (see supplementary materials 4-6). The research Sponsor is Cumbria, Northumberland, Tyne and Wear NHS Foundation Trust (CNTW).

An anonymised version of the main outcome quantitative data and mechanisms data will be available either in open access as encouraged by peer review publications or from the trial team on reasonable request with publication of the trial outcomes paper and mechanisms paper.

The research outcomes shall be submitted for peer review open access publications. Anonymised data will be made available in a repository. Trial outcomes, mechanisms evaluations, and long-term outcomes will be reported on.

The trial outcomes: The feasibility trial outcomes will report on feasibility outcomes and the candidate primary outcome measures (SOFAS and PSYRATS). Secondary reporting will detail the secondary treatment effects and influence of moderators. Additional reporting will detail treatment integrity: data on treatment adherence to the model (sessions checklist data); exposure of participants to the interventions and additional treatments within usual care (CSRI data); the quality of treatment delivered and responsiveness of participants as reflected on by therapists and participants (STTS-R data, qualitative data); and the programme differentiation between the novel intervention arm and the usual care arm (CSRI data).

Mechanisms will be reported on the analysis of secondary assessments for the purposes of informing which aspects of patient presentation the MUSE intervention works with, and informing the outcome measures in a future efficacy and mechanisms trial.

Long-term transition to psychosis paper: Long-term transition to psychosis through the MHSDS/medical records exploratory feasibility analysis will report which features of MUSE

For peer review only

(presentation, treatment response, mechanistic) are indicated as most relevant to psychosis prevention.

Trial status

The trial opened to recruitment at the two planned NHS sites on the 14th April 2023 (Cumbria, Northumberland, Tyne and Wear NHS Foundation Trust) and 21st April 2023 (Tees, Esk and Wear alley NHS Foundation Trust). First participant randomisation (enrolment) was on 10th May 2023. Final participant facing procedures are due to be completed by end of June 2024. The study will finish at NHS research sites after the final assessment with the final participant is completed and the monitoring close-out visit has occurred at site.

*** ***

Acknowledgements

We are grateful to our Sponsor, Cumbria, Northumberland, Tyne and Wear NHS Foundation Trust and the sponsorship team for their backing and support, and to the many individuals in the collaborating institutions who have enabled this trial to run. We are grateful to the dedicated involvement and contributions of the MARS Lived Experience Advisory Panel Group members, all who wish to remain anonymous and those who are happy to be named. Thank you to Gayl McCain, Katie Rumney, Helen Errington, Patricia Higgins and the whole group. Thanks is noted especially to all of the people who over the course of this research agree to receive information on the study and consider whether would like to take part. A number of people gave their time and expertise in reviewing iterations of the trial development and supporting the trial through the various funding and approvals systems, who include Stephanie Common, Angus Williams, and others, and our appreciation is noted. The study has also benefitted from the technical contributions of the psychologists David Smailes (ORCID ID: 0000-0002-0455-070X) and Peter Moseley (ORCID ID: 0000-0002-9284-2509) who respectively built the computerised cognitive tasks used in the trial, we are grateful for this contribution. We would not be able to run the trial without the daily contribution also from all of the trial researchers on the ground who work within the NHS trusts to support the day to day running of trial operations, communication with participants, recruitment to the research and to conduct research assessments; thank you to Michael Kelly, Abbey Underwood, Alexandra Carne, Vickie Larry, Sarah Bentley, Emily Clare, Melanie Rowe, Victoria Wilson, Sarah Garner and others. Tremendous thanks is also given to all of the therapists who work into the NHS ARMS

For peer review only

services who are delivering the interventions for this study, Carina Ewels, Eloise Todd, Louise Ross, Andrew Weightman, Claire Mason, Toyah Lebert, Mark Dawson, Louise Leach, Nicola Elliot, Paul Williams, Luke Rogers, Alison Bernard and others over the life course of the trial and to all of the individuals who support these services to function.

Contributors

Authorship follows ICMJE recommendations. JH is the trial manager and wrote the final protocol and protocol paper. GD is the chief investigator and has overall responsibility for the trial design and drafted the initial trial protocol. GD and CF are the joint study leads and lead on supervision and trial decisions. RD, BA, CA, NB, TB, LD, CG, and JS contributed to the study design. GD and CF led the development of the treatment with substantial input from a range of clinicians and service users. CG was responsible for patient and public involvement. JE and EK are responsible for the statistical analysis design. NB, JS, and LB coordinated the trial at sites and were responsible for recruitment. All authors read and approved the final trial protocol.

Funding

This project is funded by the National Institute for Health and Care Research (NIHR) under its Research for Patient Benefit (RfPB) Programme (Grant Reference Number NIHR204125). The views expressed are those of the author(s) and not necessarily those of the NIHR or the Department of Health and Social Care.

Competing interests

GD, RD, JS, NB and CA provide psychological therapies for individuals with psychosis in NHS settings. RD receives payment for workshops in treating hallucinations. GD, RD, CF, NB, and CA hold or have held grants to develop and carry out trials of psychological therapy for individuals with psychosis. LD provides sponsorship oversight of trials in CNTW NHS FT. All other authors declare no competing interest. The MUSE toolkit is the joint intellectual property of Durham University and Cumbria, Northumberland, Tyne and Wear NHS Foundation Trust and will be made freely available if proven to be beneficial.

Patient consent for publication

Not applicable.

Provenance and peer review

Not commissioned; peer reviewed for ethical and funding approval prior to submission.

For peer review only

Open access

This is an open access article distributed in accordance with the Creative Commons Attribution 4.0 Unported (CC BY 4.0) license, which permits others to copy, redistribute, remix, transform and build upon this work for any purpose, provided the original work is properly cited, a link to the licence is given, and indication of whether changes were made. See: https://creativecommons.org/licenses/by/4.0/.

References

1. Salazar de Pablo G, Radua J, Pereira J, Bonoldi I, Arienti V, Besana F, et al. Probability of Transition to Psychosis in Individuals at Clinical High Risk An Updated Meta-analysis. JAMA Psychiatry. 2021;78:970-8.
2. Salazar De Pablo G, Estradé A, Cutroni M, Andlauer O, Fusar-Poli P. Establishing a clinical service to prevent psychosis: What, how and when? Systematic review. Translational Psychiatry. 2021;11(1).
3. Yung AR, Wood SJ, Malla A, Nelson B, McGorry P, Shah J. The reality of at risk mental state services: a response to recent criticisms. Psychological Medicine. 2021;51(2):212-8.
4. Toh WL, Moseley P, Fernyhough C. Hearing voices as a feature of typical and psychopathological experience. Nature Reviews Psychology. 2022;1(2):72-86.
5. Nelson B, Yung A, Markulev C, Nicoll M. The CAARMS: assessing young people at ultra high risk of psychosis: Orygen Youth Health Research Centre; 2014.
6. Yung AR, Yuen HP, McGorry PD, Phillips LJ, Kelly D, Dell'Olio M, et al. Mapping the onset of psychosis: the Comprehensive Assessment of At-Risk Mental States. Aust N Z J Psychiatry 2005;39:964–71.
7. Dodgson G, Alderson-Day B, Smailes D, Ryles F, Mayer C, Glen-Davison J, et al. Tailoring cognitive behavioural therapy to subtypes of voice-hearing using a novel tabletised manual: a feasibility study. Behavioural and Cognitive Psychotherapy. 2021;49(3):287-301.
8. NICE. Psychosis and schizophrenia in adults: prevention and management. 2014:1-40.
9. NICE. Psychosis and schizophrenia. 2021.
10. NICE. Psychosis and schizophrenia in children and young people: recognition and management. 2013:1-48.
11. Bosnjak Kuharic D, Kekin I, Hew J, Rojnic Kuzman M, Puljak L. Interventions for prodromal stage of psychosis. Cochrane Database Syst Rev. 2019;2019(11).
12. Mei C, van der Gaag M, Nelson B, Smit F, Yuen HP, Berger M, et al. Preventive interventions for individuals at ultra high risk for psychosis: An updated and extended meta-analysis. Clinical psychology review. 2021;86:102005.
13. Stafford MR, Jackson H, Mayo-Wilson E, Morrison AP, Kendall T. Early interventions to prevent psychosis: systematic review and meta-analysis. BMJ. 2013;346(jan18 1):f185-f.
14. Fusar-Poli P, Davies C, Solmi M, Brondino N, De Micheli A, Kotlicka-Antczak M, et al. Preventive Treatments for Psychosis: Umbrella Review (Just the Evidence). Front Psychiatry. 2019;10:764.
15. van der Gaag M, van den Berg D, Ising H. CBT in the prevention of psychosis and other severe mental disorders in patients with an at risk mental state: A review and proposed next steps. Schizophrenia research. 2019;203:88-93.
16. Aceituno D, Vera N, Prina AM, McCrone P. Cost-effectiveness of early intervention in psychosis: systematic review. The British Journal of Psychiatry. 2019;215(01):388-94.

For peer review only

17. Wykes T, Bell A, Carr S, Coldham T, Gilbody S, Hotopf M, et al. Shared goals for mental health research: what, why and when for the 2020s. Journal of Mental Health. 2021:1-9.
18. Schmidt SJ, Schultze-Lutter F, Schimmelmann BG, Maric NP, Salokangas RK, Riecher-Rössler A, et al. EPA guidance on the early intervention in clinical high risk states of psychoses. Eur Psychiatry. 2015;30(3):388-404.
19. Addington J, Addington D, Abidi S, Raedler T, Remington G. Canadian Treatment Guidelines for Individuals at Clinical High Risk of Psychosis. Can J Psychiatry. 2017;62(9):656-61.
20. Drake RJ, Day CJ, Picucci R, Warburton J, Larkin W, Husain N, et al. A naturalistic, randomized, controlled trial combining cognitive remediation with cognitive-behavioural therapy after first-episode non-affective psychosis. Psychol Med. 2014;44(9):1889-99.
21. Dodgson G, Aynsworth C, Mitrenga KJ, Gibbs C, Patton V, Fernyhough C, et al. Managing unusual sensory experiences: A feasibility trial in an At Risk Mental States for psychosis group. Psychology and Psychotherapy: Theory, Research and Practice. 2021;94(3):481-503.
22. McCarthy-Jones S, Thomas N, Strauss C, Dodgson G, Jones N, Woods A, et al. Better than mermaids and stray dogs? Subtyping auditory verbal hallucinations and its implications for research and practice. Schizophrenia bulletin. 2014;40 Suppl 4(Suppl 4):S275-84.
23. Goldman HH, Skodol AE, Lave TR. Revising axis V for DSM-IV: a review of measures of social functioning. Am J Psychiatry. 1992;149(9):1148-56.
24. Haddock G, McCarron J, Tarrier N, Faragher EB. Scales to measure dimensions of hallucinations and delusions: the psychotic symptom rating scales (PSYRATS). Psychol Med. 1999;29(4):879-89.
25. Woodward TS, Jung K, Hwang H, Yin J, Taylor L, Menon M, et al. Symptom dimensions of the psychotic symptom rating scales in psychosis: a multisite study. Schizophrenia bulletin. 2014;40 Suppl 4(Suppl 4):S265-74.
26. Zsido AN, Teleki SA, Csokasi K, Rozsa S, Bandi SA. Development of the short version of the spielberger state-trait anxiety inventory. Psychiatry Res. 2020;291:113223.
27. Spielberger CD. State-Trait Anxiety Inventory. The Corsini Encyclopedia of Psychology1983.
28. Spielberger CD, Gorsuch RL, Lushene RE. Manual for the State-Trait Anxiety Inventory1970.
29. Dalenberg C, Carlson E. Severity of Dissociative Symptoms—Adult (Brief Dissociative Experiences Scale [DES-B]—Modified) [Measurement instrument]. American Psychiatric Association; 2010.
30. Dalenberg C, Carlson E. Severity of Dissociative Symptoms—Child Age 11–17 (Brief Dissociative Experiences Scale [DES-B]—Modified) [Measurement instrument]. . American Psychiatric Association; 2010.
31. Kuznetsova OM, Tymofyeyev Y. Preserving the allocation ratio at every allocation with biased coin randomization and minimization in studies with unequal allocation. Statistics in Medicine. 2012;31(8):701-23.
32. Beecham J, Knapp M. Costing psychiatric interventions. In: Thornicroft G, editor. Measuring Mental Health Needs. 2nd ed: Gaskell; 2001. p. 200-24.
33. Kroenke K, Spitzer RL, Williams JB. The PHQ-9: validity of a brief depression severity measure. J Gen Intern Med. 2001;16(9):606-13.
34. Spitzer RL, Kroenke K, Williams JB, Löwe B. A brief measure for assessing generalized anxiety disorder: the GAD-7. Arch Intern Med. 2006;166(10):1092-7.
35. Keetharuth AD, Brazier J, Connell J, Bjorner JB, Carlton J, Taylor Buck E, et al. Recovering Quality of Life (ReQoL): a new generic self-reported outcome measure for use with people experiencing mental health difficulties. Br J Psychiatry. 2018;212(1):42-9.
36. Bastien CH, Vallières A, Morin CM. Validation of the Insomnia Severity Index as an outcome measure for insomnia research. Sleep Med. 2001;2(4):297-307.
37. Cloitre M, Shevlin M, Brewin CR, Bisson JI, Roberts NP, Maercker A, et al. The International Trauma Questionnaire: development of a self-report measure of ICD-11 PTSD and complex PTSD. Acta Psychiatr Scand. 2018;138(6):536-46.

For peer review only

38. Haselgruber A, Sölva K, Lueger-Schuster B. Symptom structure of ICD-11 Complex Posttraumatic Stress Disorder (CPTSD) in trauma-exposed foster children: examining the International Trauma Questionnaire - Child and Adolescent Version (ITQ-CA). Eur J Psychotraumatol. 2020;11(1):1818974.
39. Alderson-Day B, Mitrenga K, Wilkinson S, McCarthy-Jones S, Fernyhough C. The varieties of inner speech questionnaire – Revised (VISQ-R): Replicating and refining links between inner speech and psychopathology. Consciousness and Cognition. 2018;65:48-58.
40. Bentall RP, Slade PD. Reality testing and auditory hallucinations: a signal detection analysis. Br J Clin Psychol. 1985;24 (Pt 3):159-69.
41. Moseley P, Aleman A, Allen P, Bell V, Bless J, Bortolon C, et al. Correlates of Hallucinatory Experiences in the General Population: An International Multisite Replication Study. Psychological Science. 2021;32(7):1024-37.
42. Paulik G, Badcock JC, Maybery MT. Poor intentional inhibition in individuals predisposed to hallucinations. Cognitive Neuropsychiatry. 2007;12:457-70.
43. Campbell ML, Morrison AP. The relationship between bullying, psychotic-like experiences and appraisals in 14-16-year olds. Behav Res Ther. 2007;45(7):1579-91.
44. Fernyhough C, Bland K, Meins E, Coltheart M. Imaginary companions and young children's responses to ambiguous auditory stimuli: implications for typical and atypical development. J Child Psychol Psychiatry. 2007;48(11):1094-101.
45. Andrade J, May J, Deeprose C, Baugh SJ, Ganis G. Assessing vividness of mental imagery: The Plymouth Sensory Imagery Questionnaire. Br J Psychol. 2014;105(4):547-63.
46. Smailes D, Burdis E, Gregoriou C, Fenton B, Dudley R. Pareidolia-proneness, reality discrimination errors, and visual hallucination-like experiences in a non-clinical sample. Cognitive Neuropsychiatry. 2020;25:113-25.
47. Aynsworth C, Nemat N, Collerton D, Smailes D, Dudley R. Reality monitoring performance and the role of visual imagery in visual hallucinations. Behaviour Research and Therapy. 2017;97:115-22.
48. Oei TP, Shuttlewood GJ. Development of a Satisfaction with Therapy and Therapist Scale. Aust N Z J Psychiatry. 1999;33(5):748-53.
49. Oei TPS, Green AL. The Satisfaction With Therapy and Therapist Scale--Revised (STTS-R) for group psychotherapy: Psychometric properties and confirmatory factor analysis. Professional Psychology: Research and Practice. 2008;39(4):435-42.
50. Dudley R, Dodgson G, Common S, O'Grady L, Watson F, Gibbs C, et al. Managing Unusual Sensory Experiences in People with First-Episode Psychosis (MUSE FEP): a study protocol for a single-blind parallel-group randomised controlled feasibility trial. BMJ Open. 2022;12(5):e061827.
51. Higgins JPT, Green S. Cochrane handbook for systematic reviews of interventions: John Wiley & Sons; 2011.
52. Schulz KF, Altman DG, Moher D, the CG. CONSORT 2010 Statement: updated guidelines for reporting parallel group randomised trials. BMC Medicine. 2010;8(1):18.
53. Teare MD, Dimairo M, Shephard N, Hayman A, Whitehead A, Walters SJ. Sample size requirements to estimate key design parameters from external pilot randomised controlled trials: a simulation study. Trials. 2014;15(1):264.
54. Braun V, Clarke V. Using thematic analysis in psychology. Qualitative research in psychology. 2006;3:77-101.

FIGURE TITLE

Figure 1. Data to report in line with the Consolidated Standards of Reporting Trials (CONSORT)

For peer review only

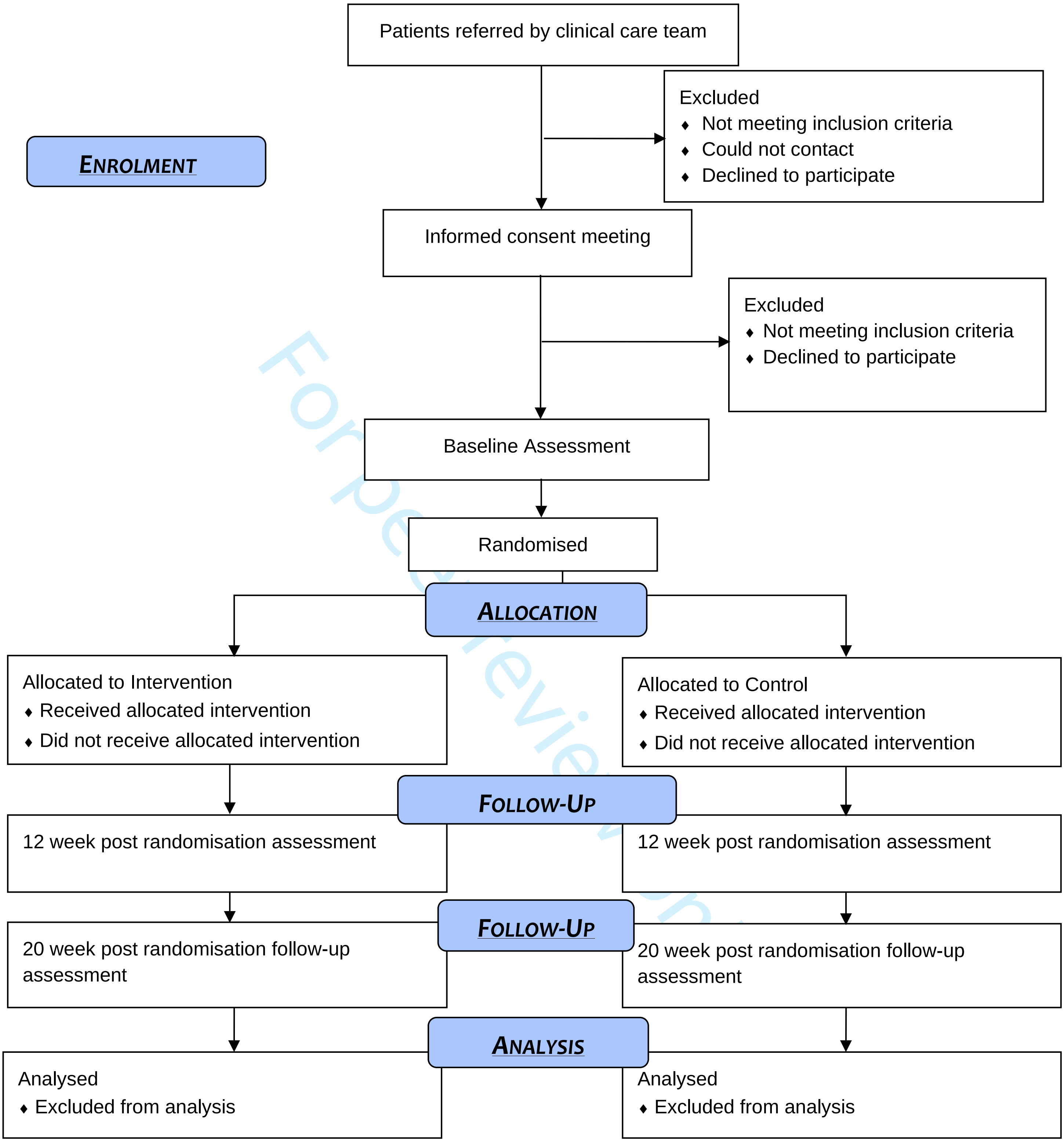
Patients referred by clinical care team
Excluded
Not meeting inclusion criteria
Could not contact
Declined to participate
ENROLMENT
Informed consent meeting
Excluded
Not meeting inclusion criteria
Declined to participate
Baseline Assessment
Randomised
ALLOCATION
Allocated to Intervention
Received allocated intervention
Did not receive allocated intervention
Allocated to Control
Received allocated intervention
Did not receive allocated intervention
FOLLOW-UP
12 week post randomisation assessment
12 week post randomisation assessment
FOLLOW-UP
20 week post randomisation follow-up assessment
20 week post randomisation follow-up assessment
ANALYSIS
Analysed
Excluded from analysis
Analysed
Excluded from analysis

Supplementary Table 1. Approved trial monitoring and progression criteria to a future definitive trial

Criterion	Critical feasibility outcome	Other feasibility and acceptability data relevant to the criterion		Proposed thresholds on critical outcome
**1) Recruitment**	• Number of participants consented into the trial and randomised	• Number of referrals per month<br>• Source of recruitment<br>• Number of participants eligible,<br>• Number of participants referred<br>• Reasons for non-eligibility or withdrawal of interest		Feasibility will be demonstrated where an average of least 7.84 participants are recruited and randomised per month (80% of recruitment target met).<br>If at least 5.88 participants are recruited per month, then a future trial will be feasible but additional strategies must be identified to support recruitment (e.g. informed by other feasibility data relevant to this criterion) (60-80% of recruitment target met).<br>If an average of under 5.88 participant is recruited per month over the recruitment period, feasibility within the current design will not be demonstrated (under 60% of recruitment target met).
**2) Therapy engagement**	• % who drop-out of therapy	• Session record forms for each therapy session<br>• Number of therapy sessions attended<br>• Qualitative interviews with SU participants<br>• Therapy satisfaction scores		Feasibility will be demonstrated if at least 80% of the participants in the intervention arm completed at least 4 out of the 6-8 sessions of MUSE.<br>If 60-80% of participants in the intervention arm complete at least 4 out of the 6-8 sessions of MUSE.<br>If less than 60% of participants in the intervention arm complete at least 4 out of the 6-8 sessions of MUSE.
**3) Assessment retention**	• % of participants who are lost to follow-up at primary assessment endpoint (12weeks post randomisation)	• Reasons for withdrawal from the study<br>• Qualitative interviews with SU participants<br>• Data completion		If at least 70% of participants complete primary outcome measure at primary assessment endpoint, feasibility will be demonstrated.<br>If 50-70% of participants complete primary outcome measure at primary assessment endpoint, a future trial will be feasible if strategies to overcome barriers are identified (e.g. via other data relevant to this).<br>If less than 50% of participants complete primary outcome measure at primary assessment endpoint, feasibility within the current design will not be demonstrated.
**4) Therapy fidelity**	• Adherence ratings from therapy tapes	• Session record form for each therapy session (including reasons for deviation from protocol)		Feasibility will be demonstrated if over 80% of rated therapy tapes are rated as acceptable.<br>If 50-80% of rated therapy tapes are rated as acceptable, a future trial will be feasible if strategies to overcome barriers are identified<br>If less than 50% of rated therapy tapes will be rates as acceptable, feasibility within the current design will not be demonstrated.
**5) Safety**	• Number of related SAEs	• Increased number of AEs in Intervention condition		0-1 Related SAEs in the Intervention arm.<br>2 Related SAEs in the Intervention arm.<br>3+ Related SAEs in the Intervention arm.

For peer review only

For peer review only

MUSE ARMS Feasibility Trial: Preferences Questionnaire; v1.0, 12.03.2023
IRAS Project ID: 323903

Preferences for psychological therapy or support

This set of questions is seeking to develop an understanding of your preferences for therapy treatment.

1. Do you have a preference for the number of therapy sessions you might get?

◯ 1-3

◯ 4-8

◯ 9-16

◯ 17-30

◯ Don't know

2. How important is it that your treatment:

Includes being give medication?

◯ Not important

◯ Somewhat important

◯ Very important

Includes a talking therapy?

◯ Not important

◯ Somewhat important

◯ Very important

Addresses any feelings of anxiety?

◯ Not important

◯ Somewhat important

◯ Very important

For peer review only - http://bmjopen.bmj.com/site/about/guidelines.xhtml

MUSE ARMS Feasibility Trial: Preferences Questionnaire; v1.0, 12.03.2023
IRAS Project ID: 323903

Addresses any feeling of low mood?

- ◯ Not important
- ◯ Somewhat important
- ◯ Very important

Helps you understand the causes of any unusual sensory experiences, such as hearing a voice?

- ◯ Not important
- ◯ Somewhat important
- ◯ Very important

Helps you learn to manage any unusual sensory experiences?

- ◯ Not important
- ◯ Somewhat important
- ◯ Very important

Helps you feel less distressed about any unusual sensory experiences?

- ◯ Not important
- ◯ Somewhat important
- ◯ Very important

For peer review only

3. What are your preferences for the way the therapist/clinical care team works with you? Please rate how important you think the following statements are:

I am given space to talk and feel heard

- ◯ Not important
- ◯ Somewhat important
- ◯ Very important

For peer review only - http://bmjopen.bmj.com/site/about/guidelines.xhtml

MUSE ARMS Feasibility Trial: Preferences Questionnaire; v1.0, 12.03.2023
IRAS Project ID: 323903

I work with my therapist to help me make sense of my experiences

- ◯ Not important
- ◯ Somewhat important
- ◯ Very important

I am involved in setting my own goals

- ◯ Not important
- ◯ Somewhat important
- ◯ Very important

I am given new ideas of how to cope with my experiences

- ◯ Not important
- ◯ Somewhat important
- ◯ Very important

4. How much do you hope to get the MUSE therapy?

- ◯ I would prefer to be allocated to MUSE based therapy
- ◯ I don't mind one way or the other whether I receive MUSE based therapy
- ◯ I would prefer to be allocated to the treatment as usual.

For peer review only

MUSE THERAPY THERAPIST PACK

*Return to research site file for archiving after completion

Adherence Checklist: MUSE Therapy Sessions
(Please tick topic used in any session)

**Insert Date**:									
**Insert length of session (minutes):**									
**Module/Topic**	**S1**	**S2**	**S3**	**S4**	**S5**	**S6**	**S7**	**S8**	**Comments**
**What are voices?**									
What are voices?									
How many people hear voices?									
Why does it become a problem?									
Can things get better?									
Personal experiences									
1. **How the mind works?**									
Thoughts and senses									
How thoughts work									
Embarrassing thoughts									
The power of attention									
How we use expectation									
2. **Assessment**									
Types of unusual sensory experiences.									
What kind of voices do we hear?									
3. **Inner Speech**									
What is inner speech?									
Our inner speech can do amazing things									
Why do people not recognise voices?									
Thoughts are hard to control									
Blocking the loop									
Inner speech – what is the evidence?									
Tracking the self – Was that me?									
Writers and voice hearing									
Imaginary friends									
Formulation									
Voices and Relationships									
Transforming the voice									
Testing out your explanations									

Completed by

Participant ID (MUSE ID Number):	Print Name:	Role:	Signature:	**Completed on (DD/MM/YYYY)**

For peer review only

Living well with voices									
4. **Memory Based Voices**									
Memory, dissociation, trauma									
The importance of trauma									
Threat system and Soothing system									
Formulation									
Treating trauma									
5. **Hypervigilance**									
Nature versus Nurture									
Filling in the gaps									
What our perception system is designed to do									
Response to danger									
Formulation									
Threat system and soothing system									
Mistrust									
6. **Seeing Visions**									
Is seeing believing?									
What do your visions mean to you?									
Perception system design									
Filling in the gaps									
Tracking the self – was that me?									
Imaginary friends									
Testing distressing appraisals									
Changing images									
Living well with visual experiences									
Voices, visions and relationships									
Challenging unacceptability									
Testing out your explanations									
Living well with voices and visions									
7. **Sleep**									
Why do we sleep?									
How to sleep well									

	**Completed by**			
Participant ID (MUSE ID Number):	Print Name:	Role:	Signature:	**Completed on (DD/MM/YYYY)**

For peer review only

Adverse Events Checklist

Insert date:									
	**S1**	**S2**	**S3**	**S4**	**S5**	**S6**	**S7**	**S8**	**Comments**
**Adverse events of interest reported?** **Add to Rio/Paris**									
**Serious Adverse Event? NB 24hour reporting deadline**									
**Urgent Safety Measures? NB Phone PI immediately**									

Adverse Events Guidance:

Adverse Events. Record on Rio/Paris for collection by the Unblinded Researcher at the 12wk and 20wk assessment time points that pertain to the following events of Protocol Interest:

- Clinically significant increases in distress and/or psychosis
- Increased harm to self/harm to others
- Increased suicidal ideation/attempts
- Increased use of drugs/alcohol
- Emergency room visits for mental health concerns
- Access to crises services

Serious Adverse Event (SAE): The site Principal Investigator (PI), or delegate shall report all SAEs within 24 hours of becoming aware of the event to the Chief Investigator (CI), or delegate via email to MUSE.ARMS@cntw.nhs.uk using the SAE reporting form. These are events that:

- results in death;
- is life-threatening;
- requires hospitalisation or prolongation of existing hospitalisation;
- results in persistent or significant disability or incapacity;
- consists of a congenital anomaly or birth defect; or,
- is otherwise considered medically significant by the investigator.

Urgent Safety Measures: The site Principal Investigator (PI), or delegate, must inform the CI immediately by telephone (Tel. 01670844670 / alternatively Teams video/voice call for guy.dodgson@cntw.nhs.uk) of urgent safety measures defined above in section 11.5 (early withdrawal/changes to procedure due to safety concerns for staff or participants). This information shall be documented on the Urgent safety reporting form and submitted by email to MUSE.ARMS@cntw.nhs.uk. This is when the following applies:

- Early withdrawal of participant(s) due to safety concerns about the intervention or assessments
- Changes to procedures due to concerns about staff or participant safety

Transition to Psychosis Checklist

Insert date:									
	**S1**	**S2**	**S3**	**S4**	**S5**	**S6**	**S7**	**S8**	**Comments**
**Indication of Transition to Psychosis?** **Add note to Rio/Paris**									

Transition to Psychosis Guidance: The following information suggests potential transition to psychosis for this protocol:

- Clinical diagnosis using standard diagnostic classification systems DSM/ICD
- Clinical diagnosis using ARMS assessment schedule documented in clinical notes
- Transfer to the Early Intervention in Psychosis pathway
- Treated or untreated psychotic episode of one week's duration or longer
- Initiation of treatment with antipsychotics (3 or more weeks of treatment with antipsychotics at a dose of ≥ 5mg haloperidol or equivalent)

	**Completed by**			
Participant ID (MUSE ID Number):	Print Name:	Role:	Signature:	**Completed on (DD/MM/YYYY)**

For peer review only

Therapy Session measure

MUSE Therapist Pack MUSE ARMS Feasibility Trial; IRAS ID: 323903

Session Number:

Date:

Therapy Session measure

Please answer the following questions about the voices you experienced <u>in the past week</u>

<u>How frequent were the voices?</u>

0%	10	20	30	40	50	60	70	80	90	100%
Voices not present					Once a day					Voices always present

<u>Were the voices distressing? How much of the time?</u>

0%	10	20	30	40	50	60	70	80	90	100%
Voices never distressing					Voices were distressing about half of the times					Voices always distressing

If relevant please answer the following questions about the visions you experienced <u>in the past week</u>

<u>How frequent were the visions?</u>

0%	10	20	30	40	50	60	70	80	90	100%
Visions not present					Once a day					Visions always present

<u>Were the vision distressing? How much of the time?</u>

0%	10	20	30	40	50	60	70	80	90	100%
Visions never distressing					Moderately distressing					extremely distressing

<u>Overall, how distressing were the experiences listed above?</u>

0%	10	20	30	40	50	60	70	80	90	100%
not at all distressing					Moderately distressing					extremely distressing

Completed by

Participant ID (MUSE ID Number):	Print Name:	Role:	Signature:	**Completed on (DD/MM/YYYY)**

For peer review only

Therapy Session measure

MUSE Therapist Pack MUSE ARMS Feasibility Trial; IRAS ID: 323903

Session Number:

Date:

Therapy Session measure

Please answer the following questions about the voices you experienced <u>in the past week</u>

<u>How frequent were the voices?</u>

0%	10	20	30	40	50	60	70	80	90	100%
Voices not present					Once a day					Voices always present

<u>Were the voices distressing? How much of the time?</u>

0%	10	20	30	40	50	60	70	80	90	100%
Voices never distressing					Voices were distressing about half of the times					Voices always distressing

If relevant please answer the following questions about the visions you experienced <u>in the past week</u>

<u>How frequent were the visions?</u>

0%	10	20	30	40	50	60	70	80	90	100%
Visions not present					Once a day					Visions always present

<u>Were the vision distressing? How much of the time?</u>

0%	10	20	30	40	50	60	70	80	90	100%
Visions never distressing					Moderately distressing					extremely distressing

<u>Overall, how distressing were the experiences listed above?</u>

0%	10	20	30	40	50	60	70	80	90	100%
not at all distressing					Moderately distressing					extremely distressing

Completed by

Participant ID (MUSE ID Number):	Print Name:	Role:	Signature:	**Completed on (DD/MM/YYYY)**

For peer review only

Therapy Session measure

MUSE Therapist Pack MUSE ARMS Feasibility Trial; IRAS ID: 323903

Session Number:

Date:

Therapy Session measure

Please answer the following questions about the voices you experienced <u>in the past week</u>

<u>How frequent were the voices?</u>

0%	10	20	30	40	50	60	70	80	90	100%
Voices not present					Once a day					Voices always present

<u>Were the voices distressing? How much of the time?</u>

0%	10	20	30	40	50	60	70	80	90	100%
Voices never distressing					Voices were distressing about half of the times					Voices always distressing

If relevant please answer the following questions about the visions you experienced <u>in the past week</u>

<u>How frequent were the visions?</u>

0%	10	20	30	40	50	60	70	80	90	100%
Visions not present					Once a day					Visions always present

<u>Were the vision distressing? How much of the time?</u>

0%	10	20	30	40	50	60	70	80	90	100%
Visions never distressing					Moderately distressing					extremely distressing

<u>Overall, how distressing were the experiences listed above?</u>

0%	10	20	30	40	50	60	70	80	90	100%
not at all distressing					Moderately distressing					extremely distressing

Completed by

Participant ID (MUSE ID Number):	Print Name:	Role:	Signature:	**Completed on (DD/MM/YYYY)**

For peer review only

Therapy Session measure

MUSE Therapist Pack MUSE ARMS Feasibility Trial; IRAS ID: 323903

Session Number:

Date:

Therapy Session measure

Please answer the following questions about the voices you experienced <u>in the past week</u>

<u>How frequent were the voices?</u>

0%	10	20	30	40	50	60	70	80	90	100%
Voices not present					Once a day					Voices always present

<u>Were the voices distressing? How much of the time?</u>

0%	10	20	30	40	50	60	70	80	90	100%
Voices never distressing					Voices were distressing about half of the times					Voices always distressing

If relevant please answer the following questions about the visions you experienced <u>in the past week</u>

<u>How frequent were the visions?</u>

0%	10	20	30	40	50	60	70	80	90	100%
Visions not present					Once a day					Visions always present

<u>Were the vision distressing? How much of the time?</u>

0%	10	20	30	40	50	60	70	80	90	100%
Visions never distressing					Moderately distressing					extremely distressing

<u>Overall, how distressing were the experiences listed above?</u>

0%	10	20	30	40	50	60	70	80	90	100%
not at all distressing					Moderately distressing					extremely distressing

Completed by

Participant ID (MUSE ID Number):	Print Name:	Role:	Signature:	**Completed on (DD/MM/YYYY)**

For peer review only

Therapy Session measure

MUSE Therapist Pack MUSE ARMS Feasibility Trial; IRAS ID: 323903

Session Number:

Date:

Therapy Session measure

Please answer the following questions about the voices you experienced in the past week

How frequent were the voices?

0%	10	20	30	40	50	60	70	80	90	100%
Voices not present					Once a day					Voices always present

Were the voices distressing? How much of the time?

0%	10	20	30	40	50	60	70	80	90	100%
Voices never distressing					Voices were distressing about half of the times					Voices always distressing

If relevant please answer the following questions about the visions you experienced in the past week

How frequent were the visions?

0%	10	20	30	40	50	60	70	80	90	100%
Visions not present					Once a day					Visions always present

Were the vision distressing? How much of the time?

0%	10	20	30	40	50	60	70	80	90	100%
Visions never distressing					Moderately distressing					extremely distressing

Overall, how distressing were the experiences listed above?

0%	10	20	30	40	50	60	70	80	90	100%
not at all distressing					Moderately distressing					extremely distressing

Completed by

Participant ID (MUSE ID Number):	Print Name:	Role:	Signature:	**Completed on (DD/MM/YYYY)**

For peer review only

Therapy Session measure

MUSE Therapist Pack MUSE ARMS Feasibility Trial; IRAS ID: 323903

Session Number:

Date:

Therapy Session measure

Please answer the following questions about the voices you experienced <u>in the past week</u>

<u>How frequent were the voices?</u>

0%	10	20	30	40	50	60	70	80	90	100%
Voices not present					Once a day					Voices always present

<u>Were the voices distressing? How much of the time?</u>

0%	10	20	30	40	50	60	70	80	90	100%
Voices never distressing					Voices were distressing about half of the times					Voices always distressing

If relevant please answer the following questions about the visions you experienced <u>in the past week</u>

<u>How frequent were the visions?</u>

0%	10	20	30	40	50	60	70	80	90	100%
Visions not present					Once a day					Visions always present

<u>Were the vision distressing? How much of the time?</u>

0%	10	20	30	40	50	60	70	80	90	100%
Visions never distressing					Moderately distressing					extremely distressing

<u>Overall, how distressing were the experiences listed above?</u>

0%	10	20	30	40	50	60	70	80	90	100%
not at all distressing					Moderately distressing					extremely distressing

Completed by

Participant ID (MUSE ID Number):	Print Name:	Role:	Signature:	**Completed on (DD/MM/YYYY)**

For peer review only

Therapy Session measure

MUSE Therapist Pack MUSE ARMS Feasibility Trial; IRAS ID: 323903

Session Number:

Date:

Therapy Session measure

Please answer the following questions about the voices you experienced <u>in the past week</u>

<u>How frequent were the voices?</u>

0%	10	20	30	40	50	60	70	80	90	100%
Voices not present					Once a day					Voices always present

<u>Were the voices distressing? How much of the time?</u>

0%	10	20	30	40	50	60	70	80	90	100%
Voices never distressing					Voices were distressing about half of the times					Voices always distressing

If relevant please answer the following questions about the visions you experienced <u>in the past week</u>

<u>How frequent were the visions?</u>

0%	10	20	30	40	50	60	70	80	90	100%
Visions not present					Once a day					Visions always present

<u>Were the vision distressing? How much of the time?</u>

0%	10	20	30	40	50	60	70	80	90	100%
Visions never distressing					Moderately distressing					extremely distressing

<u>Overall, how distressing were the experiences listed above?</u>

0%	10	20	30	40	50	60	70	80	90	100%
not at all distressing					Moderately distressing					extremely distressing

Completed by

Participant ID (MUSE ID Number):	Print Name:	Role:	Signature:	**Completed on (DD/MM/YYYY)**

For peer review only

Therapy Session measure

MUSE Therapist Pack MUSE ARMS Feasibility Trial; IRAS ID: 323903

Session Number:

Date:

Therapy Session measure

Please answer the following questions about the voices you experienced <u>in the past week</u>

<u>How frequent were the voices?</u>

0%	10	20	30	40	50	60	70	80	90	100%
Voices not present					Once a day					Voices always present

<u>Were the voices distressing? How much of the time?</u>

0%	10	20	30	40	50	60	70	80	90	100%
Voices never distressing					Voices were distressing about half of the times					Voices always distressing

If relevant please answer the following questions about the visions you experienced <u>in the past week</u>

<u>How frequent were the visions?</u>

0%	10	20	30	40	50	60	70	80	90	100%
Visions not present					Once a day					Visions always present

<u>Were the vision distressing? How much of the time?</u>

0%	10	20	30	40	50	60	70	80	90	100%
Visions never distressing					Moderately distressing					extremely distressing

<u>Overall, how distressing were the experiences listed above?</u>

0%	10	20	30	40	50	60	70	80	90	100%
not at all distressing					Moderately distressing					extremely distressing

	**Completed by**			
Participant ID (MUSE ID Number):	Print Name:	Role:	Signature:	**Completed on (DD/MM/YYYY)**

For peer review only

TAU THERAPY THERAPIST PACK

*Return to research site file for archiving after completion

Adherence Checklist: TAU Therapy Sessions
(Please tick for used in any session)

**Insert Date:**									
**Insert length of session (minutes):**									
	**S1**	**S2**	**S3**	**S4**	**S5**	**S6**	**S7**	**S8**	**Comments**
**Was this a CBT session (Y/N)?**									
CBT Assessment									
Formulation									
Needs based emotional support									
Social Support									
Normalisation									
Stress management									
Psychoeducation*<br><br>**Please describe if related to managing unusual sensory experiences in the comments box*									
Other:									
Other:									
Other:									

Completed by

Participant ID (MUSE ID Number):	Print Name:	Role:	Signature:	**Completed on (DD/MM/YYYY)**

For peer review only

Adverse Events Checklist

Insert date:									
	S1	S2	S3	S4	S5	S6	S7	S8	Comments
**Adverse events of interest reported?** **Add to Rio/Paris**									
**Serious Adverse Event? NB 24hour reporting deadline**									
**Urgent Safety Measures? NB Phone PI immediately**									

Adverse Events Guidance:

Adverse Events. Record on Rio/Paris for collection by the Unblinded Researcher at the 12wk and 20wk assessment time points that pertain to the following events of Protocol Interest:

- Clinically significant increases in distress and/or psychosis
- Increased harm to self/harm to others
- Increased suicidal ideation/attempts
- Increased use of drugs/alcohol
- Emergency room visits for mental health concerns
- Access to crises services

Serious Adverse Event (SAE): The site Principal Investigator (PI), or delegate shall report all SAEs within 24 hours of becoming aware of the event to the Chief Investigator (CI), or delegate via email to MUSE.ARMS@cntw.nhs.uk using the SAE reporting form. These are events that:

- results in death;
- is life-threatening;
- requires hospitalisation or prolongation of existing hospitalisation;
- results in persistent or significant disability or incapacity;
- consists of a congenital anomaly or birth defect; or,
- is otherwise considered medically significant by the investigator.

Urgent Safety Measures: The site Principal Investigator (PI), or delegate, must inform the CI immediately by telephone (Tel. 01670844670 / alternatively Teams video/voice call for guy.dodgson@cntw.nhs.uk) of urgent safety measures defined above in section 11.5 (early withdrawal/changes to procedure due to safety concerns for staff or participants). This information shall be documented on the Urgent safety reporting form and submitted by email to MUSE.ARMS@cntw.nhs.uk. This is when the following applies:

- Early withdrawal of participant(s) due to safety concerns about the intervention or assessments
- Changes to procedures due to concerns about staff or participant safety

Transition to Psychosis Checklist

Insert date:									
	S1	S2	S3	S4	S5	S6	S7	S8	Comments
**Indication of Transition to Psychosis?** **Add note to Rio/Paris**									

Transition to Psychosis Guidance: The following information suggests potential transition to psychosis for this protocol:

- Clinical diagnosis using standard diagnostic classification systems DSM/ICD
- Clinical diagnosis using ARMS assessment schedule documented in clinical notes
- Transfer to the Early Intervention in Psychosis pathway
- Treated or untreated psychotic episode of one week's duration or longer
- Initiation of treatment with antipsychotics (3 or more weeks of treatment with antipsychotics at a dose of ≥ 5mg haloperidol or equivalent

	**Completed by**			
Participant ID (MUSE ID Number):	Print Name:	Role:	Signature:	**Completed on (DD/MM/YYYY)**

For peer review only

For peer review only

[Insert local site logos here]
IRAS Number: 323903 Study Title: MUSE ARMS Feasibility Trial.
Informed Consent Form [Version 2.0 23022023]
Centre Name: [e.g. CNTW / Other NHS Participating Organisation]

NHS Cumbria, Northumberland, Tyne and Wear NHS Foundation Trust

Participant ID Number:

MUSE ARMS Feasibility Trial

INFORMED CONSENT FORM

		**Initial box to agree**
1	I confirm that I have read the information sheet dated.................... (Version............) for the above study. I have had the opportunity to consider the information, ask questions and have had these answered satisfactorily.	☐
2	I understand that my participation is voluntary and that I am free to withdraw at any time without giving any reason, without my medical care or legal rights being affected.	☐
3	I understand that if I withdraw from the study, or need to be withdrawn due to becoming too unwell, the research team will keep the research data about me that they already have, and if you give consent to question 10 on this form they will continue to track long term outcomes via the MHSDS/medical notes unless you request that they do not.	☐
4	I understand that relevant sections of my medical notes and data collected during the study, may be looked at by responsible individuals from *[Research site]* and from the research Sponsor CNTW NHS Foundation Trust, their representatives and regulatory authorities for the purposes of this research study, which includes audit and monitoring for research quality assurance. I give permission for these individuals to have access to my records in accordance with this study participant information sheet and informed consent.	☐
5	I understand and agree that the information collected from me in the course of this study will be held and maintained by *[enter name of organisation(s) that will be storing the participant data]* and CNTW, and archived at *[enter name of organisation(s)]* and CNTW.	☐
6	I agree to my NHS Care Team being informed of my participation in the study.	☐
7	I agree for a brief summary of the research assessments and any treatment sessions to be shared with my clinical team (i.e. added into my NHS care notes).	☐
8	OPTIONAL: I consent to the use of audio recording of my treatment sessions to check the quality of the MUSE treatment. I understand recordings will follow NHS data security standards for storage and will be destroyed once they are checked for treatment quality. **Circle decision: YES / NO**	☐

For peer review only - http://bmjopen.bmj.com/site/about/guidelines.xhtml

Print double sided. When completed: One for participant, one for Investigator Site File, and one to be kept in medical notes.

9	OPTIONAL: I consent to take part in an interview about my experience in the trial. I recognise not everyone is asked to do this and that I can change my mind at any time. I am aware that these reflective interviews are audio recorded anonymously (using an ID code as identifier) and then transcribed during which any further potential personal identifying information is removed ahead of analysis of research findings. **Circle decision: YES / NO**	☐
10	OPTIONAL: I consent to my medical records being accessed by the central research team at CNTW to collect follow-up data from medical databases to look at long term outcomes including use of hospital inpatient services. Medical databases include Hospital Records, and the Mental Health Services Data Set (MHSDS). This requires a copy of my consent form and my NHS record number to be sent securely to CNTW for processing and storage in the trial master file, which I agree to. **Circle decision: YES / NO**	☐
11	I understand that the information collected about me will be used to support the writing up of research findings. The data in an anonymised format will be shared with researchers for this study who have a role in analysing and writing up data.	☐
12	I understand that in accordance with openness of data findings the anonymised data set from the study may be published in open access and or for wider research. My personal details will not be shared.	☐
13	I agree to take part in the above study.	☐
14	OPTIONAL: I would like to be contacted with end of study information on the trial and my preferred contact method is: email / post / text message *(circle as appropriate).* Contact details will be obtained from medical records. **Circle decision: YES / NO**	☐

For peer review only

**Name of Participant**	
**Signature of Participant**	**Date**
***I certify that the information provided was discussed in a language accessible to the participant. That they retained and understood the information for a sufficient period in order to weigh up their decision and communicate their decision regarding informed consent.**	
***Name of Researcher Obtaining Consent**	
***Signature of Researcher Obtaining Consent**	**Date**

Print double sided. When completed: One for participant, one for Investigator Site File, and one to be kept in medical notes.

For peer review only - http://bmjopen.bmj.com/site/about/guidelines.xhtml

[Insert local site logos here]
IRAS Number: Study Title: MUSE ARMS Feasibility Trial.
YP Assent Form [Version 2.0 23022023]
Centre Name: [e.g. CNTW / Other NHS / Participating Organisation]

Cumbria, Northumberland, Tyne and Wear NHS Foundation Trust

Participant ID Number:

MUSE ARMS Feasibility Trial

YP ASSENT FORM

		Initial box to agree
1	I confirm that I have had time to think about this study. I have had the time to consider the information, ask questions, and have had helpful answers.	☐
2	I understand that taking part is my choice. I am free to stop or take a break at any time without giving any reason.	☐
3	I understand that if I withdraw from the study, or need to be withdrawn due to becoming too unwell, the research team will keep the research data about me that they have already collected, and if you give assent to question 10 on this form they will continue to track long term outcomes via medical records unless you request that they do not.	☐
4	I understand that the research team will only collect information that helps answer the research questions.	☐
5	I understand that my medical notes and the information collected from me will be looked after by the NHS trusts involved in the study for research data quality checks.	☐
6	I agree to my NHS Care Team being told about of my participation in the study.	☐
7	I agree for a short summary of the research assessments and any treatment sessions to be shared with my clinical team (added into my NHS care notes).	☐
8	OPTIONAL: I agree to the audio recording of my treatment sessions. This is to check the treatment is being done properly and not what I am saying.<br>I understand recordings will follow NHS data security standards for storage and will be destroyed once they are checked.<br>**Circle decision: YES / NO**	☐

Print double sided. When completed: One for participant, one for Investigator Site File, and one to be kept in medical notes.

For peer review only - http://bmjopen.bmj.com/site/about/guidelines.xhtml

9	OPTIONAL: I agree to take part in an interview about my experience of the study*. These interviews are recorded confidentially and then written out; any identifying information is removed (it's anonymous). *Not everyone is asked to do this. **Circle decision: YES / NO**	☐
10	OPTIONAL : I agree to my medical records being accessed to collect data to look at long term outcomes including use of hospital inpatient services. This requires a copy of my assent form & parent/guardian consent form and my NHS record number to be sent securely to CNTW for processing and storage in the trial master file, which I agree to. **Circle decision: YES / NO**	☐
11	It has been explained that the information collected about me is anonymised (no one will know my name). The information collected is used by researchers for this study who have a job analysing and writing up the findings.	☐
12	It has been explained that the anonymised data set from the study may be published in open access for wider research. My personal details will not be shared (no one will know my name).	☐
13	I would like to take part in the study.	☐
14	OPTIONAL: I would like to be sent end of study information on how it went overall. My preferred contact method is: email / post / text message *(circle preferred).* Contact details will be obtained from medical records. **Circle decision: YES / NO**	☐

**Name of Participant**	
**Signature of Participant**	**Date**
I certify that the information provided was discussed in a language accessible to the participant. That they retained and understood the information for a sufficient period in order to weigh up their decision and communicate their decision.	
***Name of Researcher Obtaining Assent**	
***Signature of Researcher Obtaining Assent**	**Date**

Print double sided. When completed: One for participant, one for Investigator Site File, and one to be kept in medical notes.

For peer review only

NHS Cumbria, Northumberland, Tyne and Wear NHS Foundation Trust

[Insert local site logos here]
IRAS Number: 323903 Study Title: MUSE ARMS Feasibility Trial.
Parent/Guardian Consent Form [Version 2.0 23022023]
Centre Name: [e.g. CNTW / Other NHS Participating Organisation]

Participant ID Number:

MUSE ARMS Feasibility Trial

PARENT/GUARDIAN INFORMED CONSENT FORM

		Initial box to agree
1	I confirm that I have read the parent/guardian information sheet dated................... (Version............) for the above study. I have had the opportunity to consider the information, ask questions and have had these answered satisfactorily.	☐
2	I understand that my child's participation is voluntary and that they are free to withdraw at any time without giving any reason, without their medical care or legal rights being affected.	☐
3	I understand that if my child withdraws from the study, or needs to be withdrawn due to becoming too unwell, the research team will keep the research data about my child that they already have, and if you give consent to question 10 on this form they will continue to track long term outcomes via the MHSDS/medical notes unless you request that they do not.	☐
4	I understand that relevant sections of my child's medical notes and data collected during the study, may be looked at by responsible individuals from *[Research site]* and from the research Sponsor CNTW NHS Foundation Trust, their representatives and regulatory authorities for the purposes of this research study, which includes audit and monitoring for research quality assurance. I give permission for these individuals to have access to these records in accordance with this study participant information sheet and informed consent and my child's agreement (assent).	☐
5	I understand and agree that the information collected about my child in the course of this study will be held and maintained by *[enter name of organisation(s) that will be storing the participant data]*, and CNTW and archived at *[enter name of organisation(s)]* and CNTW.	☐
6	I agree to my child's NHS Care Team being informed of their participation in the study.	☐
7	I agree for a brief summary of the research assessments and any treatment sessions to be shared with my child's clinical team (i.e. added into NHS care notes).	☐
8	OPTIONAL: I consent to the use of audio recording of my child's treatment sessions, so long as my child agrees to this, to check the quality of the MUSE treatment. I understand recordings will follow NHS data security standards for storage and will be destroyed once they are checked for treatment quality. **Circle decision: YES / NO**	☐

Print double sided. When completed: One for participant, one for Investigator Site File, and one to be kept in medical notes.

For peer review only - http://bmjopen.bmj.com/site/about/guidelines.xhtml

9	OPTIONAL: I consent to my child to take part in an interview about their experience in the trial, if they wish to do this.<br>I recognise not everyone is asked to do this and that my child can change their mind at any time. I am aware that these reflective interviews are audio recorded anonymously (using an ID code as identifier) and then transcribed during which any further potential personal identifying information is removed ahead of analysis of research findings.<br>**Circle decision: YES / NO**	☐
10	I consent to my child's medical records being accessed to collect follow-up data from medical databases to look at long term outcomes including use of hospital inpatient services, so long as they are in agreement with this. Medical databases Hospital Records, and the Mental Health Services Data Set (MHSDS). This requires a copy of my child's consent form and NHS record number to be sent securely to CNTW for processing and storage in the trial master file, which I agree to.	☐
11	I understand that the information collected about my child will be used to support the writing up of research findings. The data in an anonymised format will be shared with researchers for this study who have a role in analysing and writing up data.	☐
12	I understand that in accordance with openness of data findings the anonymised data set from the study may be published in open access and or for wider research. My child's personal details will not be shared.	☐
13	I agree for my child to take part in the above study if they wish to do so.	☐
14	OPTIONAL: I agree for my child to be contacted with end of study information on the trial and their preferred contact method is: email / post / text message *(circle as appropriate).* Contact details will be obtained from medical records.<br>**Circle decision: YES / NO**	☐

**Name of Parent/Guardian**	
**Signature of Parent/Guardian**	**Date**
***I certify that the information provided was discussed in a language accessible to the Parent/Guardian. That they retained and understood the information for a sufficient period in order to weigh up their decision and communicate their decision regarding informed consent.**	
***Name of Researcher Obtaining Consent**	
***Signature of Researcher Obtaining Consent**	**Date**

Print double sided. When completed: One for parent/guardian, one for Investigator Site File, and one to be kept in medical notes.

For peer review only - http://bmjopen.bmj.com/site/about/guidelines.xhtml

SPIRIT

STANDARD PROTOCOL ITEMS: RECOMMENDATIONS FOR INTERVENTIONAL TRIALS

SPIRIT 2013 Checklist: Recommended items to address in a clinical trial protocol and related documents*

**Section/item**	**Item No**	**Description**	
**Administrative information**			
Title	1	Descriptive title identifying the study design, population, interventions, and, if applicable, trial acronym	✓
Trial registration	2a	Trial identifier and registry name. If not yet registered, name of intended registry	✓
	2b	All items from the World Health Organization Trial Registration Data Set	
Protocol version	3	Date and version identifier	✓
Funding	4	Sources and types of financial, material, and other support	✓
Roles and responsibilities	5a	Names, affiliations, and roles of protocol contributors	✓
	5b	Name and contact information for the trial sponsor	✓
	5c	Role of study sponsor and funders, if any, in study design; collection, management, analysis, and interpretation of data; writing of the report; and the decision to submit the report for publication, including whether they will have ultimate authority over any of these activities	✓
	5d	Composition, roles, and responsibilities of the coordinating centre, steering committee, endpoint adjudication committee, data management team, and other individuals or groups overseeing the trial, if applicable (see Item 21a for data monitoring committee)	✓

For peer review only

**Introduction**			
Background and rationale	6a	Description of research question and justification for undertaking the trial, including summary of relevant studies (published and unpublished) examining benefits and harms for each intervention	
	6b	Explanation for choice of comparators	✓
Objectives	7	Specific objectives or hypotheses	✓
Trial design	8	Description of trial design including type of trial (eg, parallel group, crossover, factorial, single group), allocation ratio, and framework (eg, superiority, equivalence, noninferiority, exploratory)	✓
**Methods: Participants, interventions, and outcomes**			
Study setting	9	Description of study settings (eg, community clinic, academic hospital) and list of countries where data will be collected. Reference to where list of study sites can be obtained	✓
Eligibility criteria	10	Inclusion and exclusion criteria for participants. If applicable, eligibility criteria for study centres and individuals who will perform the interventions (eg, surgeons, psychotherapists)	✓
Interventions	11a	Interventions for each group with sufficient detail to allow replication, including how and when they will be administered	✓
	11b	Criteria for discontinuing or modifying allocated interventions for a given trial participant (eg, drug dose change in response to harms, participant request, or improving/worsening disease)	
	11c	Strategies to improve adherence to intervention protocols, and any procedures for monitoring adherence (eg, drug tablet return, laboratory tests)	
	11d	Relevant concomitant care and interventions that are permitted or prohibited during the trial	
Outcomes	12	Primary, secondary, and other outcomes, including the specific measurement variable (eg, systolic blood pressure), analysis metric (eg, change from baseline, final value, time to event), method of aggregation (eg, median, proportion), and time point for each outcome. Explanation of the clinical relevance of chosen efficacy and harm outcomes is strongly recommended	

For peer review only - http://bmjopen.bmj.com/site/about/guidelines.xhtml

Participant timeline	13	Time schedule of enrolment, interventions (including any run-ins and washouts), assessments, and visits for participants. A schematic diagram is highly recommended (see Figure)	
Sample size	14	Estimated number of participants needed to achieve study objectives and how it was determined, including clinical and statistical assumptions supporting any sample size calculations	
Recruitment	15	Strategies for achieving adequate participant enrolment to reach target sample size	
**Methods: Assignment of interventions (for controlled trials)**			
Allocation:			
Sequence generation	16a	Method of generating the allocation sequence (eg, computer-generated random numbers), and list of any factors for stratification. To reduce predictability of a random sequence, details of any planned restriction (eg, blocking) should be provided in a separate document that is unavailable to those who enrol participants or assign interventions	
Allocation concealment mechanism	16b	Mechanism of implementing the allocation sequence (eg, central telephone; sequentially numbered, opaque, sealed envelopes), describing any steps to conceal the sequence until interventions are assigned	
Implementation	16c	Who will generate the allocation sequence, who will enrol participants, and who will assign participants to interventions	
Blinding (masking)	17a	Who will be blinded after assignment to interventions (eg, trial participants, care providers, outcome assessors, data analysts), and how	
	17b	If blinded, circumstances under which unblinding is permissible, and procedure for revealing a participant's allocated intervention during the trial	
**Methods: Data collection, management, and analysis**			
Data collection methods	18a	Plans for assessment and collection of outcome, baseline, and other trial data, including any related processes to promote data quality (eg, duplicate measurements, training of assessors) and a description of study instruments (eg, questionnaires, laboratory tests) along with their reliability and validity, if known. Reference to where data collection forms can be found, if not in the protocol	
	18b	Plans to promote participant retention and complete follow-up, including list of any outcome data to be collected for participants who discontinue or deviate from intervention protocols	

For peer review only - http://bmjopen.bmj.com/site/about/guidelines.xhtml

Data management	19	Plans for data entry, coding, security, and storage, including any related processes to promote data quality (eg, double data entry; range checks for data values). Reference to where details of data management procedures can be found, if not in the protocol	
Statistical methods	20a	Statistical methods for analysing primary and secondary outcomes. Reference to where other details of the statistical analysis plan can be found, if not in the protocol	
	20b	Methods for any additional analyses (eg, subgroup and adjusted analyses)	
	20c	Definition of analysis population relating to protocol non-adherence (eg, as randomised analysis), and any statistical methods to handle missing data (eg, multiple imputation)	
**Methods: Monitoring**			
Data monitoring	21a	Composition of data monitoring committee (DMC); summary of its role and reporting structure; statement of whether it is independent from the sponsor and competing interests; and reference to where further details about its charter can be found, if not in the protocol. Alternatively, an explanation of why a DMC is not needed	
	21b	Description of any interim analyses and stopping guidelines, including who will have access to these interim results and make the final decision to terminate the trial	
Harms	22	Plans for collecting, assessing, reporting, and managing solicited and spontaneously reported adverse events and other unintended effects of trial interventions or trial conduct	
Auditing	23	Frequency and procedures for auditing trial conduct, if any, and whether the process will be independent from investigators and the sponsor	
**Ethics and dissemination**			
Research ethics approval	24	Plans for seeking research ethics committee/institutional review board (REC/IRB) approval	
Protocol amendments	25	Plans for communicating important protocol modifications (eg, changes to eligibility criteria, outcomes, analyses) to relevant parties (eg, investigators, REC/IRBs, trial participants, trial registries, journals, regulators)	
Consent or assent	26a	Who will obtain informed consent or assent from potential trial participants or authorised surrogates, and how (see Item 32)	
	26b	Additional consent provisions for collection and use of participant data and biological specimens in ancillary studies, if applicable	

For peer review only - http://bmjopen.bmj.com/site/about/guidelines.xhtml

Confidentiality	27	How personal information about potential and enrolled participants will be collected, shared, and maintained in order to protect confidentiality before, during, and after the trial	
Declaration of interests	28	Financial and other competing interests for principal investigators for the overall trial and each study site	
Access to data	29	Statement of who will have access to the final trial dataset, and disclosure of contractual agreements that limit such access for investigators	Protocol paper Data management * See Note Below
Ancillary and post-trial care	30	Provisions, if any, for ancillary and post-trial care, and for compensation to those who suffer harm from trial participation	IRAS NHS Insurance * See Note Below
Dissemination policy	31a	Plans for investigators and sponsor to communicate trial results to participants, healthcare professionals, the public, and other relevant groups (eg, via publication, reporting in results databases, or other data sharing arrangements), including any publication restrictions	
	31b	Authorship eligibility guidelines and any intended use of professional writers	Protocol Paper – ICMJE * See Note Below
	31c	Plans, if any, for granting public access to the full protocol, participant-level dataset, and statistical code	
**Appendices**			
Informed consent materials	32	Model consent form and other related documentation given to participants and authorised surrogates	
Biological specimens	33	Plans for collection, laboratory evaluation, and storage of biological specimens for genetic or molecular analysis in the current trial and for future use in ancillary studies, if applicable	N/A

*It is strongly recommended that this checklist be read in conjunction with the SPIRIT 2013 Explanation & Elaboration for important clarification on the items. Amendments to the protocol should be tracked and dated. The SPIRIT checklist is copyrighted by the SPIRIT Group under the Creative Commons "Attribution-NonCommercial-NoDerivs 3.0 Unported" license.

For peer review only - http://bmjopen.bmj.com/site/about/guidelines.xhtml

*The SRIRIT checklist has been carefully reviewed against the trial protocol and associated documentation and the explanation of the protocol in the protocol paper submitted.

To provide further explanation on three items where more consideration could be given within the protocol itself are:

(i) item 29, refers to a statement of who will have access to the final trial dataset, and disclosure of contractual agreements that limit such access for investigators. This is an NIHR funded feasibility trial with no DMEC within its feasibility stage. We do not have contractual agreements that limit data access, other than to limit access to confidential information as this is restricted and explained in the participant facing documents. We have detailed in the protocol paper how data is input, stored, and transferred prior to analysis to allow for audit and monitoring in accordance with the trial monitoring plan.

(ii) item 30 refers to provisions, if any, for ancillary and post-trial care, and for compensation to those who suffer harm from trial participation. As this is a psychological therapies trial there are no biological risks, however we are conscious of the possibility of increased psychological risks and so we have developed clear safety reporting procedures for serious adverse events and for urgent safety measures should these arise, which abide by HRA and UK Research Ethics Committee standard procedures. We have also written into the protocol procedures for collecting adverse events that do not hit the seriousness criteria, so that these can be reported in the main outcomes paper. Our research has NHS insurance and this is clear on the IRAS application document. Any post-trial care would be standard NHS care;

(iii) item 31b refers to authorship eligibility guidelines and any intended use of professional writers. We do not use professional writers. We have not written authorship eligibility into the protocol, however the lead writers for the different papers have been identified in advance. We have followed the ICMJE recommendations for authorship and have noted this in the contributors section of the protocol paper.

All other items of the SPIRIT checklist are available within the protocol and Research Ethics Committee approved participant facing documents as appropriate.

For peer review only

CONSORT Abstract Checklist (Clarke et al 2008).

The Abstract has been reviewed in accordance with the CONSORT Abstract Checklist and is in adherence, see below. As this is a protocol paper of a trial open and in its very early stages of data collection, no results are currently available. TheMUSE ARMS Feasibility Trial opened to participants on 14.04.2023.

Item	Description	
Title	Identification of the study as randomised	✓
Authors*	Contact details for the corresponding author	✓
Trial design	Description of the trial design (eg, parallel, cluster, non-inferiority)	✓
Methods		
Participants	Eligibility criteria for participants and the settings where the data were collected	✓
Interventions	Interventions intended for each group	✓
Objective	Specific objective or hypothesis	✓
Outcome	Clearly defined primary outcome for this report	✓
Randomisation	How participants were allocated to interventions	✓
Blinding (masking)	Whether or not participants, care givers, and those assessing the outcomes were blinded to group assignment	✓
Results		
Numbers randomised	Number of participants randomised to each group	
Recruitment	Trial status	
Numbers analysed	Number of participants analysed in each group	
Outcome	For the primary outcome, a result for each group and the estimated effect size and its precision	
Harms	Important adverse events or side-effects	
Conclusions	General interpretation of the results	
Trial registration	Registration number and name of trial register	✓
Funding	Source of funding	✓

*For conference abstracts.

Reference:

CONSORT for reporting randomised trials in journal and conference abstracts
Clarke, Mike;Moher, David;Wager, Elizabeth;Middleton, Philippa;Altman, Douglas G;Schulz, Kenneth F
The Lancet; Jan 26-Feb 1, 2008; 371, 9609; ProQuest
pg. 281
