# Supplementary material for: Use of a targeted, computer/web-based guided self-help psychoeducation toolkit for distressing hallucinations (MUSE) in people with an at-risk mental state for psychosis: protocol for a randomised controlled feasibility trial
Source: BMJ Open. 2023 Jun 30;13(6):e076101. doi: 10.1136/bmjopen-2023-076101 (PMC10314705; doi:10.1136/bmjopen-2023-076101)
Supplement: Reviewer comments [file bmjopen-2023-076101.reviewer_comments.pdf]

PEER REVIEW HISTORY

BMJ Open publishes all reviews undertaken for accepted manuscripts. Reviewers are asked to complete a checklist review form (**http://bmjopen.bmj.com/site/about/resources/checklist.pdf**) and are provided with free text boxes to elaborate on their assessment. These free text comments are reproduced below.

ARTICLE DETAILS

**TITLE (PROVISIONAL)**	Use of a targeted, computer/web-based guided self-help psychoeducation toolkit for distressing hallucinations (MUSE) in people with an At Risk Mental State for psychosis: protocol for a randomised controlled feasibility trial
**AUTHORS**	Hamilton, Jahnese; Arnott, Bronia; Aynsworth, Charlotte; Barclay, Nicola; Birkett, Lauren; Brandon, Toby; Dixon, Lyndsey; Dudley, Robert; Einbeck, J; Gibbs, Christopher; Kharatikoopaei, Ehsan; Simpson, Jennifer; Dodgson, Guy; Fernyhough, Charles

This article was not externally reviewed at BMJ Open. Protocols that have been independently assessed prior to submission to BMJ Open are usually fast-tracked to publication on the grounds that further substantial changes will not be possible. This independent assessment will usually be external, independent review for both a substantial grant award from a non-commercial or government funder and ethics approval.
